# Statins promote bone anabolism in estrogen deficiency by inducing osteogenic differentiation of skeletal stem/progenitor cells

**DOI:** 10.1038/s43587-026-01257-1

**Published:** 2026-10-27

**Authors:** Min Yang, Jiaming Guo, Juan Ye, Xiangjiao Meng, Yunran Ma, Yuehan Ni, Tingyi Chen, Yixuan Zhang, Hanyao Guo, Ge Shen, Yi Yuan, Xinyi Qiao, Yifei Wu, Jiaojiao Li, Xiangbing Qi, Shentong Fang, Ling Wang, Bo Shen

**Affiliations:** 1 College of Life Sciences, Beijing Normal University, Beijing, China; 2 National Institute of Biological Sciences, Beijing (NIBS), Beijing, China; 3 Tsinghua Institute of Multidisciplinary Biomedical Research, Tsinghua University, Beijing, China; 4 Peking University-Tsinghua University-National Institute of Biological Sciences Joint Graduate Program, Academy for Advanced Interdisciplinary Studies, Peking University, Beijing, China; 5 Department of Radiology, Suzhou Kowloon Hospital, Shanghai Jiao Tong University School of Medicine, Suzhou, China; 6 Chinese Academy of Medical Sciences & Peking Union Medical College, Beijing, China; 7 Peking University-Tsinghua University-National Institute of Biological Sciences Joint Graduate Program, School of Life Sciences, Tsinghua University, Beijing, China; 8 Department of Radiology, Beijing Jishuitan Hospital, Capital Medical University, National Center for Orthopaedics, Beijing, China; 9 Zhili College, Tsinghua University, Beijing, China; 10 School of Biopharmacy, China Pharmaceutical University, Nanjing, China; 11 JST Sarcopenia Research Centre, National Center for Orthopaedics, Beijing Research Institute of Traumatology and Orthopaedics, Beijing Jishuitan Hospital, Capital Medical University, Beijing, China

**Keywords:** Ageing, Stem-cell differentiation

## Abstract

Menopause-associated estrogen deficiency drives age-related bone loss in women. Statin use has been associated with higher bone mineral density in postmenopausal women, but whether menopause alters skeletal responses to statins remains unclear. Here, we show that statin use was associated with a slower annual decline in bone mineral density in postmenopausal women and that atorvastatin promotes bone anabolism specifically under estrogen-deficient conditions by inducing osteogenic differentiation of leptin receptor-expressing (LEPR^+^) bone marrow stromal cells, which include skeletal stem/progenitor cells. Atorvastatin increases osteoblast formation by restricting RAC1 prenylation and enabling nuclear factor kappa B-mediated *Oln* transcription. Estrogen acts on the same LEPR^+^ cells through estradiol–estrogen receptor-α signaling to promote *Oln* transcription. Thus, statins compensate for reduced estradiol–estrogen receptor-α signaling through a mechanistically distinct pathway. These findings reposition a major component of estrogen action to an earlier osteogenic stage and suggest broader implications for how statins influence stem/progenitor cell responsiveness beyond cholesterol management.

## Abstract (web summary)

Yang et al. demonstrate that statins can compensate for reduced estrogen signaling in skeletal stem and progenitor cells, promoting bone anabolism during estrogen deficiency and suggesting therapeutic benefits beyond cholesterol management.

## Main

In women, estrogen deficiency during menopause leads to a characteristic pattern of skeletal decline, marked by reduced bone formation and accelerated bone loss^1^. This phenotype occurs naturally during menopause and can be mimicked by surgical or pharmacological suppression of ovarian function^1^. Although hormone replacement therapy is the principal treatment to counteract these effects, its use is limited by eligibility criteria, timing and risk factors, leaving many women untreated or intermittently treated^2,3^. Observational studies have reported that statin use is associated with higher bone mineral density (BMD)^4^ or reduced fracture risk in postmenopausal women^5^ and older populations^6,7^, although results have varied across cohorts, endpoints and study designs^5–8^.

Osteogenic differentiation is the process by which skeletal stem/progenitor cells give rise to osteoblasts and osteocytes^9–12^. This process has traditionally been studied mainly in osteoblasts and osteocytes^1^. However, LEPR^+^ bone marrow stromal cells, which include skeletal stem/progenitor cells, are positioned upstream in this trajectory and give rise to the majority of osteoblasts and osteocytes formed during adulthood^13–15^. Although estrogen signaling promotes osteogenic differentiation, most prior studies have examined bone surface-associated osteogenic progenitors and their downstream osteoblasts and osteocytes^16,17^, leaving its site of action within bone marrow skeletal stem/progenitor populations less well defined.

In this study, we investigated how estrogen deficiency shapes skeletal responses to statins at the level of adult stem/progenitor cells. By integrating two postmenopausal clinical cohorts with studies of aged female and ovariectomized mice, we found that statin use was associated with a slower annual decline in BMD and that atorvastatin promoted bone anabolism under estrogen-deficient conditions. These skeletal effects were not explained by low-density lipoprotein (LDL) lowering. Inducible fate mapping showed that statins act on LEPR^+^ cells to promote bone formation, and estradiol (E2) likewise promoted osteogenic differentiation in the same population, positioning estrogen action earlier in the osteogenic trajectory than previously appreciated. Mechanistically, estradiol–estrogen receptor-α (E2–ERα) signaling promoted transcription of *Oln/Clec11a*, which encodes the osteogenic growth factor osteolectin (OLN), whereas statins restricted RAC1 prenylation, increased RAC1 activation and enabled nuclear factor kappa B (NF-κB) recruitment to the *Oln* locus, and that OLN was required for statin-induced bone anabolism.

## Results

### Statins attenuate bone loss in postmenopausal women and estrogen-deficient mice

With the cessation of ovarian estrogen production at menopause, circulating levels of E2 decline^1^, leading to increased bone resorption and reduced bone formation, resulting in net bone loss^18^. Estrogen deficiency is a defining physiological feature of menopause and affects virtually all postmenopausal women; it is therefore considered the dominant pathophysiologic driver of osteoporosis^18^. Previous studies have reported that widely prescribed statins promote osteoblast function to form bone^19^ and are associated with higher BMD in postmenopausal women with estrogen deficiency^4^.

**Fig. 1:**
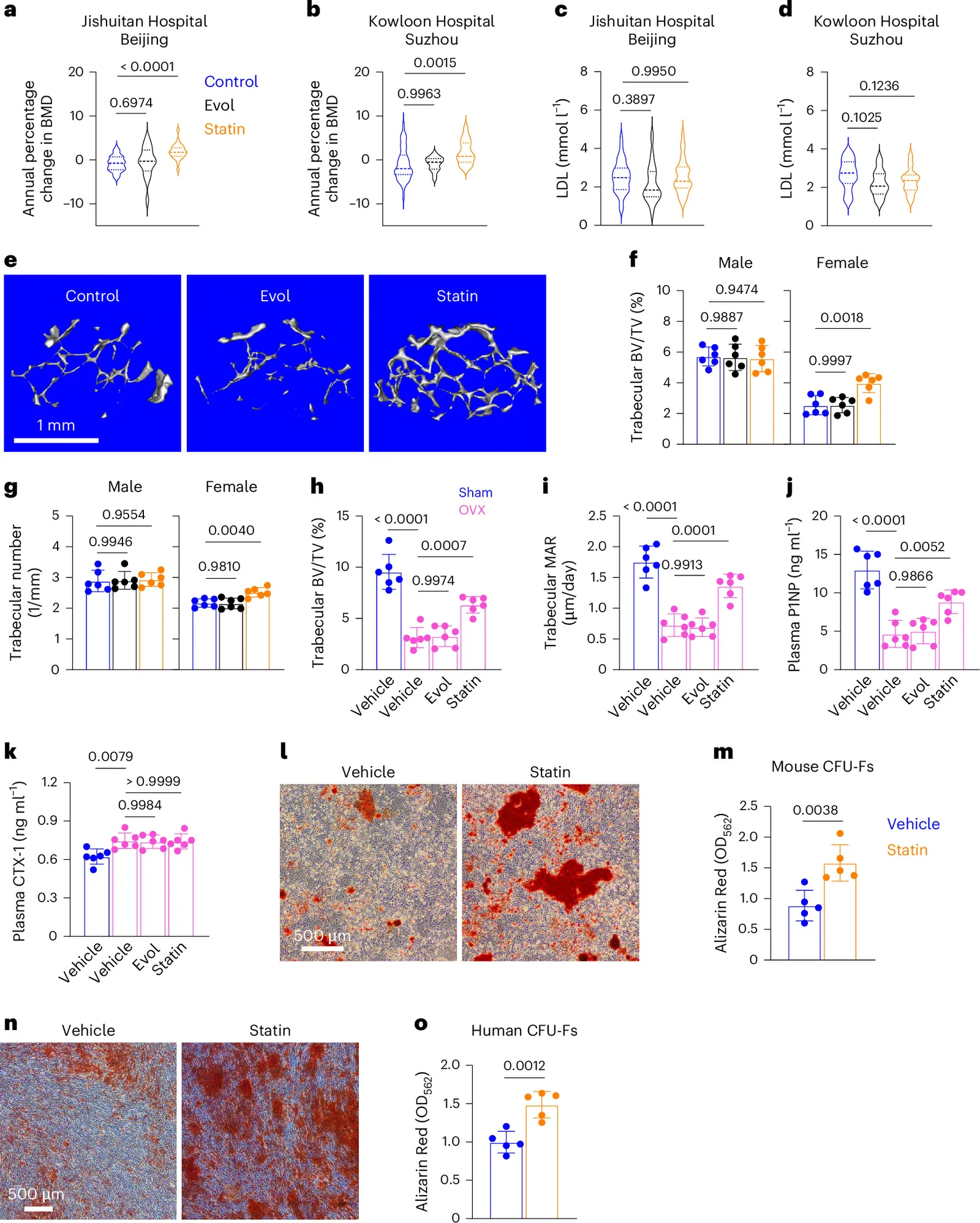
Statins promote bone anabolism in estrogen-deficient women and mice. **a**–**d**, Annual percentage change in lumbar spine BMD in postmenopausal women stratified into statin users, evolocumab users and controls with no lipid-lowering therapy: Beijing Jishuitan Hospital (**a**; control, *n* = 36; evolocumab, *n* = 17; statin, *n* = 27) and Kowloon Hospital (**b**; control, *n* = 49; evolocumab, *n* = 15; statin, *n* = 25). Plasma LDL cholesterol levels were comparable among statin-treated, evolocumab-treated and control groups at either Beijing Jishuitan Hospital (**c**) or Kowloon Hospital (**d**). **e**–**g**, Representative microCT images of trabecular bone in the distal femur metaphysis (**e**) and quantification of trabecular bone volume (BV)/total volume (TV) (**f**) and trabecular number (**g**) in 18-month-old male and female mice treated with vehicle, atorvastatin, or evolocumab (*n* = 6 mice per treatment per sex; three independent experiments). **h**, Quantification of trabecular bone volume in female mice subjected to sham surgery or ovariectomy (OVX) followed by treatment with vehicle, atorvastatin, or evolocumab (*n* = 6 mice per group; three independent experiments). **i**, Trabecular bone MAR measured by calcein and Alizarin Red double labeling in OVX mice treated with vehicle, atorvastatin, or evolocumab (*n* = 6 mice per group; three independent experiments). **j**,**k**, Plasma levels of P1NP (**j**) and CTX-1 (**k**) in OVX mice following treatment with vehicle, atorvastatin, or evolocumab (*n* = 6 mice per group; three independent experiments). **l**–**o**, Atorvastatin promoted osteogenic differentiation of mouse (**l** and **m**) and human (**n** and **o**) CFU-F cultures, shown by representative Alizarin Red staining (**l** and **n**) and quantification (**m** and **o**; mouse: *n* = 5 mice; human: *n* = 5 donors; five independent experiments). Data are presented as the mean ± s.d. Statistical significance was determined by Kruskal–Wallis test followed by Dunn’s multiple-comparisons adjustment (**a**), Welch’s analysis of variance (ANOVA) followed by Dunnett’s T3 multiple-comparisons adjustment (**b** and **d**), one-way ANOVA followed by Tukey’s multiple-comparisons adjustment (**c** and **f**–**k**) or Student’s *t*-test (**m** and **o**). All tests were two sided. Not significant (NS), *P* > 0.05. Source data

**Extended Data Fig. 1:**
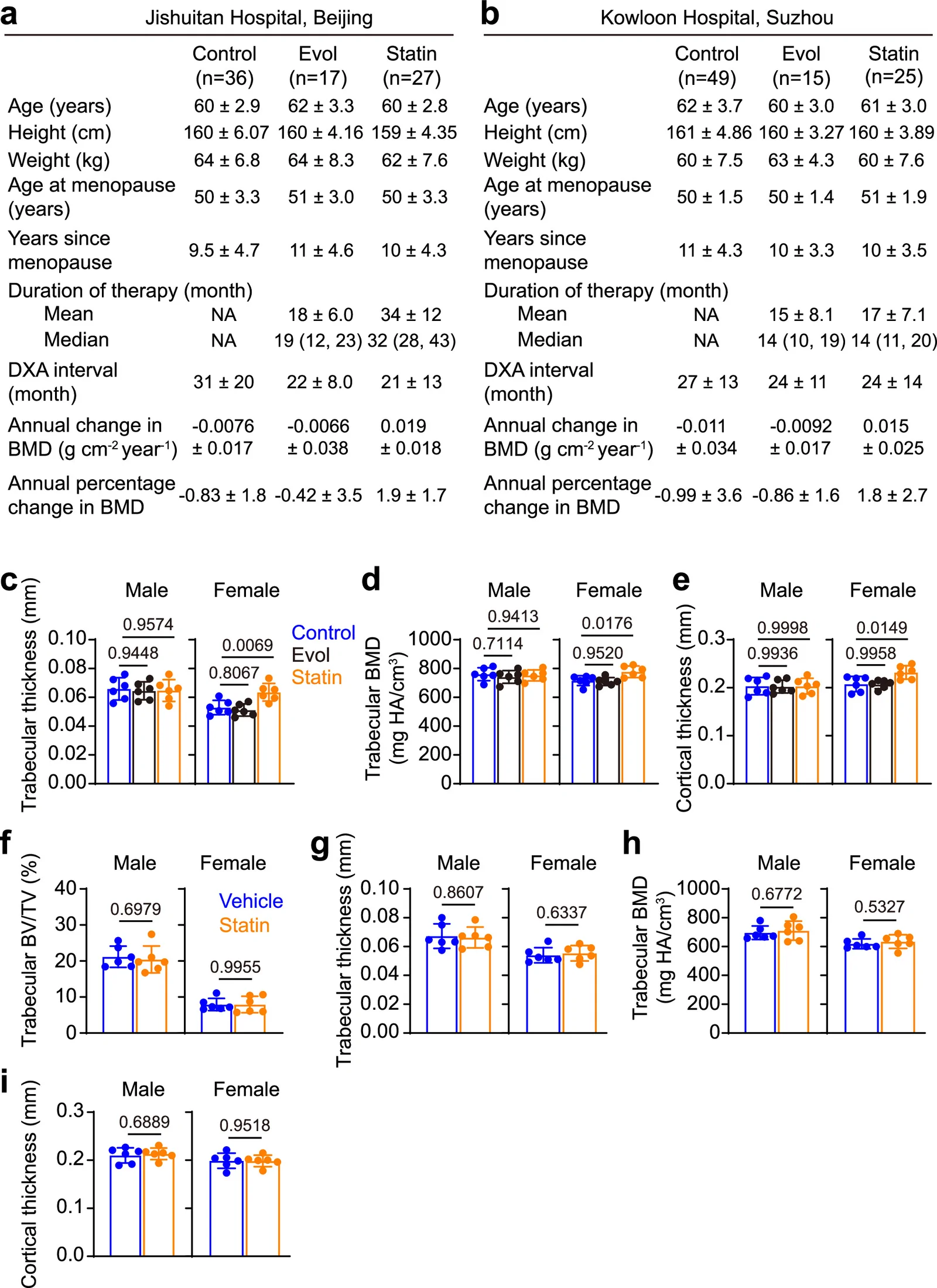
Context-dependent skeletal effects of statins in postmenopausal women and estrogen-deficient mice. (**a**, **b**) Baseline characteristics, menopause-related variables, duration of lipid-lowering therapy, DXA interval, and absolute and percentage annualized lumbar spine BMD changes in participants from Beijing Jishuitan Hospital (a; control, n = 36; evolocumab, n = 17; statin, n = 27) and Kowloon Hospital (b; control, n = 49; evolocumab, n = 15; statin, n = 25). (**c**–**e**) microCT analyses of trabecular thickness (c) and trabecular BMD (d) in the distal femur metaphysis, and cortical thickness (e) in the femoral mid-diaphysis of 18-month-old male and female mice treated with vehicle, atorvastatin, or evolocumab (n = 6 mice per treatment per sex; 3 independent experiments). (**f**–**i**) microCT analyses of trabecular bone volume (**f**), trabecular thickness (**g**), and trabecular BMD (**h**) in the distal femur metaphysis, and cortical thickness (**i**) in the femoral mid-diaphysis of wild-type 2-month-old male and female mice treated with vehicle or atorvastatin (n = 6 mice per treatment per sex; 3 independent experiments). Data are presented as mean ± s.d. Statistical significance was determined by Kruskal–Wallis tests followed by Dunn’s multiple comparisons adjustment, Mann–Whitney test, Student’s t-test, one-way ANOVAs followed by Tukey’s multiple comparisons adjustment, and Welch’s ANOVAs followed by Dunnett’s T3 multiple comparisons adjustment (**a**, **b**), one-way ANOVAs followed by Tukey’s multiple comparisons adjustment (**c**–**e**), or Student’s t-tests (**f**–**i**). All tests were two-sided. NS, *P* > 0.05. Source data

To evaluate the impact of statins on bone in estrogen-deficient women, we performed a retrospective analysis of paired dual-energy X-ray absorptiometry (DXA) data from postmenopausal women aged 55–65 years. In an initial cohort from Beijing Jishuitan Hospital (*n* = 80), participants had been postmenopausal for at least 3 years, with no significant differences in measured baseline variables among groups (Extended Data Fig. 1a). Individuals with metabolic diseases or receiving medications known to affect bone mass were excluded. Statin use was associated with a significantly slower annual decline in BMD compared with nonusers (Fig. 1a). This association was replicated in an independent cohort from Kowloon Hospital in Suzhou (*n* = 89; Fig. 1b and Extended Data Fig. 1b). Together, these two cohorts support an association between statin use and attenuated annual BMD loss in postmenopausal women.

**Extended Data Fig. 2:**
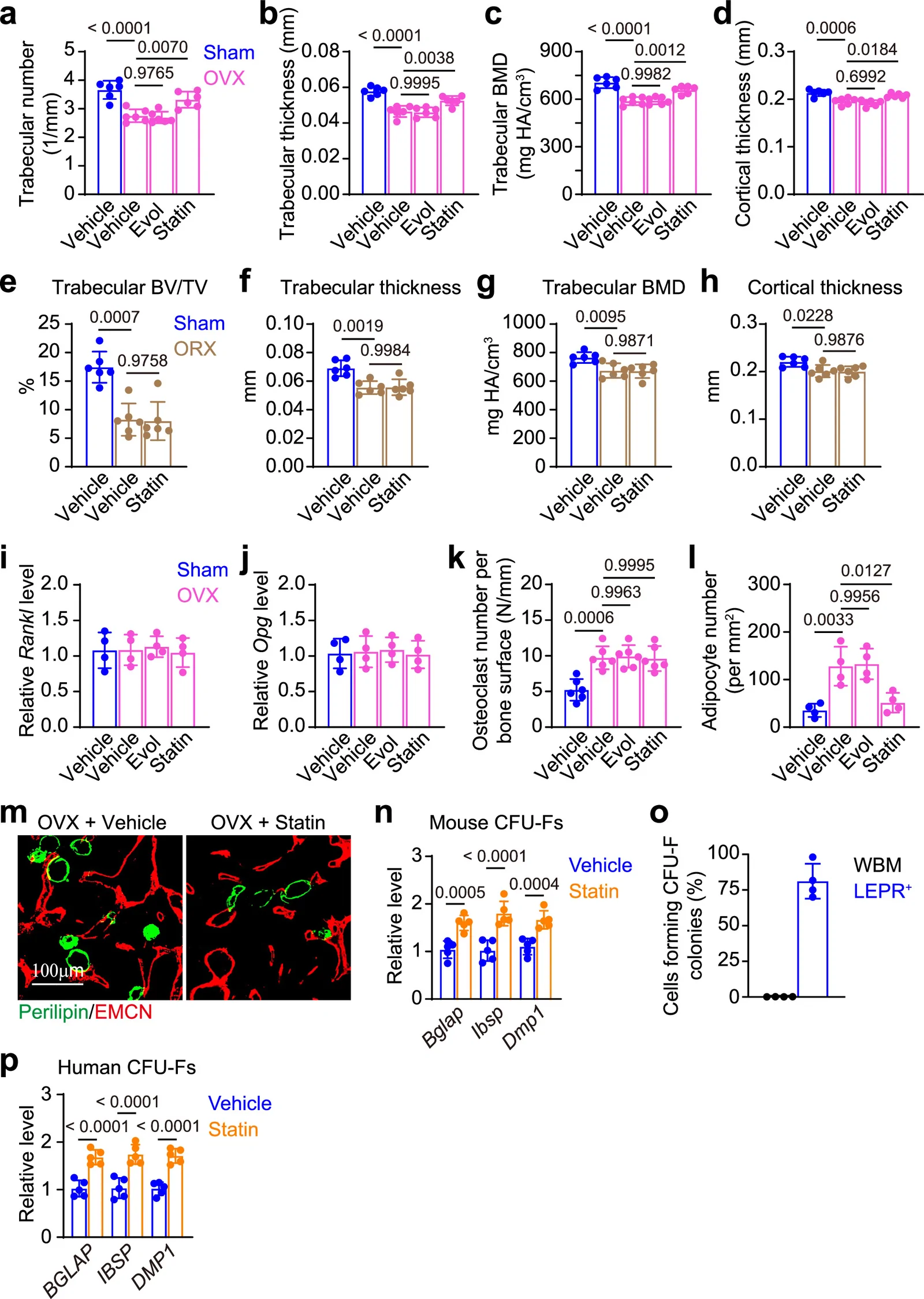
Atorvastatin promotes bone anabolism in ovariectomized but not orchiectomized mice and enhances CFU-F osteogenic differentiation. (**a**–**d**) microCT analyses of trabecular number (**a**), trabecular thickness (**b**), trabecular BMD (**c**) in the distal femur metaphysis, and cortical thickness (**d**) in the femoral mid-diaphysis of wild-type female mice subjected to sham surgery or OVX followed by treatment with vehicle, atorvastatin, or evolocumab (n = 6 mice per group; 3 independent experiments). (**e**–**h**) microCT analyses of trabecular bone volume (**e**), trabecular thickness (**f**), trabecular BMD (**g**) in the distal femur metaphysis, and cortical thickness (**h**) in the femoral mid-diaphysis of wild-type male mice subjected to sham surgery or orchiectomy (ORX) followed by treatment with vehicle, or atorvastatin (n = 6 mice per group; 3 independent experiments). (**i, j**) qPCR analysis of *Rankl* (i) and *Opg* (**j**) transcript levels in sorted LEPR^+^ cells from wild-type female mice subjected to sham surgery or OVX followed by treatment with vehicle, atorvastatin, or evolocumab (n = 4 mice per group; 4 independent experiments). (**k**–**m**) Quantification of osteoclast number on trabecular bone surface from the distal femur metaphysis (**k**), quantification of adipocyte number in metaphyseal bone marrow (**l**), and representative images (**m**) from wild-type female mice subjected to sham surgery or OVX followed by treatment with vehicle, atorvastatin, or evolocumab (n = 6 (k) or 4 (l) mice per group; 3 (**k**) or 4 (**l**) independent experiments). (**n**) qPCR analysis of *Bglap*, *Ibsp*, and *Dmp1* expression in murine CFU-Fs after 42 days of osteogenic differentiation with atorvastatin or vehicle treatment (n = 5 mice; 5 independent experiments). (**o**) Percentage of human LEPR^+^ cells and unfractionated WBM cells that formed CFU-F colonies in culture (n = 4 donors; 4 independent experiments). (**p**) qPCR analysis of *BGLAP*, *IBSP*, and *DMP1* expression in human CFU-Fs after 42 days of osteogenic differentiation with atorvastatin or vehicle treatment (n = 5 donors; 5 independent experiments). Data are presented as mean ± s.d. Statistical significance was determined by one-way ANOVAs followed by Tukey’s multiple comparisons adjustment (**a**–**l**), two-way ANOVAs followed by Šídák’s multiple comparisons adjustment (**n**, **p**), and Welch’s t-test (**o**). All tests were two-sided. NS, *P* > 0.05. Source data

Statins lower LDL cholesterol^20^, raising the possibility that LDL reduction could contribute to the observed differences in BMD. LDL levels were comparable among statin, evolocumab and control groups in both cohorts (Fig. 1c,d), but evolocumab was not associated with a slower annual decline in BMD (Fig. 1a,b). In 18-month-old female mice, atorvastatin, but not evolocumab, significantly increased trabecular bone volume, number, thickness and BMD, as well as cortical thickness, as revealed by micro-computed tomography (microCT; Fig. 1e–g and Extended Data Fig. 1c–e). Atorvastatin did not measurably increase bone mass in young adult mice (Extended Data Fig. 1f–i), but increased bone mass after ovariectomy, whereas evolocumab did not (Fig. 1h and Extended Data Fig. 2a–d). Atorvastatin also did not increase bone mass after orchiectomy (ORX; Extended Data Fig. 2e–h). These results thus establish an LDL-independent bone anabolic effect of statins in estrogen-deficient mice, consistent with the association between statin use and attenuated annual BMD loss in postmenopausal women.

### Statins promote osteogenic differentiation of LEPR^+^ cells

Bone mass is maintained through a balance of bone formation and resorption^18^. We therefore assessed whether statins increase bone formation (that is, bone anabolism) and/or reduce bone resorption. In ovariectomized mice, atorvastatin increased mineral apposition rate (MAR) and plasma levels of procollagen type I N-terminal propeptide (P1NP; Fig. 1i,j). By contrast, plasma C-terminal telopeptide of type I collagen (CTX-1), *Rankl/Tnfsf11* and *Opg/Tnfrsf11b* transcription in LEPR^+^ cells, and osteoclast number on trabecular bone surfaces were unchanged (Fig. 1k and Extended Data Fig. 2i–k). Thus, the statin-induced increase in bone mass reflects enhanced bone formation rather than reduced bone resorption.

More than 90% of osteoblasts and osteocytes formed during adulthood are derived from LEPR^+^ cells^13,14^, raising the possibility that statin-induced bone anabolism results from enhanced osteogenic differentiation of this population. Atorvastatin also reduced bone marrow adipocyte number in ovariectomized mice (Extended Data Fig. 2l,m). In mouse colony-forming unit-fibroblasts (CFU-Fs), which arise from LEPR^+^ cells^14^, atorvastatin promoted osteogenic differentiation and increased expression of *Bglap*, *Ibsp* and *Dmp1* (Fig. 1l,m and Extended Data Fig. 2n). Consistent with the mouse data, CFU-F activity in human bone marrow was detected predominantly in the LEPR^+^ fraction, and atorvastatin likewise promoted osteogenic differentiation of human CFU-Fs (Fig. 1n,o and Extended Data Fig. 2o,p). These findings raise the possibility that statins promote bone anabolism by enhancing the osteogenic differentiation of LEPR^+^ cells.

**Extended Data Fig. 3:**
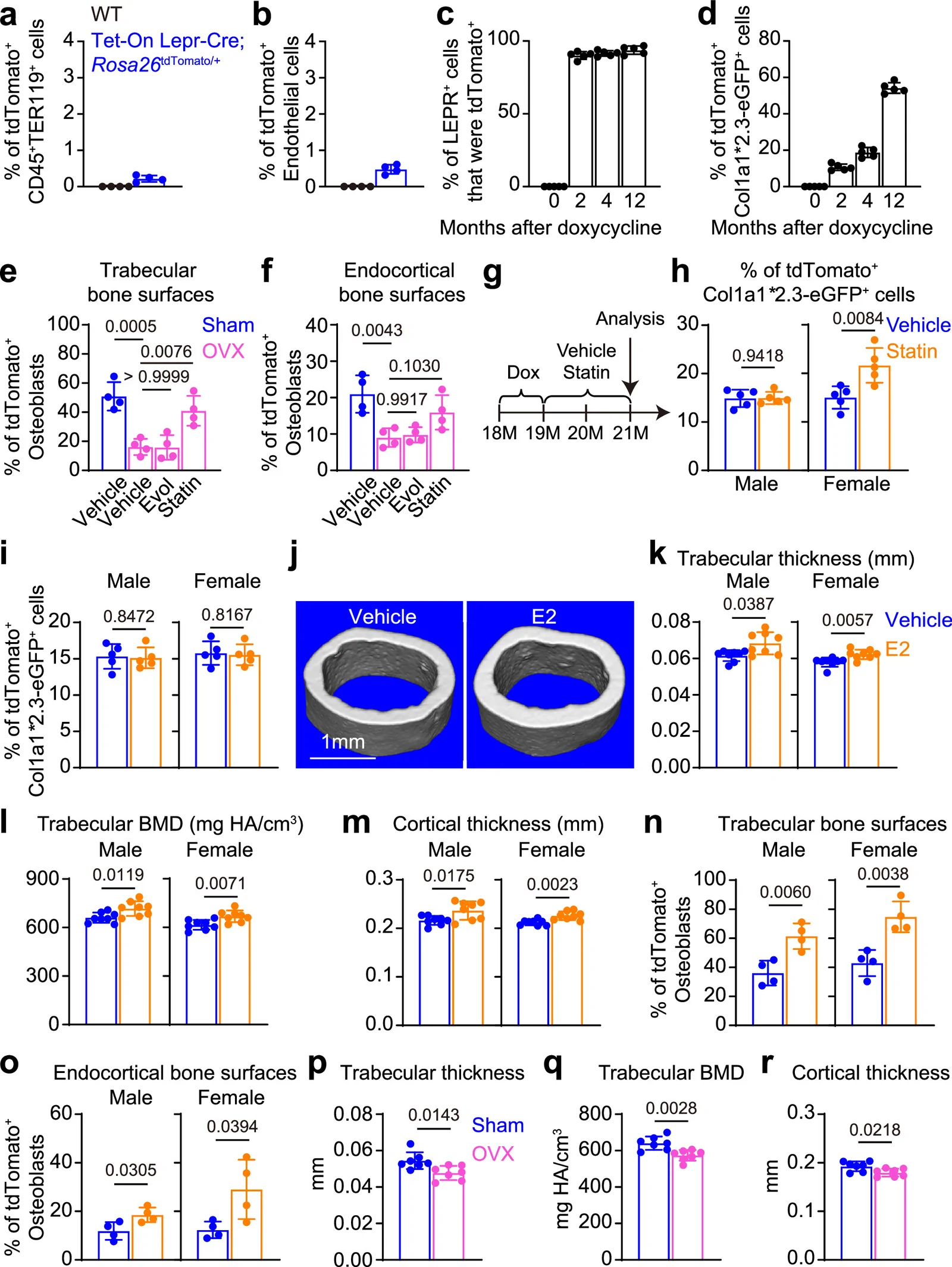
Tet-On Lepr-Cre fate mapping shows that osteogenic differentiation of LEPR^+^ cells is enhanced by atorvastatin under estrogen-deficient conditions and by E2 treatment, but reduced after ovariectomy. (**a**, **b**) Quantification of the percentage of hematopoietic cells (**a**) and endothelial cells (**b**) in bone marrow that were tdTomato^+^ from 2-month-old Tet-On Lepr-Cre; *Rosa26*^tdTomato/+^ mice after doxycycline induction (n = 4 mice per group; 4 independent experiments). (**c**) Quantification of the percentage of LEPR^+^ cells that were tdTomato^+^ in bone marrow from Tet-On Lepr-Cre; *Rosa26*^tdTomato/+^; Col1a1*2.3-eGFP mice before doxycycline induction at 2 months of age and at 2, 4, and 12 months after doxycycline induction (n = 5 mice per time point; 5 independent experiments). (**d**) Quantification of the percentage of Col1a1*2.3-eGFP^+^ osteoblasts that were tdTomato^+^ in Tet-On Lepr-Cre; *Rosa26*^tdTomato/+^; Col1a1*2.3-eGFP mice before doxycycline induction at 2 months of age and at 2, 4, and 12 months after doxycycline induction (n = 5 mice per time point; 5 independent experiments). (**e**, **f**) Quantification of the percentage of Col1a1*2.3-eGFP^+^ osteoblasts that were tdTomato^+^ at trabecular (**e**) and endocortical (**f**) bone surfaces in Tet-On Lepr-Cre; *Rosa26*^tdTomato/+^; Col1a1*2.3-eGFP female mice subjected to sham or OVX surgery followed by treatment with vehicle, atorvastatin, or evolocumab (n = 4 mice per group; 4 independent experiments). (**g**, **h**) Schematic overview of the experimental design (**g**). Atorvastatin increased the contribution of LEPR^+^ cells to osteoblasts in aged female Tet-On Lepr-Cre; *Rosa26*^tdTomato/+^; Col1a1*2.3-eGFP mice but not in aged male mice. Contribution was quantified as the fraction of Col1a1*2.3-eGFP^+^ cells that were tdTomato^+^, as measured by flow cytometry (**h**) (n = 5 mice per group per sex; 5 independent experiments). (**i**) Quantification of the percentage of Col1a1*2.3-eGFP^+^ osteoblasts that were tdTomato^+^ in 2-month-old Tet-On Lepr-Cre; *Rosa26*^tdTomato/+^; Col1a1*2.3-eGFP mice administered doxycycline for 4 weeks beginning at 2 months of age and subsequently treated with vehicle or atorvastatin for 8 weeks (n = 5 mice per group per sex; 5 independent experiments). (**j**–**m**) Representative cortical bone microCT images (**j**) and quantitative microCT analyses of trabecular thickness (**k**), trabecular BMD (**l**) in the distal femur metaphysis, and cortical thickness (**m**) in the femoral mid-diaphysis in wild-type mice treated with vehicle or E2 (n = 8 mice per group per sex; 4 independent experiments). (**n, o**) Quantification of the percentage of Col1a1*2.3-eGFP^+^ osteoblasts that were tdTomato^+^ at trabecular (n) and endocortical (o) bone surfaces in Tet-On Lepr-Cre; *Rosa26*^tdTomato/+^; Col1a1*2.3-eGFP mice treated with vehicle or E2 (n = 4 mice per group; 4 independent experiments). (**p**–**r**) microCT analyses of trabecular thickness (**p**), trabecular BMD (**q**) in the distal femur metaphysis, and cortical thickness (**r**) in the femoral mid-diaphysis of female mice subjected to sham or OVX surgery (n = 7 mice per group; 4 independent experiments). Data are presented as mean ± s.d. Statistical significance was determined by one-way ANOVAs followed by Tukey’s multiple comparisons adjustment (**e** and **f**), Student’s t-tests (**h**, **i**, **l**–**r**), and Mann-Whitney tests (**k**). All tests were two-sided. NS, *P* > 0.05. Source data

To test this directly, we generated a doxycycline-inducible Tet-On *Lepr*-Cre allele to examine the osteogenic fate of LEPR^+^ cells in vivo while avoiding potential confounding effects of tamoxifen, an estrogen receptor modulator with well-documented bone anabolic activity^21^. Doxycycline administration at 2 months of age in Tet-On *Lepr*-Cre; *Rosa26*^tdTomato/+^; Col1a1*2.3-eGFP mice efficiently and specifically labeled LEPR^+^ cells (Extended Data Fig. 3a–c), enabling fate mapping of LEPR^+^ cells to Col1a1*2.3-eGFP^+^ osteoblasts in vivo. Longitudinal fate mapping further showed that Tet-On *Lepr*-Cre-labeled LEPR^+^ cells persisted during adulthood and progressively contributed to osteoblast formation over time (Extended Data Fig. 3c,d), as previously observed using *Lepr*-CreER^13^.

**Fig. 2:**
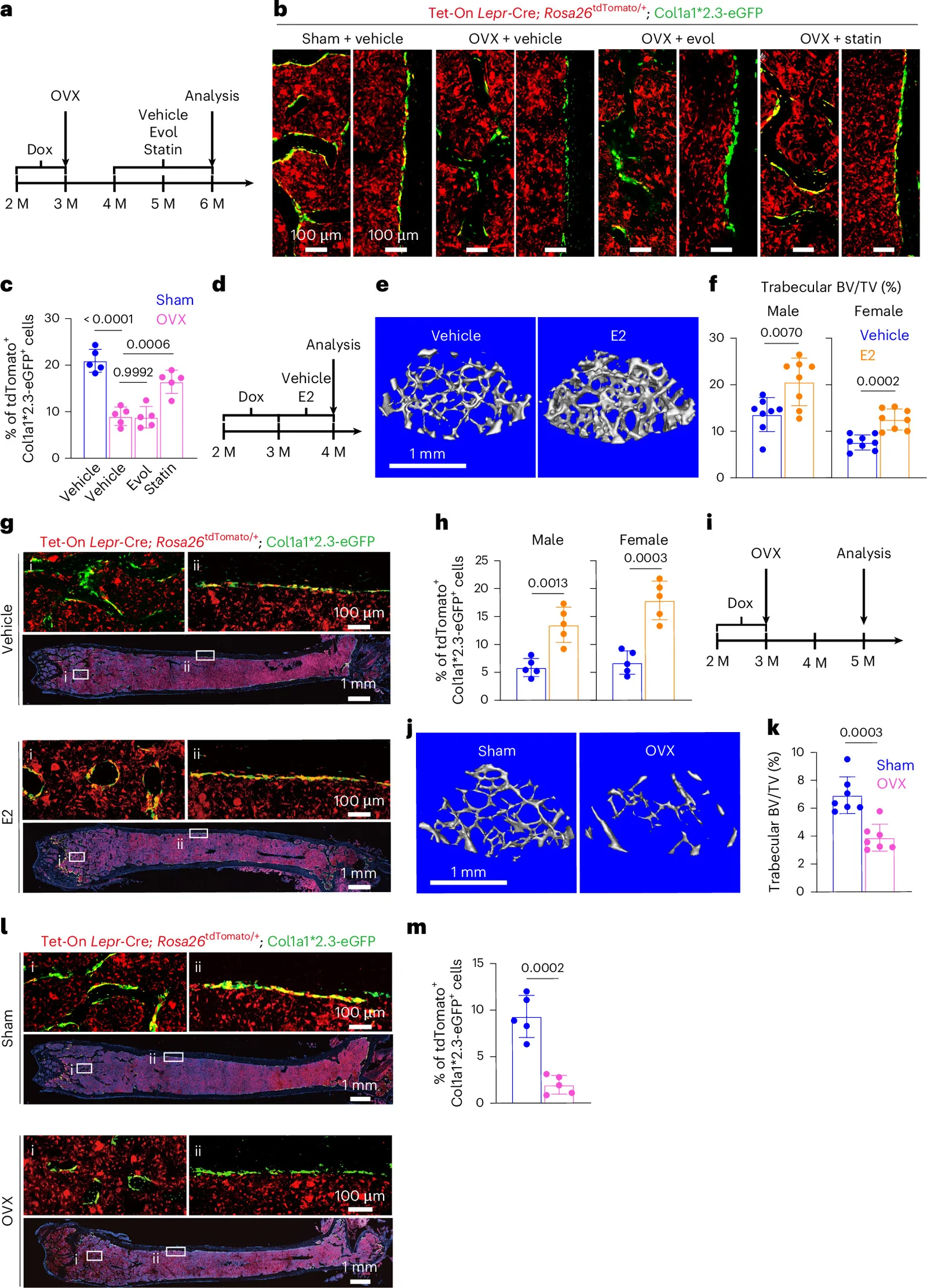
Statins and estrogen promote osteogenic differentiation of LEPR^+^ cells. **a**–**c**, In ovariectomized Tet-On *Lepr*-Cre; *Rosa26*^tdTomato/+^; Col1a1*2.3-eGFP mice, atorvastatin increased the contribution of LEPR^+^ cells to osteoblasts and osteocytes, reflected by an increased fraction of Col1a1*2.3-eGFP^+^ cells that were tdTomato^+^; schematic overview of the experimental design (**a**), representative femoral immunofluorescence images (**b**) and quantification by flow cytometry (**c**; *n* = 5 mice per group; five independent experiments). **d**–**f**, Schematic overview of the experimental design (**d**), microCT images (**e**) and quantification of trabecular bone volume (**f**) in the distal femur metaphysis of mice treated with vehicle or E2 (*n* = 8 mice per treatment per sex; four independent experiments). **g**,**h**, E2 increased the fraction of osteoblasts and osteocytes derived from LEPR^+^ cells, measured as the percentage of Col1a1*2.3-eGFP^+^ cells that were tdTomato^+^, as determined by immunofluorescence (**g**) and flow cytometry (**h**) in Tet-On *Lepr*-Cre; *Rosa26*^tdTomato/+^; Col1a1*2.3-eGFP mice (*n* = 5 mice per treatment per sex; five independent experiments). **i**–**k**, Schematic overview of the experimental design (**i**), representative microCT images (**j**) and quantification of trabecular bone volume (**k**) in the distal femur metaphysis of female mice subjected to sham or OVX surgery (*n* = 7 mice per group; four independent experiments). **l**,**m**, OVX reduced the contribution of LEPR^+^ cells to osteoblasts and osteocytes, shown by a decreased percentage of Col1a1*2.3-eGFP^+^ cells that were tdTomato^+^, as determined by immunofluorescence (**l**) and flow cytometry (**m**) in female Tet-On *Lepr*-Cre; *Rosa26*^tdTomato/+^; Col1a1*2.3-eGFP mice (*n* = 5 mice per group; five independent experiments). Data are presented as the mean ± s.d. Statistical significance was determined by one-way ANOVA followed by Tukey’s multiple-comparisons adjustment (**c**) or Student’s *t*-test (**f**, **h**, **k** and **m**). All tests were two sided. NS, *P* > 0.05. Dox, doxycycline. Source data

Consistent with our in vitro findings, atorvastatin promoted osteogenic differentiation of LEPR^+^ cells into osteoblasts in ovariectomized mice (Fig. 2a–c and Extended Data Fig. 3e,f), with a more pronounced effect at trabecular than endocortical bone surfaces (Extended Data Fig. 3e,f). A similar increase was observed in aged females but not aged males (Extended Data Fig. 3g,h), whereas no increase was detected in young adult mice (Extended Data Fig. 3i). These data demonstrate that statins promote osteogenic differentiation of LEPR^+^ cells under estrogen-deficient conditions, consistent with a bone anabolic effect in vivo.

### Estrogen per se promotes osteogenic differentiation of LEPR^+^ cells

Having established that statins promote osteogenic differentiation of LEPR^+^ cells in vivo, we next asked whether estrogen itself acts on the same population. Previous studies have implicated osteogenic progenitors, osteoblasts and osteocytes in estrogen-mediated bone formation^16,17,22^, but whether estrogen acts directly on LEPR^+^ cells remains unknown. We therefore administered doxycycline and implanted E2 pumps into 2-month-old male and female Tet-On *Lepr*-Cre; *Rosa26*^tdTomato/+^; Col1a1*2.3-eGFP mice, delivering E2 at 10 μg per kg per day for 4 weeks. MicroCT showed that E2 significantly increased trabecular bone volume, trabecular thickness, trabecular BMD and cortical thickness in both sexes (Fig. 2d–f and Extended Data Fig. 3j–m). Flow cytometric and immunofluorescence analyses further showed that E2 significantly increased the contribution of LEPR^+^ cells to osteoblast formation (Fig. 2g,h and Extended Data Fig. 3n,o).

Estrogen deficiency reduces bone formation partly by impairing osteoblast formation, including effects on osteogenic progenitors and osteoblasts^16,22^, but whether it affects earlier skeletal stem/progenitor stages remains unclear. In ovariectomized mice, microCT analysis revealed significant reductions in trabecular bone volume, trabecular thickness, trabecular BMD and cortical thickness compared with sham-operated controls (Fig. 2i–k and Extended Data Fig. 3p–r). Fate-mapping analyses further demonstrated reduced osteoblast and osteocyte formation from LEPR^+^ cells (Fig. 2l,m). Collectively, these results establish that estrogen per se promotes osteogenic differentiation of LEPR^+^ cells.

### Identification of an estrogen-sensitive LEPR^+^ cell population at early stages of osteogenic differentiation

**Extended Data Fig. 4:**
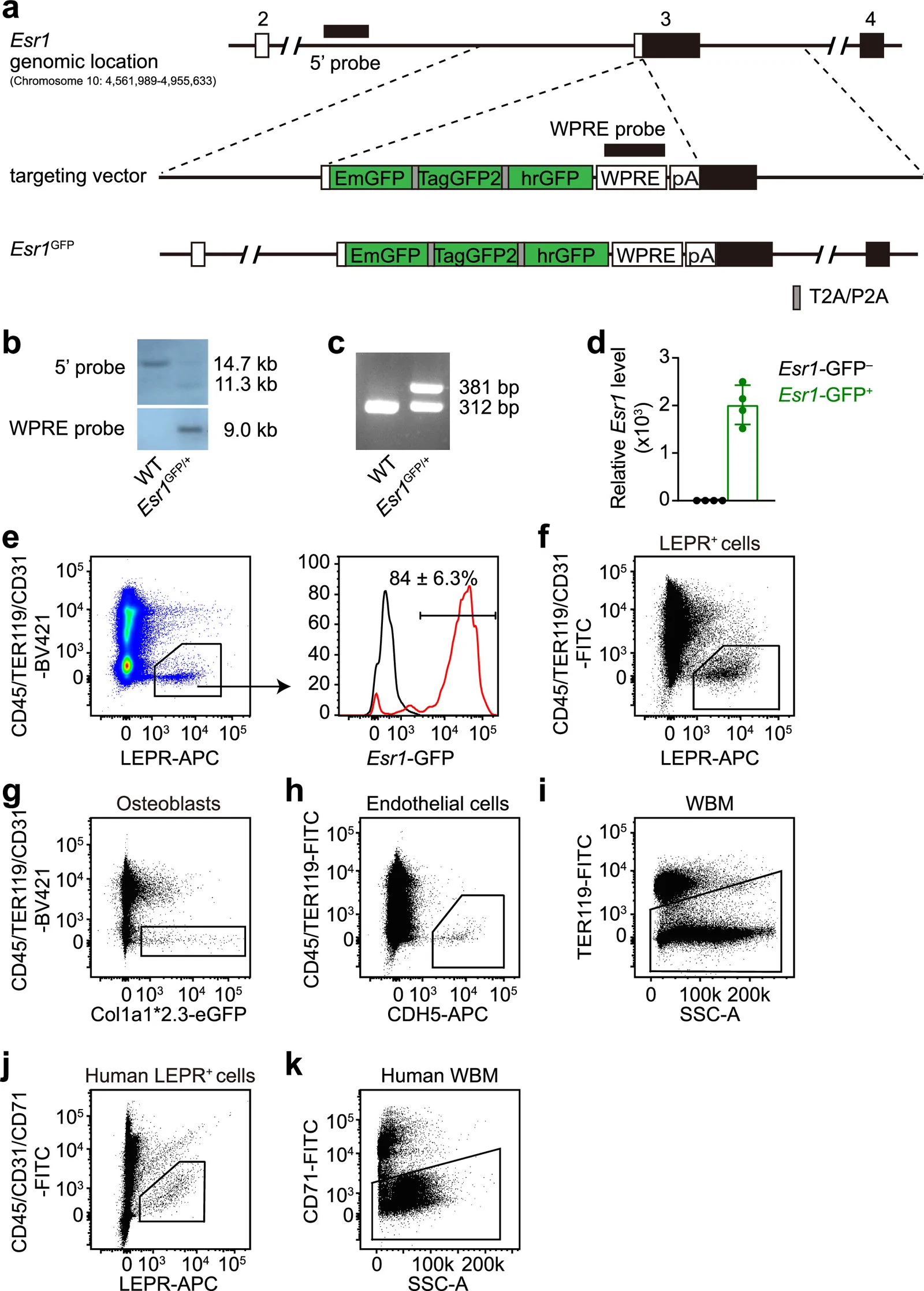
Generation and validation of the *Esr1*^GFP^ reporter allele. (**a**) Targeting strategy for generation of the *Esr1*^GFP^ mouse allele, in which an *EmGFP–T2A–TagGFP2–P2A–hrGFP–WPRE–polyA* cassette (adapted from the Ai47 allele^60^) was inserted downstream of an alternative ATG in exon 3 of the *Esr1* locus. Untranslated regions are shown as open boxes and coding regions as filled boxes. (**b**) Southern blotting using 5’ and WPRE probes (black bars in a) identifying the correctly targeted F0 founder (representative of three independent experiments). (**c**) PCR-based genotyping confirming germline transmission of the *Esr1*^GFP^ allele (representative of three independent experiments). (**d**) Reverse transcription–qPCR quantification of *Esr1* transcripts in sorted GFP^+^ versus GFP^−^ bone marrow cells, showing that *Esr1* transcripts were detected in GFP^+^ cells but not in GFP^−^ cells (n = 4 mice; 4 independent experiments). (**e**) Quantification of the percentage of LEPR^+^ cells that expressed *Esr1*-GFP in adult *Esr1*^GFP/+^ mice (n = 4 mice; 4 independent experiments). (**f**–**i**) Representative flow cytometry gating strategies for identification of LEPR^+^ cells (f), Col1a1*2.3-eGFP^+^CD45^−^TER119^−^CD31^−^ osteoblasts (**g**), VE-cadherin^+^CD45^−^TER119^−^ endothelial cells (**h**), and TER119^−^ whole bone marrow (WBM) cells (**i**) in mice. (**j**, **k**) Representative flow cytometry gating strategies for identification of LEPR^+^ cells (**j**) and CD71^−^ WBM cells (**k**) from human bone marrow samples. Data are presented as mean ± s.d. Source data

Because LEPR^+^ cells contain skeletal stem/progenitor cells that form osteoblasts and osteocytes^13–15^, our findings suggested that E2 may act earlier in osteogenic differentiation than previously assumed. To identify ERα-expressing cells along this trajectory, we generated an *Esr1*^GFP^ knock-in reporter allele (Extended Data Fig. 4a–c). *Esr1* transcripts were highly enriched in *Esr1*-GFP^+^ cells but undetectable in *Esr1*-GFP^−^ cells (Extended Data Fig. 4d), indicating that GFP faithfully reports endogenous *Esr1* expression.

**Fig. 3:**
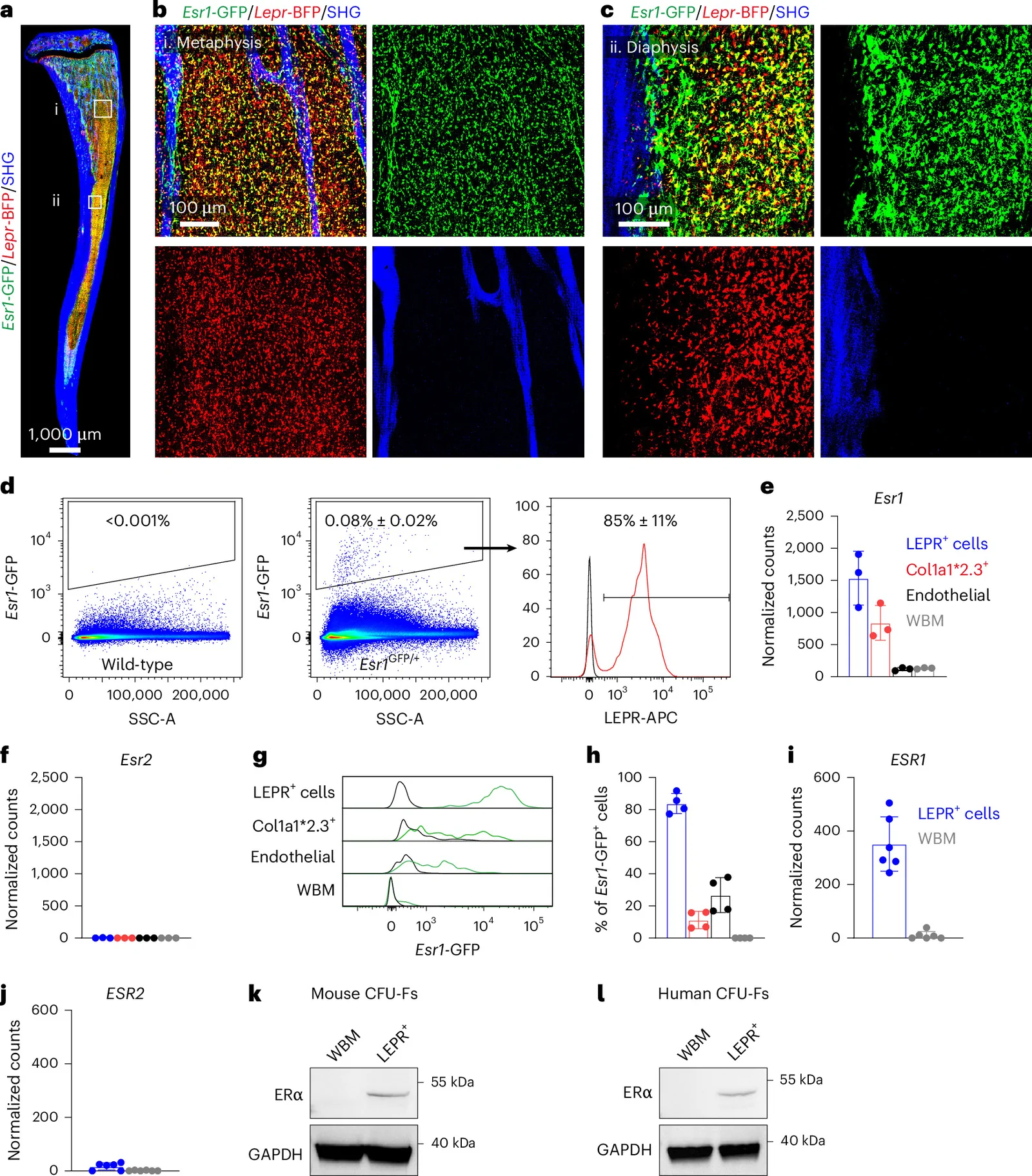
Estrogen-sensitive LEPR^+^ cells express ERα abundantly at early stages of osteogenic differentiation. **a**–**c**, Deep confocal imaging of tibia hemisections from 2-month-old *Esr1*^GFP/+^; *Lepr*^BFP/+^ mice revealed extensive overlap of *Esr1*-GFP^+^ cells with *Lepr*-BFP^+^ cells throughout the bone marrow, in both the metaphysis (**b**) and diaphysis (**c**) (representative images from three mice). **d**, Flow cytometric analysis of enzymatically dissociated bone marrow from *Esr1*^GFP/+^ mice showed that 85% ± 11% of *Esr1*-GFP^+^ cells were LEPR^+^ (*n* = 4 mice; four independent experiments). SSC-A, side scatter area. **e**,**f**, RNA-seq analysis of *Esr1* (**e**) and *Esr2* (**f**) expression in sorted bone marrow populations from 2-month-old mice, including LEPR^+^CD45^−^TER119^−^CD31^−^ stromal cells (LEPR^+^), Col1a1*2.3-eGFP^+^CD45^−^TER119^−^CD31^−^ osteoblasts, VE-cadherin^+^CD45^−^TER119^−^ endothelial cells and unfractionated whole bone marrow (WBM) cells (*n* = 3 mice per population; three independent experiments). **g**, Representative flow cytometric histograms showing *Esr1*-GFP fluorescence intensity in indicated bone marrow cell populations from *Esr1*^GFP/+^; *Col1a1*-CreER; *Rosa26*^tdTomato/+^ mice, in which tamoxifen was administered at 2 months of age to label osteoblasts. **h**, Quantification of the frequency of *Esr1*-GFP^+^ cells within LEPR^+^ cells, osteoblasts, endothelial cells and WBM cells following tamoxifen induction in *Esr1*^GFP/+^; *Col1a1*-CreER; *Rosa26*^tdTomato/+^ mice (*n* = 4 mice; four independent experiments). **i**,**j**, RNA-seq analysis of *ESR1* (**i**) and *ESR2* (**j**) expression in sorted human bone marrow LEPR^+^CD45^−^CD31^−^CD71^−^ cells and matched WBM cells (*n* = 6 human donors per population). **k**,**l**, Immunoblots showing ERα protein expression in mouse (**k**) and human (**l**) CFU-Fs, with no detectable ERα in matched WBM controls (representative blots from three independent mice or human donors). Data are presented as the mean ± s.d. Source data

Deep confocal imaging of optically cleared tibiae from 2-month-old *Esr1*^GFP/+^; *Lepr*^BFP/+^ mice revealed extensive overlap between *Esr1*-GFP and *Lepr*-BFP fluorescence (Fig. 3a–c). Most *Lepr*-BFP^+^ cells expressed *Esr1*-GFP, and double-positive cells were broadly distributed near sinusoids and arterioles throughout metaphyseal and diaphyseal marrow (Fig. 3a–c). Flow cytometry showed that *Esr1*-GFP^+^ cells constituted 0.08% ± 0.02% of enzymatically dissociated bone marrow cells. Of these, 85% ± 11% expressed LEPR, while 84% ± 6.3% of LEPR^+^ cells expressed *Esr1*-GFP (Fig. 3d and Extended Data Fig. 4e), indicating extensive overlap between the populations. These data extend the inferred site of estrogen action to a developmentally earlier LEPR^+^ population.

Because estrogen receptor signaling has been implicated in endothelial cells^23^ and osteoblasts and osteocytes^16,24,25^, we compared receptor expression across relevant bone marrow populations by bulk RNA sequencing (RNA-seq). Sorted populations from 2-month-old mice included LEPR^+^ cells (LEPR^+^CD45^−^TER119^−^CD31^−^), osteoblasts (Col1a1*2.3-eGFP^+^CD45^−^TER119^−^CD31^−^), endothelial cells (VE-cadherin^+^CD45^−^TER119^−^) and unfractionated whole bone marrow nucleated cells (TER119^−^; Extended Data Fig. 4f–i). *Esr1* expression was substantially higher in LEPR^+^ cells than in other populations, whereas *Esr2* was undetectable (Fig. 3e,f). *Esr1*-GFP expression was likewise highest in intensity and prevalence among LEPR^+^ cells (Fig. 3g,h). Similarly, *ESR1*, but not *ESR2*, was detected in sorted human LEPR^+^ cells (Fig. 3i,j and Extended Data Fig. 4j,k), and immunoblotting confirmed ERα protein in mouse and human CFU-Fs but not in unfractionated whole bone marrow cells (Fig. 3k,l). Collectively, these results identify LEPR^+^ cells as a major estrogen-responsive population in adult bone marrow, supporting a previously unappreciated role for estrogen signaling during early stages of osteogenic differentiation.

### E2–ERα signaling mediates a major component of E2-induced bone anabolism

**Fig. 4:**
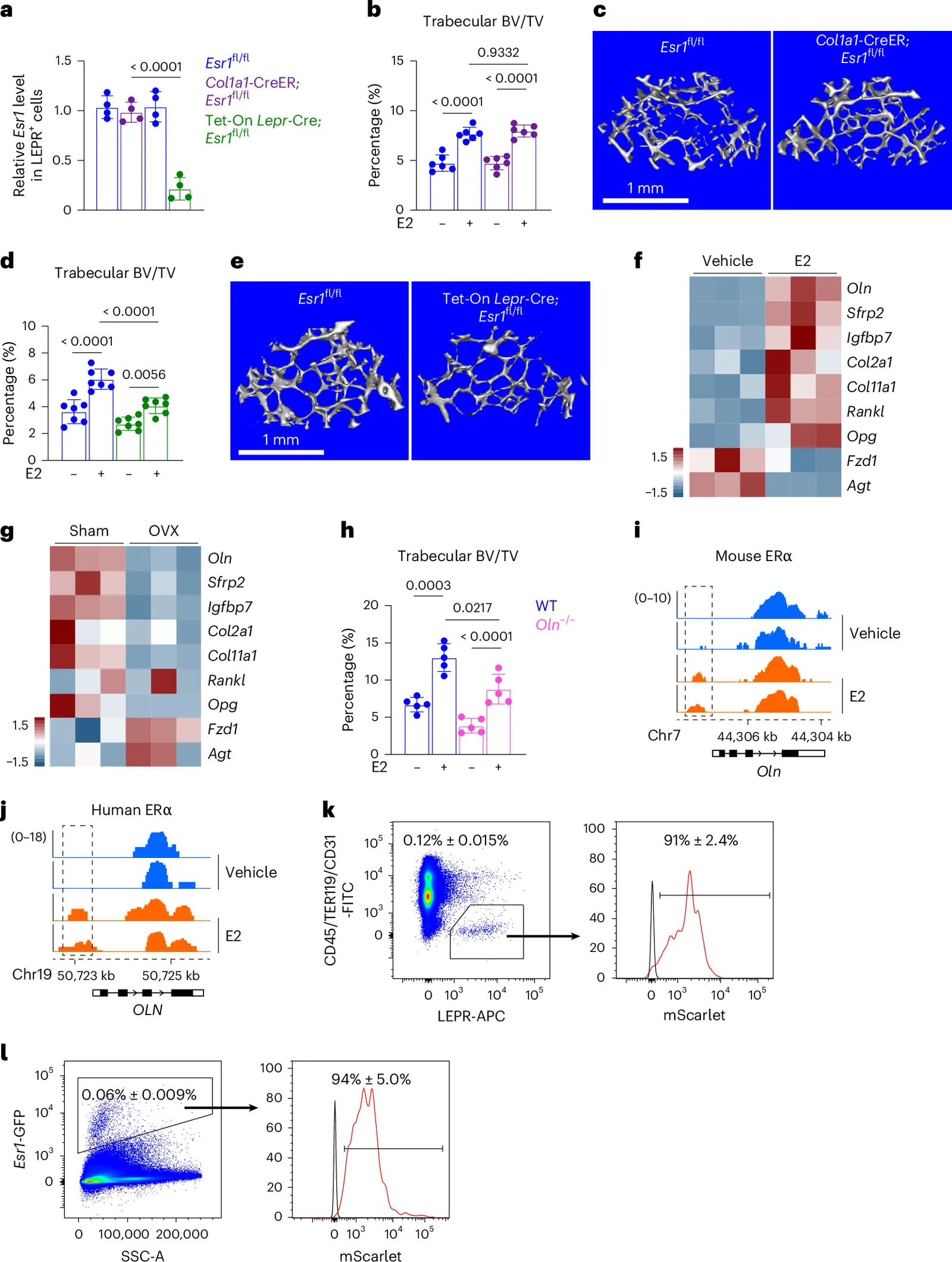
E2–ERα signaling promotes *Oln* transcription in LEPR^+^ cells. **a**, qPCR analysis of *Esr1* transcript levels in sorted LEPR^+^ cells from *Col1a1*-CreER; *Esr1*^fl/fl^ mice (tamoxifen-induced) or Tet-On *Lepr*-Cre; *Esr1*^fl/fl^ mice (doxycycline-induced), with corresponding *Esr1*^fl/fl^ littermates as controls (*n* = 4 mice per genotype; four independent experiments). **b**,**c**, Quantification of trabecular bone volume (**b**) and representative microCT images (**c**) in the distal femur metaphysis of *Col1a1*-CreER; *Esr1*^fl/fl^ mice and littermate controls subjected to OVX at 2 months of age and treated for 4 weeks with E2 or vehicle (*n* = 6 mice per genotype per treatment; three independent experiments). **d**,**e**, Quantification of trabecular bone volume (**d**) and representative microCT images (**e**) in Tet-On *Lepr*-Cre; *Esr1*^fl/fl^ mice and littermate controls subjected to OVX at 2 months of age and treated with E2 or vehicle for 4 weeks (*n* = 7 mice per genotype per treatment; four independent experiments). **f**,**g**, E2 treatment increased (**f**), whereas OVX reduced (**g**), expression of osteogenic genes—including *Oln*—in sorted LEPR^+^ cells under estrogen-replete and estrogen-deficient conditions, respectively (*n* = 3 mice per treatment; three independent experiments). **h**, microCT analysis of trabecular bone volume in female *Oln*^−/−^ mice and littermate controls subjected to OVX at 2 months of age and treated with E2 or vehicle for 4 weeks (*n* = 5 mice per genotype per treatment; three independent experiments). **i**,**j**, ERα CUT&Tag analysis at the mouse *Oln* locus (**i**) and human *OLN* locus (**j**) in CFU-Fs cultured under steroid-depleted conditions and treated with vehicle or E2 (two independent experiments). **k**,**l**, Flow cytometric analysis of enzymatically dissociated bone marrow from *Hipp11*^Oln−URE−mScarlet^; *Esr1*^GFP/+^ mice showed that mScarlet was detected in 91% ± 2.4% of LEPR^+^ cells (**k**) and that 94% ± 5.0% of *Esr1*-GFP^+^ cells were mScarlet^+^ (**l**) (*n* = 4 mice; four independent experiments). Data are presented as the mean ± s.d. Statistical significance was determined by one-way ANOVA followed by Tukey’s multiple-comparisons adjustment (**a**), or two-way ANOVA followed by Šídák’s multiple-comparisons adjustment (**b**, **d** and **h**). All tests were two sided. WT, wild type. NS, *P* > 0.05. Source data

Estrogen-based hormone replacement therapy reduces postmenopausal bone loss partly by promoting bone formation^2,3^. Although previous studies attributed this effect to osteoblasts and osteocytes^16,17^, the *Osx*-Cre and *Dmp1*-Cre lines used to delete *Esr1* also recombine in LEPR^+^ cells^26,27^, precluding unambiguous assignment of the cellular site of action. We therefore used *Col1a1*-CreER^28^ for osteoblast/osteocyte-restricted *Esr1* deletion and Tet-On *Lepr*-Cre for LEPR^+^ cell-specific deletion (Fig. 4a).

**Extended Data Fig. 5:**
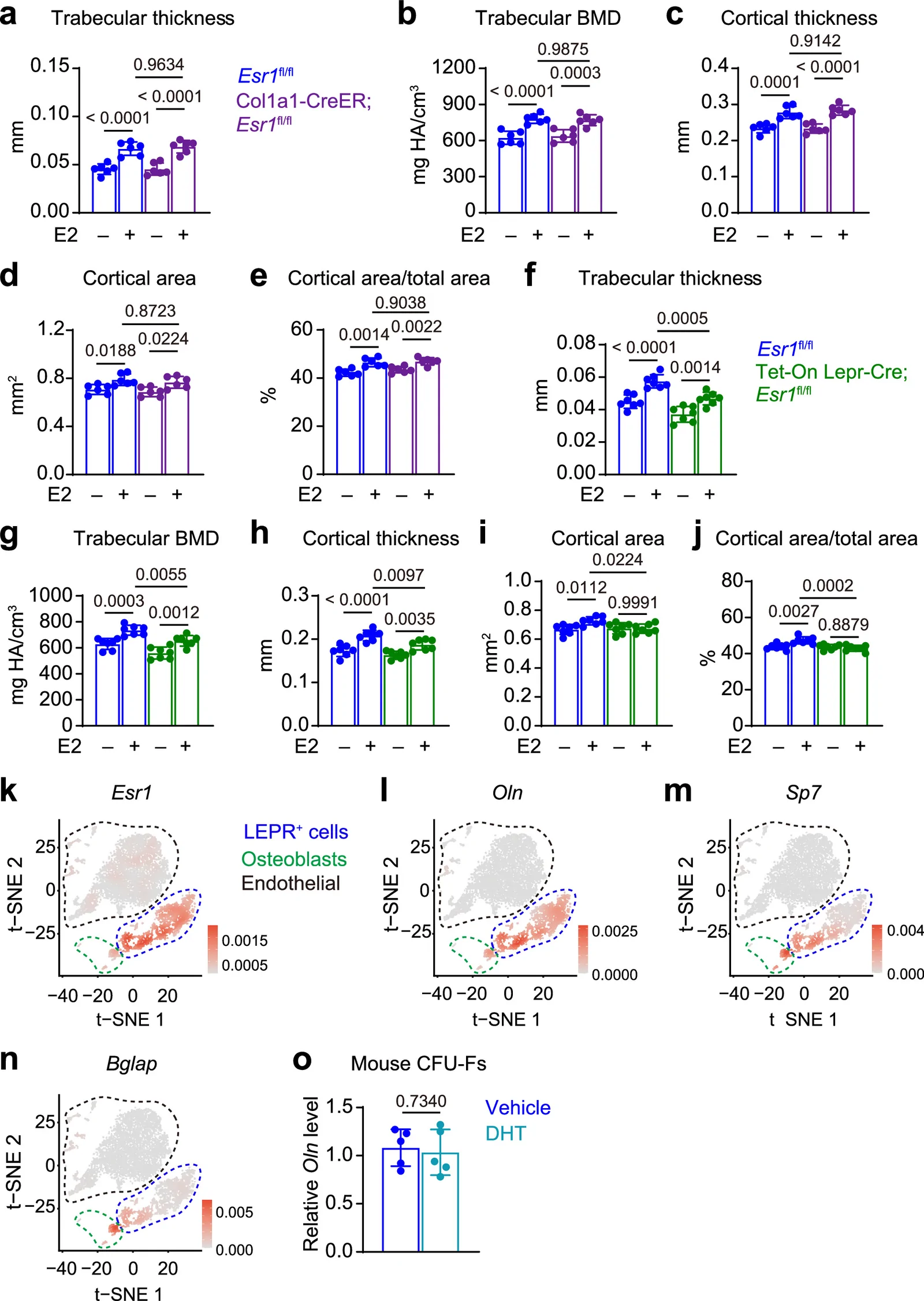
E2-induced bone anabolism is attenuated by *Esr1* deletion in LEPR^+^ cells but not by deletion in osteoblasts/osteocytes. (**a**–**e**) microCT analyses of trabecular thickness (**a**), trabecular bone mineral density (BMD) (**b**), cortical thickness (**c**), cortical area (**d**) and cortical area/total area (**e**) in Col1a1-CreER; *Esr1*^fl/fl^ mice and littermate controls following tamoxifen administration, OVX at 2 months of age, and subsequent treatment with vehicle or E2 (n = 6 mice per genotype per treatment; 3 independent experiments). (**f**–**j**) microCT analyses of trabecular thickness (**f**), trabecular BMD (**g**), cortical thickness (**h**), cortical area (**i**) and cortical area/total area (**j**) in Tet-On Lepr-Cre; *Esr1*^fl/fl^ mice and littermate controls administered doxycycline beginning at 2 months of age, subjected to OVX, and treated with vehicle or E2 for 4 weeks (n = 7 mice per genotype per treatment; 4 independent experiments). (**k**–**n**) Single-cell RNA-seq analysis of bone marrow niche cell populations showing the expression of *Esr1* (k), *Oln*/*Clec11a* (l), *Sp7* (m) and *Bglap* (**n**) in LEPR^+^ cells. (**o**) qPCR analysis of *Oln* transcript levels in CFU-Fs treated with vehicle or dihydrotestosterone (DHT) (n = 5 mice; 5 independent experiments). Data are presented as mean ± s.d. Statistical significance was assessed using two-way ANOVAs followed by Šídák’s multiple comparisons adjustment (**a**–**j**), or Student’s t-test (**o**). All tests were two-sided. NS, *P* > 0.05. Source data

Because *Esr1*-expressing LEPR^+^ cells greatly outnumber *Esr1*-expressing osteoblasts and osteocytes, we predicted that E2-induced bone anabolism would be preserved after osteoblast/osteocyte-specific *Esr1* deletion but attenuated after deletion in LEPR^+^ cells. Consistent with this prediction, osteoblast/osteocyte-specific *Esr1* deletion did not reduce E2-induced bone mass increases in ovariectomized mice (Fig. 4b,c and Extended Data Fig. 5a–e), whereas LEPR^+^ cell-specific deletion markedly attenuated the response (Fig. 4d,e and Extended Data Fig. 5f–j). These results support a role for E2-mediated regulation earlier in the osteogenic differentiation trajectory than previously appreciated.

### E2–ERα signaling promotes transcription of the osteogenic growth-factor gene *Oln* in LEPR^+^ cells

Previous studies in osteoblasts and osteocytes showed that E2–ERα signaling alters transcription of osteogenesis-related genes^16,24^. Given our finding that E2 promotes osteogenic differentiation of LEPR^+^ cells, we used RNA-seq to identify E2-responsive genes in LEPR^+^ cells isolated from 3-month-old wild-type mice treated with E2 or vehicle for 4 weeks. Among osteogenesis-related genes, 22 were significantly upregulated by E2 (Fig. 4f). *Oln*, encoding osteolectin (OLN), emerged as a prominent candidate because OLN is produced by LEPR^+^ cells^29^ and promotes their osteogenic differentiation^29–32^. Given the heterogeneity of LEPR^+^ cells^30,33–35^, we reanalyzed a published single-cell RNA-seq dataset of adult bone marrow stromal cells^33^. *Esr1*, *Oln*, *Sp7* and *Bglap* were expressed at higher levels in an overlapping LEPR^+^ cell subset (Extended Data Fig. 5k–n), placing ERα expression and *Oln* transcription within LEPR^+^ cells with osteogenic features.

**Extended Data Fig. 6:**
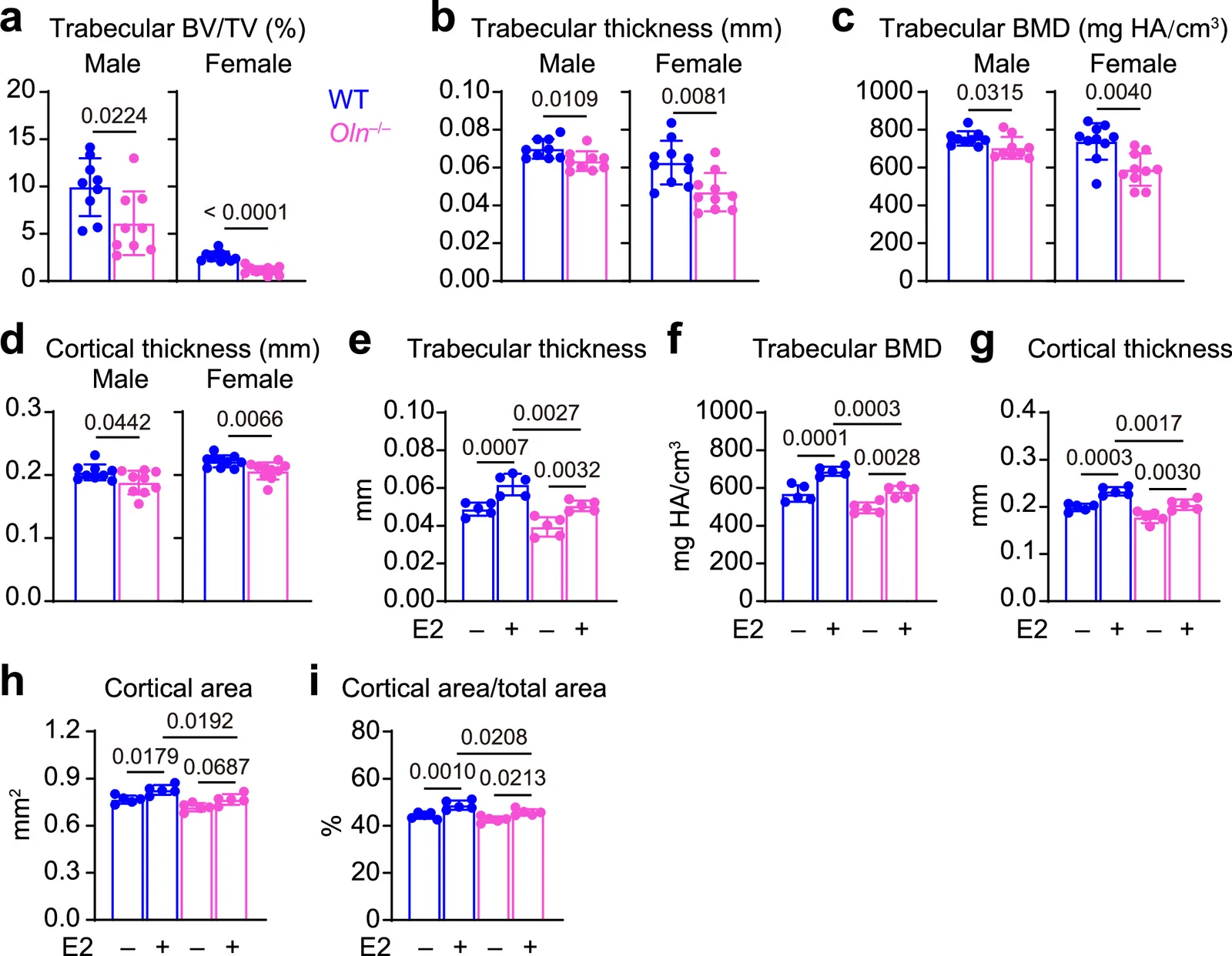
*Oln* deficiency reduces adult bone mass and attenuates E2-induced bone anabolism. (**a**–**d**) microCT analyses of trabecular bone volume (**a**), trabecular thickness (**b**), trabecular BMD (**c**) in the distal femur metaphysis, and cortical thickness (**d**) in the femoral mid-diaphysis of 12-month-old *Oln*^−*/*−^ mice and sex-matched littermate controls (n = 9 mice per genotype per sex; 3 independent experiments). (**e**–**i**) microCT analyses of trabecular thickness (**e**), and trabecular BMD (**f**) in the distal femur metaphysis, and cortical thickness (**g**), cortical area (**h**) and cortical area/total area (**i**) in the femoral mid-diaphysis in female *Oln*^−*/*−^ mice and littermate controls subjected to OVX at 2 months of age and treated with vehicle or E2 for 4 weeks (n = 5 mice per genotype per treatment; 3 independent experiments). Data are presented as mean ± s.d. Statistical significance was assessed using Student’s t-test (**a**, **b**, **d**), Mann-Whitney tests (**c**), or two-way ANOVAs followed by Šídák’s multiple comparisons adjustment (**e**–**i**). All tests were two-sided. NS, *P* > 0.05. Source data

Consistent with estrogen-dependent regulation of *Oln* expression, *Oln* transcript levels were significantly reduced in LEPR^+^ cells from ovariectomized mice relative to sham-operated controls (Fig. 4g), whereas dihydrotestosterone (DHT) did not increase *Oln* transcription in CFU-Fs (Extended Data Fig. 5o), supporting a selective role for E2–ERα signaling. MicroCT analysis of 12-month-old *Oln*^−/−^ mice showed that *Oln* deficiency caused a more pronounced reduction in bone mass in females than males relative to sex-matched littermate controls (Extended Data Fig. 6a–d), suggesting that adult female bone maintenance is particularly sensitive to OLN loss. In ovariectomized mice, E2 increased bone mass in both wild-type littermate controls and *Oln*^−/−^ mice, although the response was attenuated in the mutants (Fig. 4h and Extended Data Fig. 6e–i). These results support a contributory role for OLN in E2-induced bone anabolism.

**Extended Data Fig. 7:**
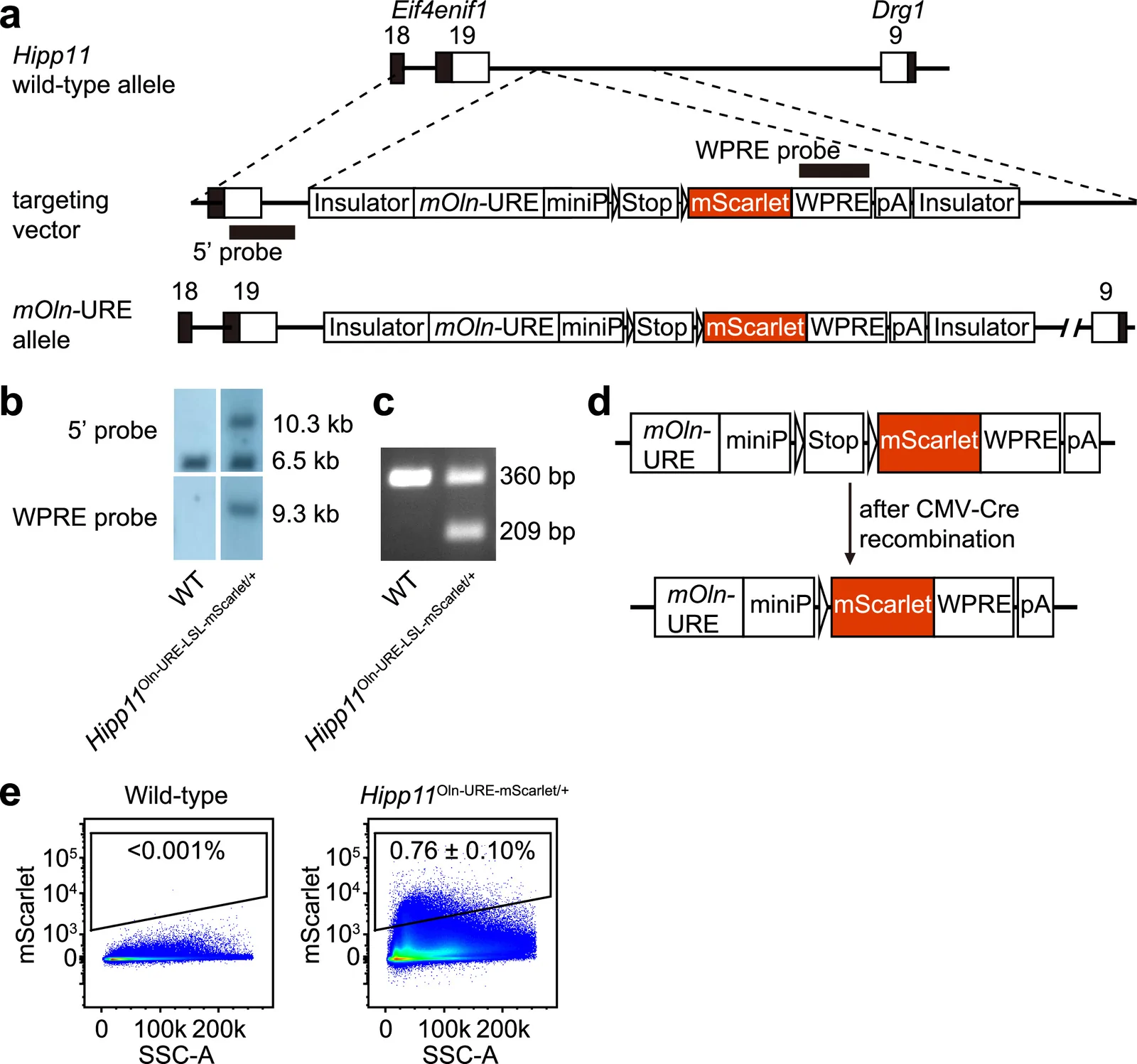
Generation and validation of a *Hipp11*^Oln−URE−LSL−mScarlet^ reporter allele. (**a**–**c**) Targeting strategy for generation of a *Hipp11*^Oln−URE−LSL−mScarlet^ reporter allele. The mouse *Hipp11* intergenic locus was modified by inserting an Oln-URE-HSP68-minimal promoter-loxP-STOP-loxP-mScarlet-WPRE-bGH cassette. Open boxes indicate untranslated regions and black boxes indicate translated regions (a). The correctly targeted founder mouse (F0) was identified by Southern blotting using 5’ and WPRE probes (black bars in a) (**b**) (representative of three independent experiments). PCR genotyping of genomic DNA confirmed germline transmission of the *Hipp11*^Oln−URE−LSL−mScarlet^ allele (**c**) (representative of three independent experiments). Mice were backcrossed at least three times onto a C57BL/6 J background before analysis. (**d**) Breeding with CMV-Cre mice resulted in germline excision of the loxP-STOP-loxP cassette, generating *Hipp11*^Oln−URE−mScarlet^ mice in which mScarlet expression was driven by the *Oln*–URE and no longer required Cre recombination. (**e**) Flow cytometric analysis of enzymatically dissociated bone marrow from *Hipp11*^Oln−URE−mScarlet^ mice revealed that only 0.76 ± 0.10% of bone marrow cells were mScarlet^+^ (n = 3 mice; 3 independent experiments). Data are presented as mean ± s.d. Source data

We next used cleavage under targets and tagmentation (CUT&Tag) to assess whether E2 directly regulates *Oln* transcription through ERα. In mouse and human CFU-Fs cultured under steroid-depleted conditions, ERα occupancy at an upstream *Oln/OLN* regulatory region was minimal with vehicle and strongly increased after E2 treatment, whereas the signal within the gene body did not increase (Fig. 4i,j). We therefore tested this E2-induced ERα-bound region as a candidate upstream regulatory element (URE) by generating a *Hipp11/Igs2*^Oln−URE−mScarlet^ reporter allele containing a ~1-kb fragment spanning the CUT&Tag peak upstream of a minimal promoter (Extended Data Fig. 7a–d). mScarlet^+^ cells constituted only 0.76% ± 0.10% of enzymatically dissociated bone marrow cells (Extended Data Fig. 7e), whereas 91% ± 2.4% of LEPR^+^ cells and 94% ± 5.0% of *Esr1*-GFP^+^ cells were mScarlet^+^ (Fig. 4k,l). These data indicate that this ERα-bound region functions as an active URE in vivo, with URE-driven mScarlet expression enriched in LEPR^+^ and *Esr1*-GFP^+^ cells. Collectively, these results established that E2–ERα signaling promotes transcription of the osteogenic growth-factor gene *Oln* in LEPR^+^ cells.

### ERα signaling in LEPR^+^ cells maintains bone mass during adulthood

**Fig. 5:**
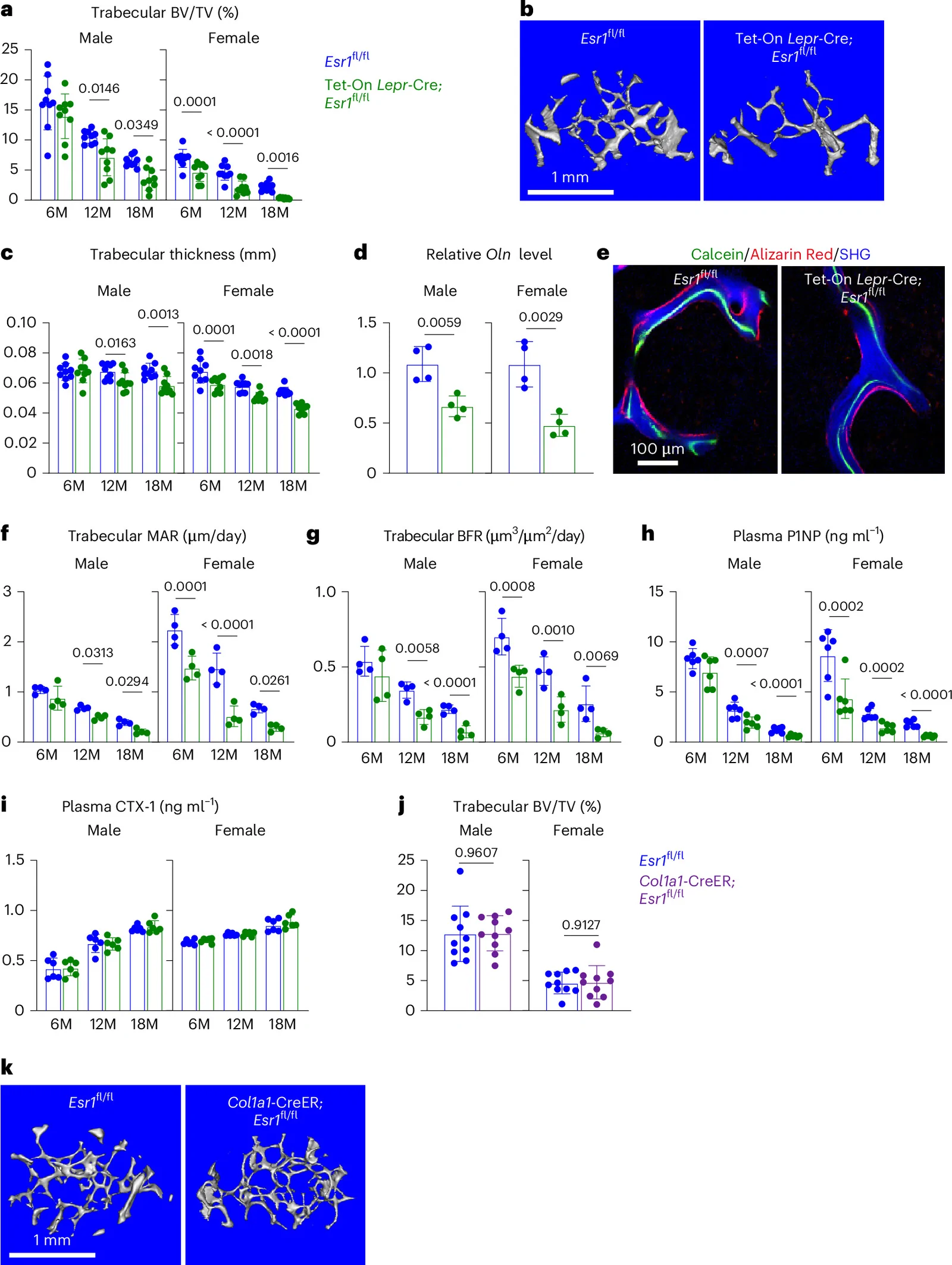
ERα signaling in LEPR^+^ cells is required for maintenance of adult bone mass. Both Tet‑On *Lepr*‑Cre; *Esr1*^fl/fl^ mice and *Esr1*^fl/fl^ littermate controls received doxycycline from 2 to 3 months of age to induce *Esr1* deletion. Sex-matched *Esr1*^fl/fl^ littermates served as controls. **a**–**c**, microCT analysis of the distal femur metaphysis at 6, 12 and 18 months of age showing trabecular bone volume (**a**), representative trabecular microCT images from 12-month-old female mice (**b**) and trabecular thickness (**c**) (*n* = 9 mice per genotype per sex per time point; three independent experiments). **d**, *Oln* transcript levels (reverse transcription–qPCR) in LEPR^+^ cells isolated at 12 months of age (*n* = 4 mice per genotype per sex; four independent experiments). **e**−**g**, Representative calcein/Alizarin Red double-labeling images from 12-month-old female mice (**e**) and quantification of trabecular MAR (**f**) and BFR (**g**) in the femur metaphysis at 6, 12 and 18 months of age (*n* = 4 mice per genotype per sex per time point; four independent experiments). **h**, Plasma P1NP levels at the indicated ages (*n* = 6 mice per genotype per sex per time point; three independent experiments). **i**, Plasma CTX‑1 levels at the indicated ages (*n* = 6 mice per genotype per sex per time point; three independent experiments). **j**,**k**, microCT analysis of the distal femur metaphysis in *Col1a1*‑CreER; *Esr1*^fl/fl^ mice and sex-matched *Esr1*^fl/fl^ littermate controls at 12 months of age following tamoxifen administration at 2 months, showing trabecular bone volume (**j**) and representative trabecular microCT images (**k**) (*n* = 10 mice per genotype per sex; four independent experiments). All data represent mean ± s.d. Statistical significance was determined by two-way ANOVA followed by Tukey’s multiple-comparisons adjustment (**a**, **c** and **f**–**i**) or Student’s *t*-test (**d** and **j**). All tests were two sided. NS, *P* > 0.05. Source data

**Extended Data Fig. 8:**
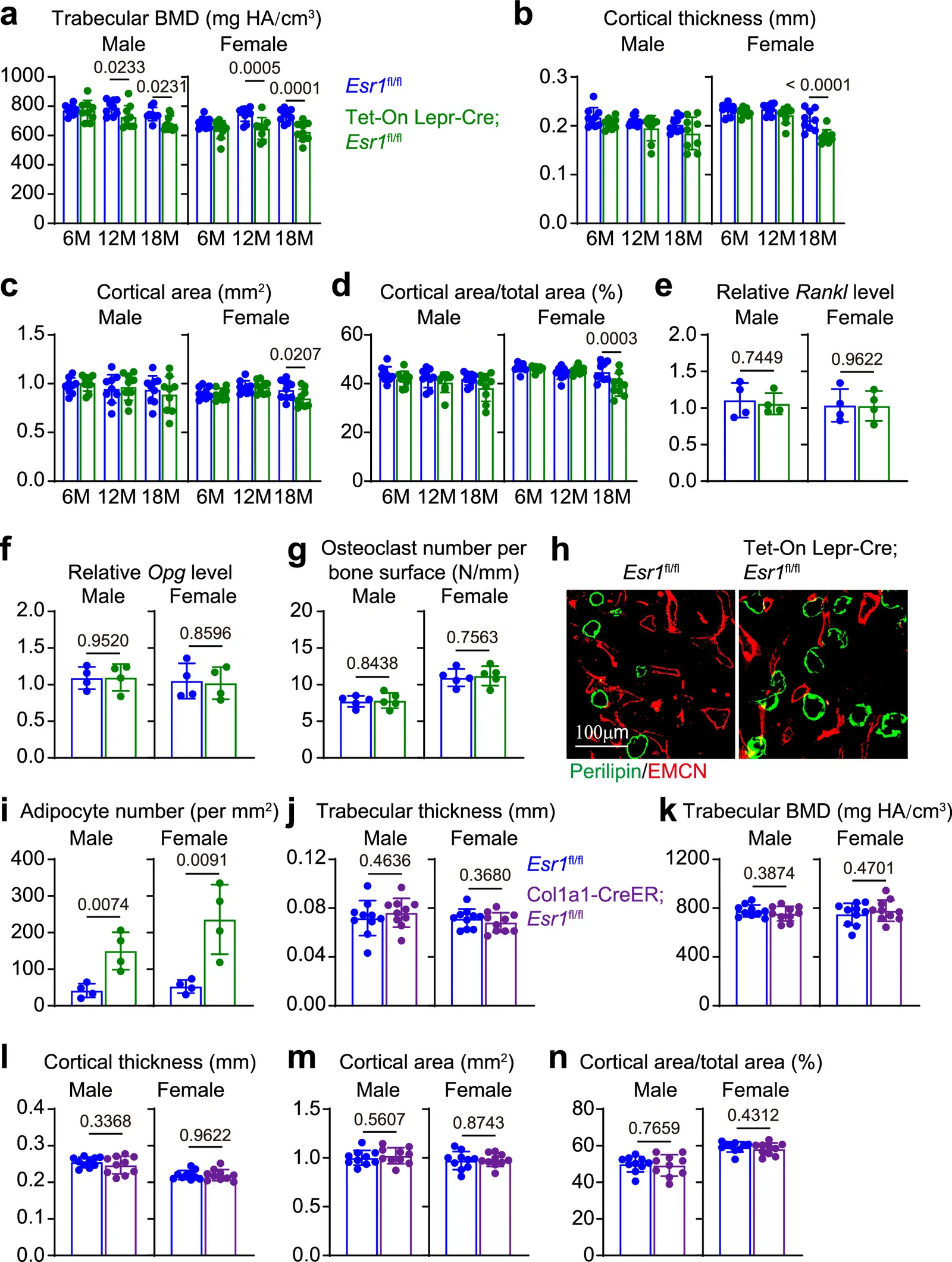
ERα signaling in LEPR^+^ cells, but not osteoblasts or osteocytes, maintains adult bone mass. (**a**–**d**) microCT analyses of trabecular BMD (**a**) in the distal femur metaphysis, and cortical thickness (**b**), cortical area (**c**) and cortical area/total area (**d**) in the femoral mid-diaphysis in Tet-On Lepr-Cre; *Esr1*^fl/fl^ mice and sex-matched littermate controls at 6, 12, and 18 months of age following doxycycline administration at 2 months of age (n = 9 mice per genotype per sex per time point; 3 independent experiments). (**e**, **f**) qPCR analysis of *Rankl* (**e**) and *Opg* (**f**) transcript levels in sorted LEPR^+^ cells from Tet-On Lepr-Cre; *Esr1*^fl/fl^ mice and sex-matched littermate controls at 12 months of age following doxycycline administration at 2 months of age (n = 4 mice per genotype per sex; 4 independent experiments). (**g**–**i**) Quantification of osteoclast number on trabecular bone surfaces in the distal femur metaphysis (**g**), and representative images from females (**h**) and quantification (**i**) of adipocyte number in metaphyseal bone marrow from Tet-On Lepr-Cre; *Esr1*^fl/fl^ mice and sex-matched littermate controls at 12 months of age following doxycycline administration at 2 months of age (n = 5 (**g**) or 4 (**i**) mice per genotype per sex; 5 (**g**) or 4 (**i**) independent experiments). (**j**–**n**) microCT analyses of trabecular thickness (**j**) and trabecular BMD (**k**) in the distal femur metaphysis, and cortical thickness (**l**), cortical area (**m**) and cortical area/total area (**n**) in the femoral mid-diaphysis in Col1a1-CreER; *Esr1*^fl/fl^ mice and sex-matched littermate controls at 12 months of age after tamoxifen administration at 2 months of age (n = 10 mice per genotype per sex; 4 independent experiments). Data are presented as mean ± s.d. Statistical significance was assessed using two-way ANOVAs followed by Šídák’s multiple comparisons adjustment (**a**–**d**), or Student’s t-tests (**e**–**g, i**–**n**). All tests were two-sided. NS, *P* > 0.05. Source data

Having established that E2–ERα signaling promotes *Oln* transcription in LEPR^+^ cells, we tested whether ERα signaling in this population maintains *Oln* expression and bone mass during adulthood. We induced *Esr1* deletion in Tet‑On *Lepr*‑Cre; *Esr1*^fl/fl^ mice by administering doxycycline at 2 months of age. Both genotypes exhibited age-associated bone loss, but *Esr1* deletion in LEPR^+^ cells accelerated this decline. Bone mass was significantly reduced by 6 months in females and at 12 and 18 months in both sexes (Fig. 5a–c and Extended Data Fig. 8a–d). *Oln* transcripts were also reduced in LEPR^+^ cells following *Esr1* deletion (Fig. 5d), and the skeletal phenotype resembled the accelerated adult bone loss observed in *Oln*^−/−^ mice^29^, consistent with ERα promoting *Oln* transcription to maintain adult bone mass.

We next assessed whether this accelerated bone loss reflects decreased bone formation and/or increased resorption. Calcein and Alizarin Red double labeling showed reduced mineral apposition and bone formation rates (BFRs) following *Esr1* deletion in LEPR^+^ cells (Fig. 5e–g), accompanied by reduced plasma P1NP but unchanged CTX-1 levels (Fig. 5h,i). *Esr1* deletion also did not alter *Rankl* or *Opg* transcription in LEPR^+^ cells or increase osteoclast number on trabecular bone surfaces (Extended Data Fig. 8e–g), but increased bone marrow adipocyte number (Extended Data Fig. 8h,i), consistent with reduced osteogenic differentiation and increased adipocyte formation. Thus, the reduced bone mass reflects impaired bone formation rather than increased resorption. By contrast, osteoblast/osteocyte-restricted *Esr1* deletion using *Col1a1*-CreER at 2 months of age did not reduce bone mass in either sex at 12 months (Fig. 5j,k and Extended Data Fig. 8j–n). Together, these results establish that ERα signaling in LEPR^+^ cells, rather than osteoblasts and osteocytes, is required to maintain adult bone mass by promoting bone formation.

### Statins promote *Oln* transcription by restricting protein prenylation

**Fig. 6:**
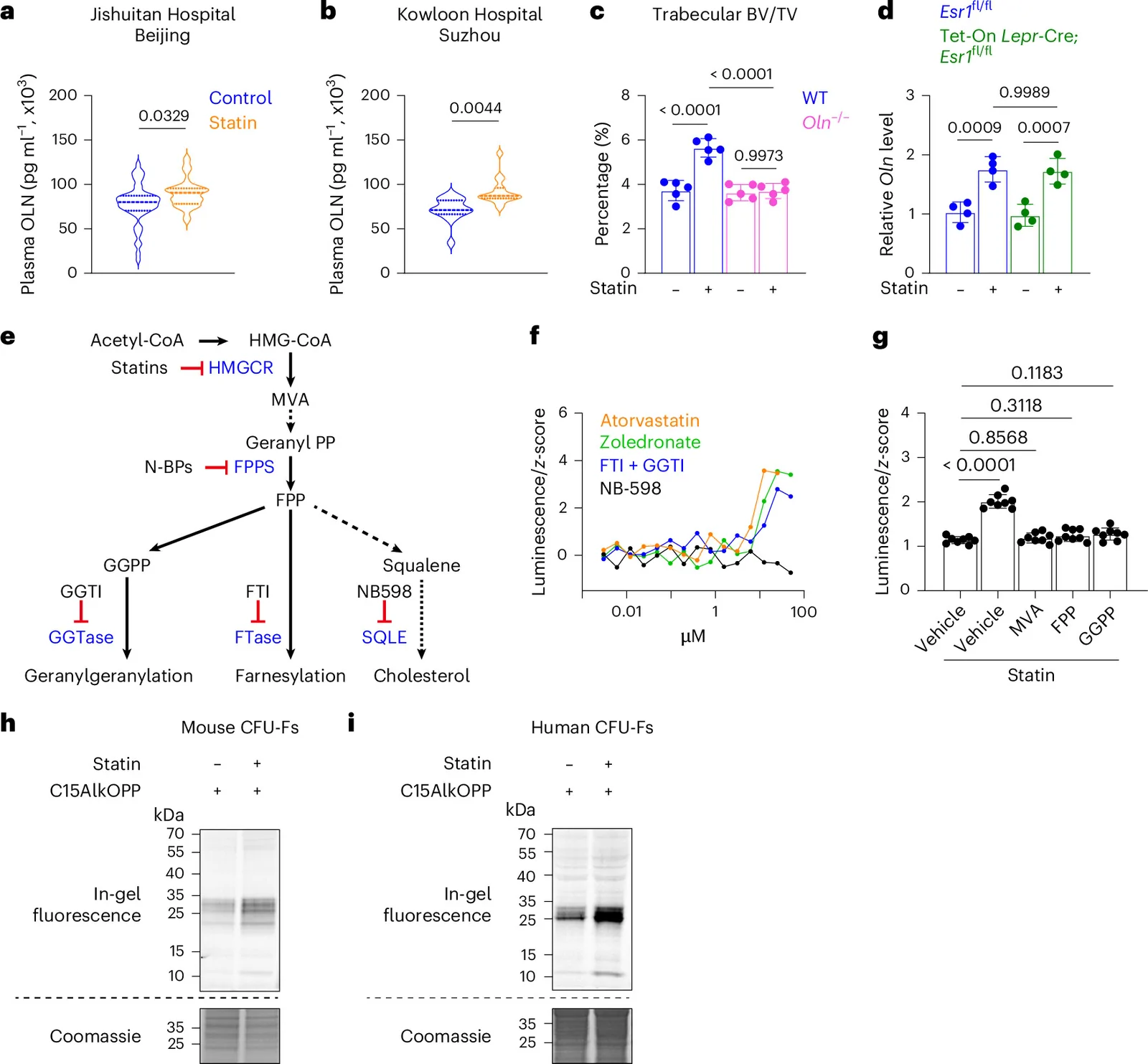
Statins increase OLN abundance through inhibition of the mevalonate pathway and protein prenylation. **a**,**b**, Multiplex immunoassay analysis showing elevated circulating OLN levels in postmenopausal statin users compared with nonusers at Beijing Jishuitan Hospital (**a**; control, *n* = 51; statin, *n* = 47) and Kowloon Hospital (**b**; control, *n* = 15; statin, *n* = 16). **c**, microCT analysis of trabecular bone volume in female *Oln*^−/−^ mice and littermate controls subjected to OVX at 2 months of age and treated with atorvastatin or vehicle for 8 weeks (*n* = 5 mice per genotype per treatment; three independent experiments). **d**, *Oln* transcript levels in LEPR^+^ cells isolated from ovariectomized Tet-On *Lepr*-Cre; *Esr1*^fl/fl^ mice and littermate controls treated with vehicle or atorvastatin following doxycycline induction (*n* = 4 mice per genotype per treatment; three independent experiments). **e**, Schematic representation of the mevalonate (MVA) synthesis pathway with key enzymes and inhibitors highlighted. **f**, Atorvastatin, zoledronate (a nitrogen-containing bisphosphonate) or combined inhibition of farnesyltransferase (FTI-277) and geranylgeranyltransferase (GGTI-298) increased *Oln*–NanoLuc activity in a dose-dependent manner (mean of three independent experiments). **g**, Supplementation with geranylgeranyl pyrophosphate (GGPP) or farnesyl pyrophosphate (FPP) blocked atorvastatin-induced promotion of *Oln*–NanoLuc activity (*n* = 8 biological replicates per treatment; four independent experiments). **h**,**i**, In-gel fluorescence imaging of C15AlkOPP-labeled prenylated proteins in mouse (**h**) and human (**i**) CFU-Fs demonstrated increased probe incorporation following atorvastatin treatment, consistent with reduced competition from endogenous isoprenoid donors (representative of three independent experiments). Data are presented as the mean ± s.d. Statistical significance was determined by a Mann–Whitney test with multiple-testing correction by the Benjamini, Krieger and Yekutieli two-stage linear step-up procedure (5% false discovery rate) (**a** and **b**), two-way ANOVA followed by Šídák’s multiple-comparisons adjustment (**c** and **d**) or one-way ANOVA followed by Dunnett’s multiple-comparisons adjustment (**g**). All tests were two sided. NS, *P* > 0.05. Source data

**Extended Data Fig. 9:**
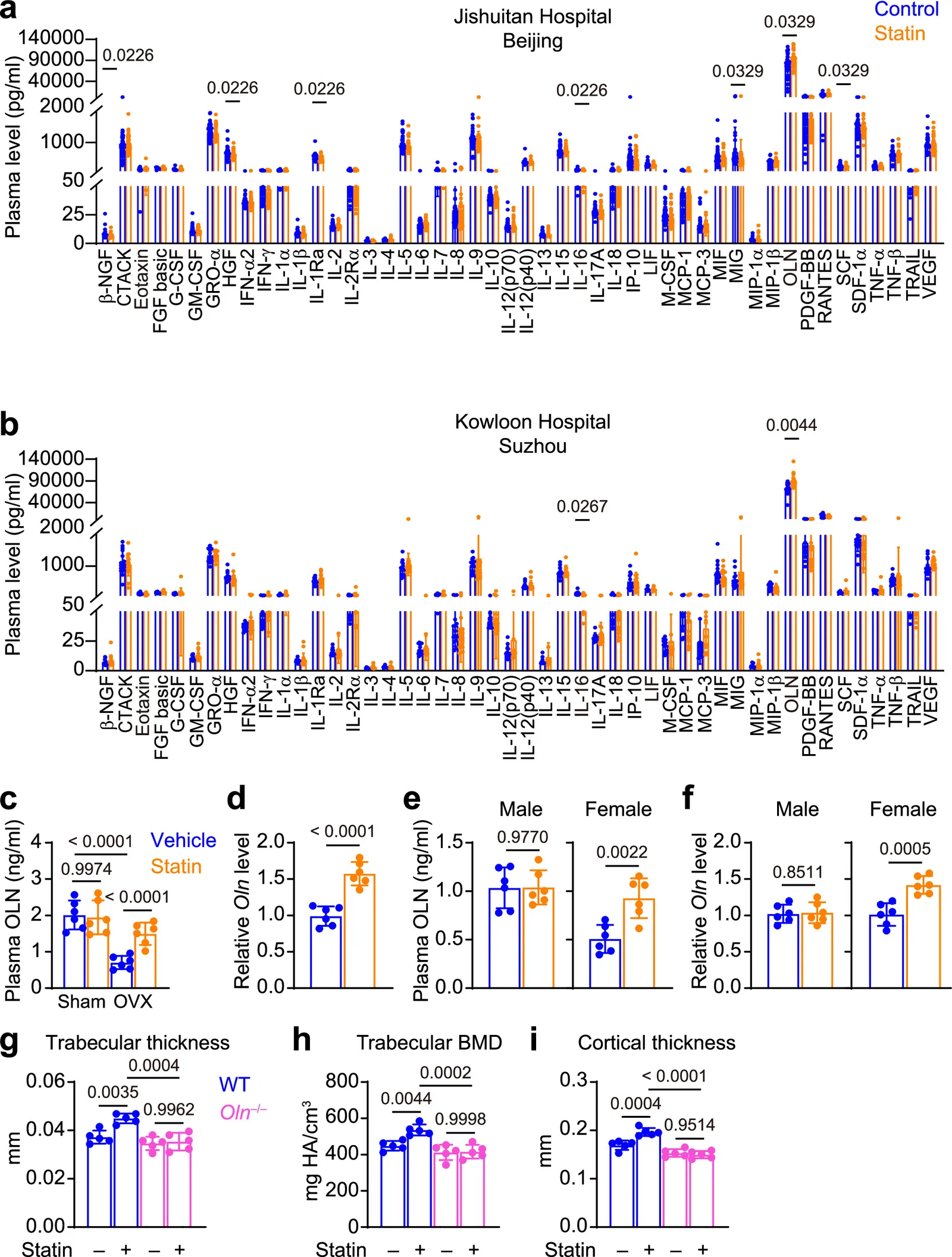
OLN is required for statin-induced bone anabolism. (**a** and **b**) Plasma protein concentrations measured by multiplex immunoassay using samples from postmenopausal women with or without statin use from Beijing Jishuitan Hospital (a; control, n = 51; statin, n = 47) and Kowloon Hospital (b; control, n = 15; statin, n = 16). (**c**) Plasma OLN levels in sham-operated and ovariectomized female mice treated with vehicle or atorvastatin (n = 6 mice per group; 3 independent experiments). (**d**) *Oln* transcript levels in sorted LEPR^+^ cells were higher in ovariectomized mice treated with atorvastatin compared with vehicle-treated controls (n = 6 mice per treatment; 3 independent experiments). (**e**) Plasma OLN levels were higher in aged female mice treated with atorvastatin relative to vehicle-treated controls, but not in aged male mice (n = 6 mice per treatment per sex; 3 independent experiments). (**f**) *Oln* transcript levels in sorted LEPR^+^ cells were higher in aged female mice treated with atorvastatin relative to vehicle-treated controls, but not in aged male mice (n = 6 mice per treatment per sex; 3 independent experiments). (**g**–**i**) microCT analyses of trabecular thickness (**g**), trabecular bone mineral density (BMD) (**h**), and cortical thickness (**i**) in female *Oln*^−*/*−^ mice and littermate controls subjected to OVX at 2 months of age and treated with vehicle or atorvastatin (n = 5 mice per genotype per treatment; 3 independent experiments). Data are presented as mean ± s.d. Statistical significance was assessed using Mann-Whitney tests with multiple-testing correction by the Benjamini, Krieger and Yekutieli two-stage linear step-up procedure (5% false discovery rate) (**a**, **b**), two-way ANOVAs followed by Šídák’s multiple comparisons adjustment (**c**, and **g**–**i**), or Student’s t-tests (**d**–**f**). All tests were two-sided. NS, *P* > 0.05. Source data

Having established that E2–ERα signaling promotes *Oln* transcription in LEPR^+^ cells and that OLN contributes to E2-induced bone anabolism, we next investigated whether OLN also contributes to statin-induced bone anabolism. Multiplex immunoassay analysis of plasma samples from postmenopausal women revealed significantly higher circulating OLN levels in statin users than nonusers in both cohorts (Fig. 6a,b). Among the 48 proteins examined, OLN and interleukin-16 were the only factors that differed significantly between the groups in both cohorts (Extended Data Fig. 9a,b). Atorvastatin likewise increased plasma OLN levels and *Oln* transcripts in LEPR^+^ cells in ovariectomized and aged female mice (Extended Data Fig. 9c–f). These data establish that statins promote *Oln* transcription in LEPR^+^ cells under estrogen-deficient conditions.

To test whether OLN is required for the statin-induced bone anabolism, we examined ovariectomized *Oln*^−*/*−^ mice. MicroCT analysis revealed no difference in bone mass between atorvastatin-treated and vehicle-treated *Oln*^−*/*−^ mice (Fig. 6c and Extended Data Fig. 9g–i), demonstrating that OLN is required for statin-induced bone anabolism. Given our finding that E2–ERα signaling promotes *Oln* transcription in LEPR^+^ cells, we next asked whether ERα also contributes to the statin-induced promotion of *Oln* transcription. In ovariectomized mice treated with atorvastatin, *Oln* expression in LEPR^+^ cells did not differ between mice with conditional deletion of *Esr1* and littermate controls (Fig. 6d), indicating that statin-induced promotion of *Oln* transcription occurs independently of ERα.

**Extended Data Fig. 10:**
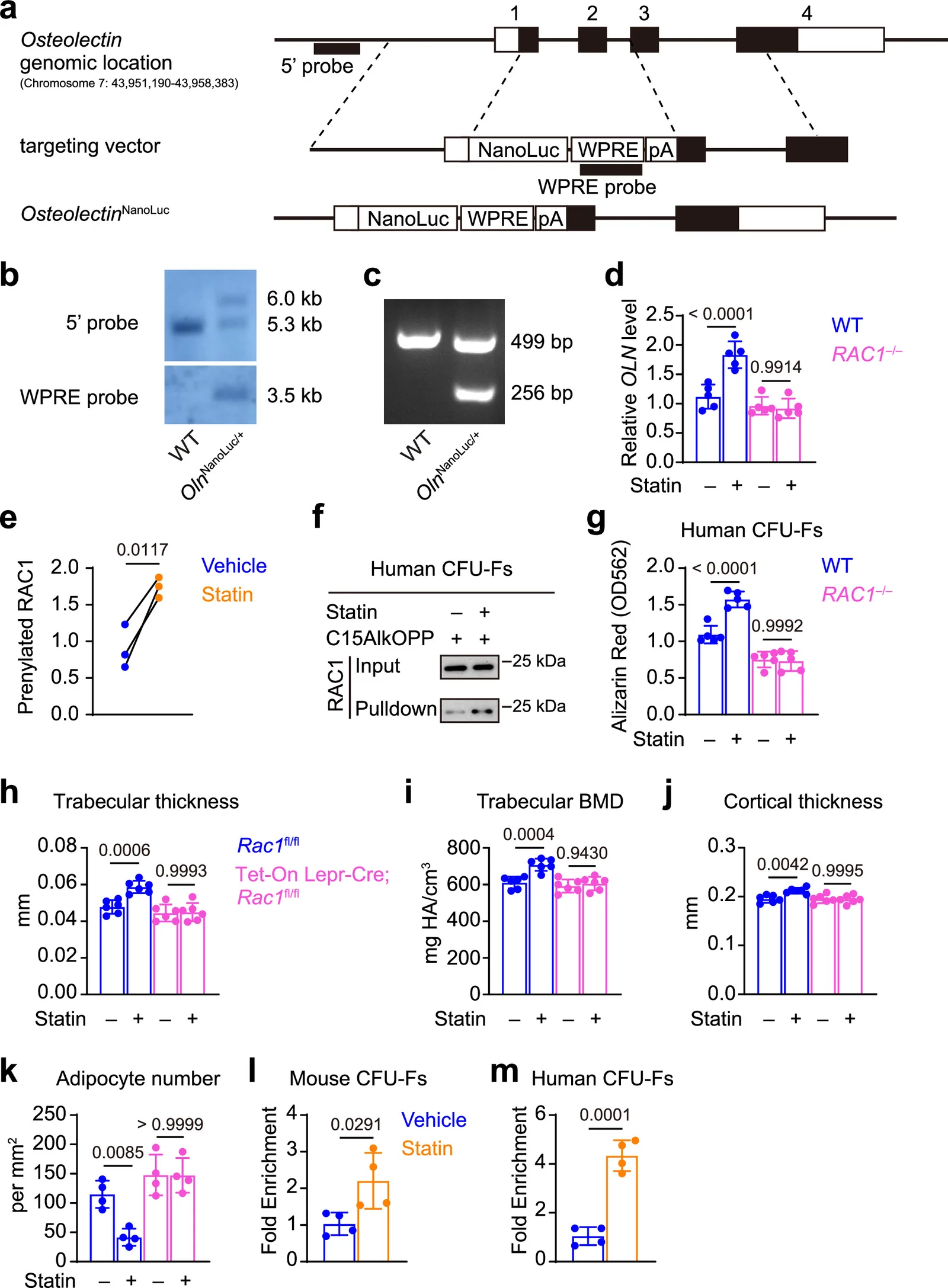
RAC1 in LEPR^+^ cells is required for statin-induced bone anabolism. (**a**–**c**) Generation and validation of the *Oln*^NanoLuc/+^ reporter allele, created by inserting a *NanoLuciferase–WPRE–bGH* cassette into the endogenous *Oln* locus. Open boxes represent untranslated regions, and filled boxes denote coding regions (**a**). Correct targeting of the F0 founder was confirmed by Southern blotting using 5’ and WPRE probes (**b**) (representative of three independent experiments), and PCR-based genotyping verified germline transmission of the reporter allele (**c**) (representative of three independent experiments). (**d**) Atorvastatin increased *OLN* transcript levels in control but not *RAC1*-deficient human CFU-Fs (n = 5 human samples per treatment; 4 independent experiments). (**e**) Quantification of C15AlkOPP labeling of immunoprecipitated RAC1 from murine CFU-Fs following vehicle or atorvastatin treatment, corresponding to Fig. 7b (n = 3 mice; 3 independent experiments). (**f**) C15AlkOPP labeling of immunoprecipitated RAC1 from human CFU-Fs revealed increased probe incorporation following atorvastatin treatment, consistent with restricted endogenous RAC1 prenylation (representative of 3 independent experiments). (**g**) Atorvastatin promoted osteogenic differentiation in control but not *RAC1*-deficient human CFU-Fs (n = 5 human samples per treatment; 4 independent experiments). (**h**–**j**) microCT analyses of trabecular thickness (**h**) and trabecular bone mineral density (BMD) (**i**) in the distal femur metaphysis, and cortical thickness (**j**) in the femoral mid-diaphysis of female Tet-On Lepr-Cre; *Rac1*^fl/fl^ mice and littermate controls administered doxycycline, subjected to OVX at 3 months of age, and treated with vehicle or atorvastatin (n = 6 mice per genotype per treatment; 3 independent experiments). (**k**) Quantification of adipocyte number in metaphyseal bone marrow from Tet-On Lepr-Cre; *Rac1*^fl/fl^ mice and littermate controls administered doxycycline, subjected to OVX at 3 months of age, and treated with vehicle or atorvastatin (n = 4 mice per genotype per treatment; 4 independent experiments). (**l**, **m**) ChIP-qPCR analysis confirmed increased NF-κB binding at the murine *Oln* locus (l) and the human *OLN* locus (m) following atorvastatin treatment (n = 4 mice for l; n = 4 human samples for m; 4 independent experiments). Data are presented as mean ± s.d. Statistical significance was assessed using two-way ANOVAs followed by Šídák’s multiple comparisons adjustment (**d**, and **g**–**k**), or Student’s t-tests (**e**, **l** and **m**). All tests were two-sided. NS, *P* > 0.05. Source data

We next examined whether this ERα-independent effect reflects inhibition of the mevalonate pathway. Statins inhibit 3-hydroxy-3-methyl-glutaryl-CoA reductase, the rate-limiting enzyme of this pathway, reducing the production of isoprenoid intermediates required for non-sterol metabolic processes^20^. To assess transcription from the endogenous *Oln* locus, we generated an *Oln*^NanoLuc/+^ reporter allele and measured *Oln*–NanoLuc activity in CFU-Fs following pharmacological perturbation of the pathway (Fig. 6e and Extended Data Fig. 10a–c). Atorvastatin increased *Oln*–NanoLuc activity in a dose-dependent manner, and inhibition of farnesyl pyrophosphate synthase with a nitrogen-containing bisphosphonate phenocopied this response (Fig. 6f). By contrast, inhibition of sterol synthesis at squalene epoxidase with NB-598 had no effect (Fig. 6f). Combined inhibition of farnesyltransferase and geranylgeranyltransferase with FTI-277 and GGTI-298 also dose-dependently increased *Oln*–NanoLuc activity (Fig. 6f). Moreover, supplementation with geranylgeranyl pyrophosphate or farnesyl pyrophosphate blocked the atorvastatin-induced increase (Fig. 6g), demonstrating that prenyl donor availability limits statin-induced *Oln* transcription. These data establish that statins promote *Oln* transcription by limiting the abundance of isoprenoid substrates required for protein prenylation rather than by inhibiting sterol synthesis.

Farnesyl pyrophosphate is a precursor of geranylgeranyl pyrophosphate, and both serve as lipid donors for prenylation of CAAX-motif-containing proteins, including many small GTPases^36,37^. To assess whether statin treatment alters protein prenylation in CFU-Fs, we performed metabolic labeling of prenylated proteins in mouse and human CFU-Fs using the clickable farnesyl pyrophosphate analog C15AlkOPP^38^. In-gel fluorescence revealed prominent C15AlkOPP-labeled bands in the 20–30-kDa range, consistent with the molecular masses of small GTPases (Fig. 6h,i). Atorvastatin increased probe incorporation within this range, consistent with reduced competition from endogenous isoprenoid donors. Collectively, these results are consistent with reduced availability of endogenous isoprenoid prenyl donors in statin-treated CFU-Fs, further supporting a prenylation-dependent mechanism for statin-induced promotion of *Oln* transcription.

### The small GTPase RAC1 is required for statin-induced bone anabolism

**Fig. 7:**
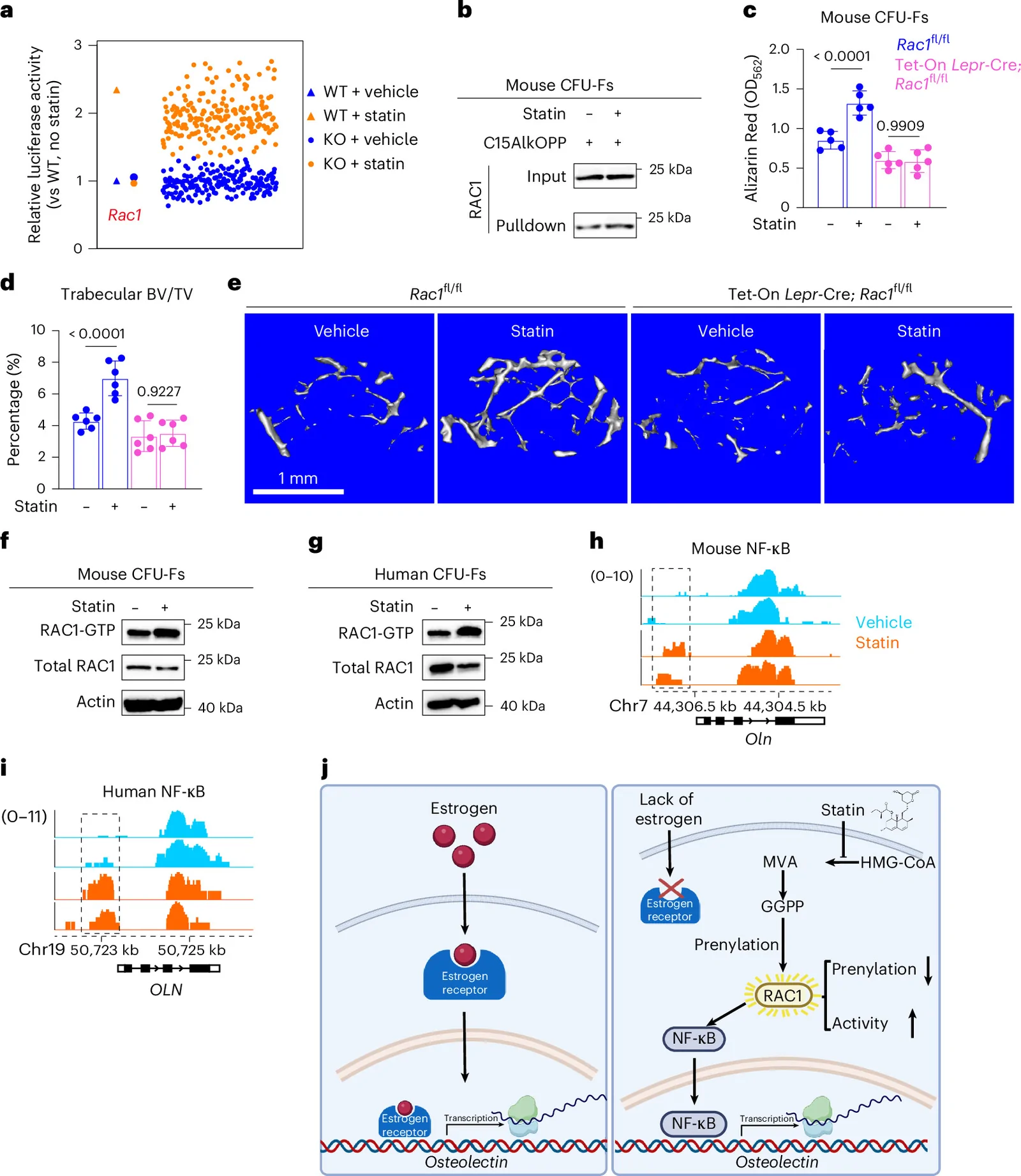
Statins restrict RAC1 prenylation and promote NF-κB-mediated *Oln* transcription. **a**, CRISPR-based genetic screening in *Rosa26*^Cas9/+^; *Oln*^NanoLuc/+^ CFU-Fs identified *Rac1* as a critical determinant of atorvastatin-induced promotion of *Oln*–NanoLuc activity (triangle, control gRNA; circle, gRNAs targeting candidate prenylated proteins; blue, vehicle; orange, atorvastatin; mean of three independent experiments). **b**, C15AlkOPP labeling of immunoprecipitated RAC1 from mouse CFU-Fs showed increased probe incorporation following atorvastatin treatment, consistent with impaired RAC1 prenylation (representative of three independent experiments). **c**, Atorvastatin promoted osteogenic differentiation in control but not *Rac1*-deficient mouse CFU-Fs (*n* = 5 mice per genotype per treatment; three independent experiments). **d**,**e**, Quantification of trabecular bone volume (**d**) and representative microCT images (**e**) from ovariectomized Tet-On *Lepr*-Cre; *Rac1*^fl/fl^ mice and littermate controls following doxycycline induction, treated with vehicle or atorvastatin (*n* = 6 mice per genotype per treatment; three independent experiments). **f**, Atorvastatin increased GTP loading of RAC1 in mouse CFU-Fs, as determined by a pull-down assay for activated RAC1 (representative of three independent experiments). **g**, Atorvastatin increased RAC1 GTP loading in human CFU-Fs, as determined by the RAC1 pull-down assay (representative of three independent experiments). **h**,**i**, CUT&Tag analysis demonstrated NF-κB occupancy at the mouse *Oln* locus (**h**) and the human *OLN* locus (**i**), with enhanced binding following atorvastatin treatment (two independent experiments). **j**, Proposed model illustrating ERα-dependent and NF-κB-mediated promotion of *Oln* transcription, highlighting alternative upregulation of *Oln* by statins under estrogen-deficient conditions. Created in BioRender; Shen, B. https://biorender.com/5uh2ize (2026). Data are presented as the mean ± s.d. Statistical significance was determined using two-way ANOVA followed by Benjamini–Hochberg correction (**a**), or two-way ANOVA followed by Šídák’s multiple-comparisons adjustment (**c** and **d**). All tests were two sided. NS, *P* > 0.05. Source data

We next conducted a genetic screen to identify small GTPases required for statin-induced *Oln* transcription. Guided by prior literature on protein prenylation and C-terminal CAAX motifs associated with this modification^36,37^, we compiled 197 candidate proteins, including 104 small GTPases. Each candidate gene was individually deleted in CFU-Fs from *Oln*^NanoLuc/+^; *Rosa26*^Cas9/+^ mice using CRISPR–Cas9, and the effect on atorvastatin-induced *Oln*–NanoLuc activity was assessed. Thirteen deletions attenuated the response, with *Rac1* deletion producing the strongest phenotype and completely abolishing the statin-induced increase in *Oln*–NanoLuc activity (Fig. 7a). *RAC1* deletion in human CFU-Fs likewise eliminated atorvastatin-induced promotion of *OLN* transcription (Extended Data Fig. 10d). C15AlkOPP labeling of immunoprecipitated RAC1 increased after atorvastatin treatment in mouse and human CFU-Fs (Fig. 7b and Extended Data Fig. 10e,f), consistent with reduced competition from endogenous prenyl donors and restricted RAC1 prenylation. These data identified RAC1 as a required mediator of statin-induced promotion of *Oln* transcription.

We next assessed whether RAC1 is required for statin-induced osteogenic differentiation and bone anabolism. *Rac1/RAC1* knockout abolished atorvastatin-induced osteogenic differentiation in mouse and human CFU-Fs (Fig. 7c and Extended Data Fig. 10g). In ovariectomized mice with conditional *Rac1* deletion in LEPR^+^ cells, atorvastatin neither increased bone mass nor reduced bone marrow adipocyte number (Fig. 7d,e and Extended Data Fig. 10h–k). These results established that RAC1 is required for the statin-induced promotion of *Oln* transcription, osteogenic differentiation of LEPR^+^ cells and bone anabolism in mice.

### Statins restrict RAC1 prenylation to promote NF-κB-mediated *Oln* transcription

In its GTP-bound active form, RAC1 binds to and activates effector proteins to propagate downstream signaling^39^. Blocking RAC1 prenylation increases its activation^40^, and activated RAC1 can promote NF-κB-dependent transcription through RAC1-mediated IκBα degradation^40–42^. We therefore tested whether statin-mediated restriction of RAC1 prenylation increases RAC1 activation. In mouse and human CFU-Fs, atorvastatin significantly increased GTP-bound RAC1 compared with vehicle-treated controls, as assessed by RAC1 activation assays^43^ (Fig. 7f,g). Together with our finding that atorvastatin reduces RAC1 prenylation, these results indicate that statins increase RAC1 activation by limiting its prenylation.

We next examined whether the statin-induced increase in RAC1 activation promotes NF-κB recruitment to the *Oln*/*OLN* locus. A CUT&Tag analysis of mouse and human CFU-Fs revealed that atorvastatin treatment led to increased NF-κB occupancy at the *Oln*/*OLN* locus compared with vehicle-treated controls (Fig. 7h,i). Consistent with these findings, chromatin immunoprecipitation coupled with quantitative PCR (ChIP–qPCR) analyses further revealed significantly increased NF-κB binding to the *Oln*/*OLN* locus following atorvastatin treatment in both mouse and human CFU-Fs (Extended Data Fig. 10l,m). Thus, by dissecting the metabolic consequences of statin-mediated inhibition of the mevalonate pathway, we define a regulatory pathway in which statin-mediated restriction of RAC1 prenylation increases GTP loading of RAC1 and enables NF-κB recruitment to the *Oln*/*OLN* locus, thereby promoting *Oln*/*OLN* transcription and ultimately driving osteogenic differentiation and bone anabolism.

## Discussion

Our results show that statin use is associated with an attenuated rate of annual BMD loss in postmenopausal women and that statins promote bone anabolism in aged female and ovariectomized mice by enhancing bone formation rather than altering bone resorption. We identify LEPR^+^ skeletal stem/progenitor cells as the cellular site at which endocrine state and statin treatment are integrated to regulate osteogenic differentiation, repositioning a major component of E2–ERα action to an earlier stage of the osteogenic trajectory than previously appreciated. These distinct inputs converge on *Oln*/OLN: E2–ERα signaling promotes *Oln* transcription as part of the E2-induced bone anabolic response, whereas statins restrict RAC1 prenylation, increase RAC1 activation and enable NF-κB recruitment to the *Oln* locus to promote *Oln* transcription (Fig. 7j). Collectively, these findings define a stem/progenitor-based mechanism through which estrogen and statins promote bone anabolism and identify OLN as a shared mediator of osteogenic differentiation during estrogen deficiency.

Estrogen deficiency in women is characterized by reduced bone formation, deterioration of the trabecular and cortical microarchitecture, and increased fracture susceptibility across natural and induced hypoestrogenic states^1^. Our findings indicate that skeletal responses to statins are context dependent: statin use was associated with attenuated BMD loss in estrogen-deficient women, and statins elicited a bone anabolic effect in aged female and ovariectomized mice. These observations support the idea that estrogen deficiency and stem/progenitor cell responsiveness contribute to variability in skeletal adaptation to widely prescribed statin drugs. This context dependence may help explain why clinical studies of statins and skeletal outcomes have yielded variable results, with observational associations in some cohorts but no consistent fracture-preventive effect in randomized or meta-analytic studies^44,45^. The absence of a detectable statin response in aged males, young adult mice and orchiectomized males indicates that atorvastatin-induced bone anabolism is not a uniform skeletal response to mevalonate-pathway inhibition. Rather, estrogen deficiency in females appears to provide a permissive endocrine context for this response, although sex-dependent differences in LEPR^+^ cells or OLN regulation may also contribute.

Estrogen signaling through ERα has traditionally been understood to act at the level of osteoblasts and osteocytes^17,22^. Our findings indicate that E2–ERα signaling operates earlier in the osteogenic trajectory, specifically within LEPR^+^ skeletal stem/progenitor cells. By distinguishing osteoblast/osteocyte-restricted and LEPR^+^ cell-specific *Esr1* deletion, we show that the E2-induced bone anabolism was reduced by *Esr1* deletion in LEPR^+^ cells but not by osteoblast/osteocyte-restricted *Esr1* deletion. Moreover, *Esr1* deletion in LEPR^+^ cells accelerated bone loss during adulthood, whereas osteoblast/osteocyte-restricted *Esr1* deletion did not reduce adult bone mass. This repositioning refines the developmental window in which E2–ERα signaling is integrated into LEPR^+^ cells and motivates approaches that can distinguish effects acting at early stem/progenitor stages from those operating in osteoblasts and osteocytes at later stages of differentiation^16,24^. LEPR^+^ cells are heterogeneous^30,33–35^, and our current genetic approaches do not determine whether estrogen and statins act broadly across this population or preferentially through a more restricted subset. Because OLN is secreted, the OLN-producing and OLN-responsive cells may not be identical. Future subset-specific genetic approaches targeting distinct LEPR^+^ subsets will be needed to test whether particular LEPR^+^ subpopulations preferentially mediate these responses.

Statins inhibit the mevalonate pathway and reduce the availability of isoprenoid substrates required for protein prenylation, a modification essential for the localization and signaling of many small GTPases^37^. In LEPR^+^ skeletal stem/progenitor cells, our data show that statin treatment restricts RAC1 prenylation, increases the abundance of GTP-bound RAC1 and promotes osteogenic differentiation by enabling NF-κB recruitment to the *Oln* locus. These findings provide an example of how prenylation-sensitive signaling can be interpreted at the level of a defined stem/progenitor cell population to drive tissue-level anabolic outcomes.

These observations raise several questions for future study, including whether other progenitor populations exhibit similar sensitivity to statin-mediated restriction of prenylation and how cell-type-specific thresholds for small GTPase activity shape differentiation outcomes. Statins have also been reported to inhibit prenylation in nonskeletal lineages, including endothelial progenitors^46^ and immune cells^47–49^, suggesting that the consequences of impaired prenylation may be highly dependent on cell type. More broadly, given the widespread clinical use of statins in older populations, understanding how impaired prenylation is interpreted by distinct stem/progenitor states will be important for elucidating tissue-specific consequences of long-term statin use.

## Methods

### Human samples

Human sample procurement and phenotyping were conducted in accordance with the Declaration of Helsinki and were approved by the Ethics Committees of Beijing Jishuitan Hospital (K2025-009-00), Suzhou Kowloon Hospital (KY-2021-024) and the National Institute of Biological Sciences, Beijing (IRB25022001). For the retrospective observational analyses of DXA data and pharmacy dispensing and administration records, the requirement for informed consent was waived by the same ethics committees. A total of 169 postmenopausal women aged 55–65 years at the first DXA scan used for analysis were enrolled (80 from Beijing Jishuitan Hospital and 89 from Kowloon Hospital). All participants had been amenorrheic for at least 3 years and had undergone at least two lumbar spine DXA examinations separated by a minimum interval of 6 months. The retrospective DXA cohort data were collected from 2011 to 2026 at Beijing Jishuitan Hospital and from 2011 to 2025 at Kowloon Hospital. The maximum interval between paired DXA scans included in the analysis was 78 months in the Beijing Jishuitan Hospital cohort and 53 months in the Kowloon Hospital cohort. Age at menopause, years since menopause and the interval between paired DXA scans were obtained from clinical records and are reported together with other baseline variables in Extended Data Fig. 1a,b. Participants were stratified into three groups based on pharmacy dispensing and administration records: (i) statin users, defined as individuals who received continuous statin therapy throughout the interval between the paired DXA scans used to compute annualized BMD change; (ii) evolocumab users, defined as individuals who received subcutaneous evolocumab, a PCSK9 inhibitor, throughout the interval between the paired DXA scans used to compute annualized lumbar spine BMD change; and (iii) control participants, who had no record of lipid-lowering therapy during the observation window. The mean and median durations of statin therapy and evolocumab therapy during the DXA observation interval are reported in Extended Data Fig. 1a,b. Exclusion criteria included: (i) use within the preceding 2 years of bone-active medications, including bisphosphonates, denosumab, teriparatide, romosozumab, selective estrogen receptor modulators or systemic glucocorticoids; (ii) metabolic bone disease (including primary hyperparathyroidism or Paget’s disease), inflammatory rheumatic disease, chronic kidney disease (estimated glomerular filtration rate < 45 ml min^−1^ 1.73 m^−2^), any malignancy or uncontrolled thyroid disease; and (iii) incomplete or technically inadequate DXA measurements. DXA scans of the lumbar spine (L1–L4) were acquired using a GE Lunar iDXA system (GE Healthcare) with standardized positioning and quality-control procedures according to the manufacturer’s guidelines. Absolute annualized lumbar spine BMD change was calculated as the difference between two serial DXA measurements divided by the interval between scans in years and expressed as g cm^−2^ per year. Annual percentage BMD change was calculated as absolute annualized BMD change divided by baseline lumbar spine BMD and multiplied by 100.

Written informed consent was obtained from all participants who provided plasma or bone marrow samples; this component of the study was approved by the ethics committees (K2022-168-03 and KY-2021-024). Plasma samples used for circulating OLN measurements were obtained from a separate cohort of postmenopausal participants recruited from the same hospitals. This plasma cohort used the same general inclusion and exclusion criteria as the retrospective DXA cohort, except that plasma availability was required. Circulating proteins in fasting plasma samples were quantified using a validated Bio-Plex Pro Human Cytokine 48-plex Screening Panel (Bio-Rad, 12007283). Human bone marrow samples were obtained from consenting adults undergoing total hip replacement surgery. Samples were enzymatically dissociated immediately after collection to generate single-cell suspensions. Unfractionated bone marrow cells were plated directly under the CFU-F culture conditions described below; where indicated, LEPR^+^ cells were prospectively isolated by flow cytometry for downstream analyses.

### Mice

All mouse experiments were conducted in compliance with institutional and national ethical guidelines and were approved by the Institutional Animal Care and Use Committee of the National Institute of Biological Sciences, Beijing (NIBS2026M0042). All mice were maintained on a C57BL/6J genetic background. The following mouse lines were used: *Esr1*^fl^ (Jackson Laboratory, JAX: 032173)^50^, *Oln*^−/−^ (ref. ^29^), *Rac1*^fl^ (GemPharmatech: T009642), *Rosa26*^tdTomato^ (*Ai14*, JAX: 007914)^51^, *Lepr*^BFP^ (JAX: 040198)^52^, *Rosa26*^Cas9^ (JAX: 026179)^53^, Col1a1*2.3-eGFP (JAX: 013134)^54^, *Col1a1*-CreER (JAX: 016241)^28^ and *CMV*-Cre (JAX: 006054)^55^.

The Tet-On *Lepr*-Cre transgenic line was generated by pronuclear injection of the bacterial artificial chromosome clone RP23-184F13, in which the endogenous *Lepr* locus was modified to contain an insulator–*Lepr* promoter–*rtTA*–*polyA*–*WPRE*–*iCre*–*TRE3G*–insulator cassette, into fertilized C57BL/6J oocytes. To generate *Esr1*^GFP^ reporter mice, *Cas9* mRNA, a single-guide RNA (sgRNA) targeting the *Esr1* locus, and a donor plasmid encoding a tandem GFP reporter cassette (*EmGFP*–*T2A*–*TagGFP2*–*P2A*–*hrGFP*) that was inserted downstream of an alternative ATG in exon 3 of the *Esr1* locus were co-microinjected into C57BL/6J zygotes. To generate *Hipp11*^Oln−URE−LSL−mScarlet^ reporter mice, *Cas9* mRNA, a sgRNA targeting the *Hipp11* locus and a donor plasmid encoding an Oln–URE–LSL–mScarlet cassette were co-microinjected into C57BL/6J zygotes. To generate *Oln*^NanoLuc^ reporter mice, *Cas9* mRNA, a sgRNA targeting the *Oln* locus and a donor plasmid encoding NanoLuc luciferase^56^ (NanoLuc) that was inserted immediately downstream of the endogenous start codon were co-microinjected into C57BL/6J zygotes. For *Esr1*^GFP^, *Hipp11*^Oln−URE−LSL−mScarlet^ and *Oln*^NanoLuc^ lines, correctly targeted founders were identified by restriction fragment length polymorphism analysis and Southern blotting using 5′ and WPRE (woodchuck hepatitis virus posttranscriptional regulatory element) probes, followed by Sanger sequencing of the targeted alleles. Founder mice were backcrossed to wild-type C57BL/6J mice for at least three generations before use in experiments.

### Primer sequences

Genotyping primers for *Esr1*^GFP/+^ mice were 5′-GAG TTG GGT CAC CTG TGT TCT GCA G-3′, 5′-CTC TCC ATG GGC ATC TTG AGC TGC-3′ and 5′-ACG AGT CGG ATC TCC CTT TGG-3′. Genotyping primers for Tet-On *Lepr*-Cre mice were 5′-GAG TAA CTA TGG ATA CCA GGA AAG AGA AGA AG-3′, 5′-GGT ACC TGA CAT CGA TTC CAT GTC TAG AC-3′, 5′-ATT ATG TTG AAT GAC ACA ACT CTG TCC TAG-3′ and 5′-AGT TCC AGC TCA GTG CTT CAA GTT C-3′. Genotyping primers for *Hipp11*^Oln−URE−LSL−mScarlet^ mice were 5′-CAA GCT AAT TTG ACC AGT CAT TGG A-3′, 5′-GCA GAC ACC CAG GAT AAG TG-3′ and 5′-TCT AGA TGG CCA GAT CTA TTT AAA CGG-3′. Genotyping primers for *Oln*^NanoLuc/+^ mice were 5′-TGC CCA CCA GAC TGG GAC ACT T-3′, 5′-ACC CAT CTG AAC ACT TCA TCT CAA GCC-3′ and 5′-AAT AGT CGA TCA TGT TCG GCG TAA CC-3′. Primers used for qPCR were as follows: *Oln*, 5′-AGG TCC TGG GAG GGA GTG-3′ and 5′-GGG CCT CCT GGA GAT TCT T-3′; *Esr1*, 5′-TCT GCC AAG GAG ACT CGC TAC T-3′ and 5′-GGT GCA TTG GTT TGT AGC TGG AC-3′; *Actb*, 5′-GCT CTT TTC CAG CCT TCC TT-3′ and 5′-CTT CTG CAT CCT GTC AGC AA-3′.

### Gonadectomy procedures

Eight-week-old female mice underwent sham surgery or bilateral ovariectomy, and age-matched males underwent sham surgery or bilateral ORX, under ketamine (100 mg per kg body weight) and xylazine (10 mg per kg body weight) anesthesia. Ovaries were removed through bilateral flank incisions. Testes were removed through scrotal incisions after ligation of the spermatic cords and associated vessels. Sham-operated mice underwent the corresponding procedure without gonad removal. Incisions were closed with surgical clips for ovariectomy or absorbable sutures for ORX. Mice recovered for 4 weeks before pharmacological treatment.

### Pharmacological treatments and genetic induction

Treatments after gonadectomy began 4 weeks after ovariectomy, ORX or sham surgery. Tet-On *Lepr*-Cre mice received doxycycline from 2 to 3 months of age for 4 weeks in light-protected drinking water (2 mg ml^−1^; MedChemExpress, HY-N0565B) containing 5% sucrose, replaced every other day. CreER recombination was induced at 2 months by intraperitoneal injection of tamoxifen (1 mg per mouse per day; Sigma-Aldrich, T5648) for 5 consecutive days. Subsequent treatments or analyses were performed at the indicated times. E2 (10 µg per kg per day; Sigma-Aldrich, E2758) was delivered subcutaneously for 4 weeks using Alzet 1004 osmotic pumps implanted at 2 months of age or 4 weeks after ovariectomy or sham surgery. Atorvastatin (10 mg per kg per day; MedChemExpress, HY-B0589) was administered by daily oral gavage for 8 weeks beginning at 2 or 18 months of age or 4 weeks after ovariectomy, ORX or sham surgery. Evolocumab (10 mg per kg per week; MedChemExpress, HY-P9930) was administered subcutaneously once weekly for 8 weeks beginning at 2 months of age or 4 weeks after ovariectomy or sham surgery.

### CFU-F and in vitro differentiation assays

Freshly isolated and enzymatically dissociated mouse or human bone marrow cells were plated at clonal density (5 × 10^6^ cells per 10-cm dish) in low-glucose DMEM (Gibco) containing 20% fetal bovine serum (Gemini), 10 μM Y-27632 (Selleck) and 1% penicillin–streptomycin (Gibco). Cultures were maintained at 37 °C in gas-tight chambers (Billups-Rothenberg) under 1% O_2_, 6% CO_2_ and a balance of N_2_. Non-adherent cells were removed on day 2, cultures were flushed daily with the hypoxic gas mixture and medium was changed every 3 days. On day 8, colonies were fixed, stained with 0.1% Toluidine blue in 4% formalin and counted.

For differentiation, pooled passage-1 CFU-F cultures were replated at 25,000 cells cm^−2^. On day 2, medium was replaced with α-MEM (Gibco) containing 20% fetal bovine serum, 1% penicillin–streptomycin, 10 nM dexamethasone, 50 μg ml^−1^ L-ascorbic acid-2-phosphate and 3 mM β-glycerophosphate. Atorvastatin (10 μM), E2 (100 nM), DHT (100 nM) or vehicle was added, and medium was replaced every 3 days for up to 42 days. Osteogenic differentiation was assessed by Alizarin Red S staining. Stain was extracted with 10% cetylpyridinium chloride in 10 mM sodium phosphate buffer (pH 7.0) for 10 min, and absorbance was measured at 562 nm.

### Luciferase assay

CFU-Fs from *Oln*^NanoLuc/+^ mice were replated into 384-well plates under osteogenic conditions. For pharmacological perturbation, cells were treated for 72 h before NanoLuc activity was measured using the Nano-Glo Luciferase Assay System (Promega, N1130) on a Spark plate reader (TECAN). Compounds were used at 10 µM unless otherwise indicated. For genetic screening, three sgRNAs per target were selected as described^57^ and cloned into AAV-BI vectors produced in HEK293T cells (China Center for Type Culture Collection, GDC0187) by plasmid transfection and purified by polyethylene glycol precipitation and buffer exchange. CFU-Fs derived from *Oln*^NanoLuc/+^; *Rosa26*^Cas9/+^ mice were transduced with 1 × 10^6^ viral genomes per cell. After 72 h, cells were split into vehicle-treated and atorvastatin-treated cultures, and luciferase activity was measured 3 days later. For pharmacological perturbation experiments, luminescence values were normalized on a per-plate basis using *z*-score normalization (*z* = (*x* − *μ*)/*σ*), where *x* represents the luminescence value of an individual well, *μ* is the mean luminescence of all wells on the plate, and *σ* is the corresponding standard deviation. For genetic screening, luminescence values were first normalized to vehicle-treated control sgRNA wells on the same plate. Basal effects of each knockout were assessed in vehicle-treated wells. Atorvastatin responses were calculated for each knockout as the fold change in luminescence (atorvastatin/vehicle) and were normalized to the fold change observed in control sgRNA wells. For each gene, values were averaged across the three sgRNAs.

### Metabolic labeling and click-chemistry detection of prenylated proteins

Metabolic labeling of prenylated proteins was performed in both mouse and human CFU-Fs. Cells were pretreated with either vehicle or 10 μM atorvastatin for 24 h. The medium was then replaced with fresh medium containing 50 μM C15AlkOPP (TargetMol, T30677L), with vehicle or atorvastatin as indicated, and metabolic labeling was carried out for 48 h. Following labeling, cells were lysed, and 100–150 μg of total protein lysate was subjected to a click-chemistry reaction. Proteins were separated by SDS–PAGE, and in-gel fluorescence was visualized using an iBright FL1500 imaging system.

### CUT&Tag and ChIP–qPCR

For ERα CUT&Tag, mouse and human CFU-Fs were cultured in phenol red-free medium with charcoal-stripped serum and treated with vehicle or E2 (100 nM) for 1 h. For NF-κB CUT&Tag, cells received vehicle or atorvastatin (10 μM) for 1 h. CUT&Tag was performed using the Hyperactive Universal CUT&Tag Assay kit (Vazyme, TD904) with anti-ERα (Santa Cruz, sc-8002; EpiCypher, 13-2011) or anti-NF-κB p65 (Cell Signaling Technology, 8242; Abcam, ab218533). Libraries were prepared following tagmentation, sequenced and aligned to GRCh38 or GRCm39, after which peaks were called with MACS. ChIP–qPCR used the same antibodies and locus-specific primers. Primer sequences were as follows: human ERα and NF-κB p65 binding sites, 5′-AGA TGG TTC TGA GCG GGT TC-3′ and 5′-TTC CAC TTT CCA GAG CTC GC-3′; mouse ERα and NF-κB p65 binding sites, 5′-GCC TGC TTG TGG GGA TAT GT-3′ and 5′-TGT CAA CCC TTC TCC TCC CT-3′.

### Flow cytometry

Bone marrow was flushed from long bones with Ca^2+^-free and Mg^2+^-free Hanks’ balanced salt solution (Beyotime, C0218) containing 2% fetal bovine serum (Gemini, 900-108) and enzymatically dissociated. For Col1a1*2.3-eGFP^+^ cell analyses, crushed bone fragments were enzymatically dissociated to recover bone-associated osteoblasts. LEPR^+^ cells were defined as LEPR^+^CD45^−^TER119^−^CD31^−^ using anti-LEPR (Abcam, EPR28293-79), anti-CD45 (Invitrogen, 30-F11), anti-CD31 (Invitrogen, 390) and anti-TER119 (BioLegend, TER119). Endothelial cells were defined as VE-cadherin^+^CD45^−^TER119^−^ using anti-VE-cadherin (Invitrogen, BV13), anti-CD45 and anti-TER119. The VE-cadherin antibody was administered intravenously at 10 µg per mouse and analyzed 10 min after injection, as described previously^58^. Col1a1*2.3-eGFP^+^ osteoblasts were analyzed after excluding CD45^+^, CD31^+^ and TER119^+^ cells. Antibodies were used at a 1:200 dilution. Dead cells were excluded with DAPI or propidium iodide. Data were acquired on BD FACSAria Fusion or FACSAria III instruments.

### Immunostaining of bone sections

Femurs and tibiae were fixed in 4% paraformaldehyde (Solarbio, P1110) for 6 h at 4 °C, washed and decalcified in 0.5 M EDTA (pH 8.0) at 4 °C. Samples were cryoprotected in 30% sucrose (Macklin, S818046) for 12 h, embedded in OCT (Sakura, 4583) and stored at −80 °C. Femurs were sectioned longitudinally at 50 μm using a CryoJane tape-transfer system. Sections were blocked in PBS containing 5% donkey serum (Jackson ImmunoResearch, 017-000-121) for 1 h, incubated with primary antibodies overnight at 4 °C and then with fluorescent secondary antibodies. Bone marrow adipocytes were identified as perilipin^+^ cells by immunofluorescence staining of bone sections and counted throughout the metaphyseal bone marrow. For bone hemisections, femurs were longitudinally bisected and processed for whole-mount immunostaining and deep confocal imaging as described^52^. Antibodies used for immunostaining included: goat anti-Discosoma tdTomato (LSBio, used at a 1:250 dilution), chicken anti-GFP (Aves Labs, used at a 1:250 dilution), goat anti-endomucin (R&D Systems, used at a 1:250 dilution), rabbit anti-perilipin-1 (Cell Signaling Technology, used at a 1:1,000 dilution), rabbit anti-tRFP/BFP (Evrogen, used at a 1:200 dilution), Alexa Fluor 555-conjugated donkey anti-goat IgG (Invitrogen, used at a 1:500 dilution), Alexa Fluor 647-conjugated AffiniPure F(ab′)_2_ donkey anti-rabbit IgG (Jackson ImmunoResearch, used at a 1:250 dilution), Alexa Fluor 488-conjugated AffiniPure F(ab′)_2_ donkey anti-chicken IgG (Jackson ImmunoResearch, used at a 1:250 dilution).

### MicroCT analysis

Femurs were fixed overnight in 4% paraformaldehyde, stored in 70% ethanol and scanned using a NEMO NMC-200 system (PINGSENG Healthcare) at 8-μm isotropic resolution, 90 kV and 0.04 mA. Reconstructed images were filtered with a three-dimensional Gaussian filter (*σ* = 1.0), and mineralized bone was segmented using a global threshold of 700–2,500. The trabecular region comprised 100 consecutive slices in the distal metaphysis, beginning immediately proximal to the growth plate where the epiphyseal cap was no longer visible. Contours were traced several voxels away from the endocortical surface to exclude cortical bone. Cortical parameters were measured over 100 consecutive mid-diaphyseal slices. Standard trabecular and cortical parameters were calculated.

### Calcein and Alizarin Red S double labeling

Mice were injected intraperitoneally with calcein (20 mg kg^−^^1^ body weight; MCE) and Alizarin Red S (30 mg per kg body weight; Sigma) twice at an interval of 9 days and were euthanized 4 days after the second injection. Femurs were dissected and fixed overnight in 4% paraformaldehyde at 4 °C, followed by dehydration in 30% sucrose at 4 °C for 24 h before embedding in OCT compound. Embedded bones were sectioned, dehydrated, optically cleared and imaged as previously described^52^. MAR and BFR were quantified as previously described^59^.

### RNA-seq

Sorted cells were collected into RLT buffer, and RNA was purified using the RNeasy Plus Micro kit (Qiagen, 74034) and assessed with a Pico Bioanalyzer. cDNA was generated using SMART-Seq v4 (Takara/Clontech, 634891), and libraries were prepared with the TruePrep DNA Library Prep kit V2 (Vazyme, TD503), quantified by Qubit and assessed using a Fragment Analyzer. Libraries were sequenced on an Illumina NovaSeq X Plus. Reads were evaluated with FastQC v0.12.1, trimmed with Cutadapt v5.0 and aligned to GRCh38 or GRCm39 using STAR v2.7.11b. Gene counts were generated with featureCounts v2.0.4, and differential expression was analyzed using DESeq2 v1.38.3 in R v4.2.3. For reanalysis of published single-cell RNA-seq data^33^, processed expression matrices and cell annotations were used to visualize *Esr1*, *Oln*, *Sp7* and *Bglap* across annotated bone marrow LEPR^+^ subsets.

### Statistical methods

For animal studies, independent experiments corresponded to separate cohorts of mice generated, treated and analyzed at different times using the same experimental protocol. Data from all animals across independent experiments were pooled for statistical analysis. Mice were allocated to experiments randomly, and samples were processed in an arbitrary order. However, formal randomization techniques were not used. Before assessing statistical significance among treatments, data were tested for normality and homogeneity of variance. Normality was assessed using the Shapiro–Wilk test when 3 ≤ *n* < 20, or the D’Agostino–Pearson omnibus test when *n* ≥ 20. Homogeneity of variance was assessed using *F*-tests for comparisons between two groups or Brown–Forsythe tests for comparisons involving more than two groups. When data significantly deviated from normality or showed unequal variance, log_2_ transformation was applied and tests for normality and variance were repeated. If the transformed data no longer significantly deviated from normality and exhibited equal variance, parametric tests were performed on the transformed data. If the transformed data continued to deviate from normality or homogeneity of variance, nonparametric tests were applied to the non-transformed data. When data or log_2_-transformed data were normally distributed and exhibited equal variance, statistical analyses were performed using Student’s *t*-tests for comparisons between two groups, one-way ANOVA for comparisons among more than two groups or two-way ANOVA when experiments involved two independent variables (for example, genotype and treatment). When data or log_2_-transformed data were normally distributed but exhibited unequal variance, Welch’s *t*-tests were used for two-group comparisons and Welch’s ANOVA for comparisons among more than two groups. When data or log_2_-transformed data were abnormally distributed, statistical analyses were performed using Mann–Whitney tests for two-group comparisons or Kruskal–Wallis tests for comparisons among more than two groups. After ANOVAs, *P* values from multiple comparisons were adjusted using Dunnett’s, Dunnett’s T3, Tukey’s or Šídák’s method or the Benjamini–Hochberg correction. After Kruskal–Wallis tests, multiple comparisons were adjusted using Dunn’s method. After multiple Mann–Whitney tests, multiple comparisons were adjusted using the Benjamini, Krieger and Yekutieli two-stage linear step-up procedure (5% false discovery rate). All statistical analyses were performed using GraphPad Prism v10.4.2 and R v4.2.3 with the tidyverse and broom packages. All statistical tests were two sided. Data are presented as the mean ± s.d. *P < 0.05, **P < 0.01, ***P < 0.001, NS, *P* > 0.05.

### Reporting summary

Further information on research design is available in the Nature Portfolio Reporting Summary linked to this article.

## Supplementary information

Supplementary Information Unprocessed gels or blots for Figs. 3, 6 and 7 and Extended Data Figs. 4, 7 and 10.

Reporting Summary

Peer Review File

## Source data

Source Data Fig. 1 Statistical source data.

Source Data Fig. 2 Statistical source data.

Source Data Fig. 3 Statistical source data.

Source Data Fig. 4 Statistical source data.

Source Data Fig. 5 Statistical source data.

Source Data Fig. 6 Statistical source data.

Source Data Fig. 7 Statistical source data.

Source Data Extended Data Fig. 1 Statistical source data.

Source Data Extended Data Fig. 2 Statistical source data.

Source Data Extended Data Fig. 3 Statistical source data.

Source Data Extended Data Fig. 4 Statistical source data.

Source Data Extended Data Fig. 5 Statistical source data.

Source Data Extended Data Fig. 6 Statistical source data.

Source Data Extended Data Fig. 7 Statistical source data.

Source Data Extended Data Fig. 8 Statistical source data.

Source Data Extended Data Fig. 9 Statistical source data.

Source Data Extended Data Fig. 10 Statistical source data.

## Data Availability

Further information and requests for resources and reagents should be directed to B.S. No original code was generated in this study. Mouse RNA-seq and CUT&Tag raw sequencing data have been deposited in the Genome Sequence Archive (GSA) at the National Genomics Data Center (NGDC; https://ngdc.cncb.ac.cn/gsa/) under accession number CRA036045 (PRJCA054117). Human RNA-seq and CUT&Tag raw sequencing data have been deposited in the Genome Sequence Archive for Human (https://ngdc.cncb.ac.cn/gsa-human/) under accession number HRA015691 (PRJCA054117). Processed data have been deposited in the OMIX database at the NGDC (https://ngdc.cncb.ac.cn/omix/) under accession numbers OMIX013936, OMIX013945, OMIX013951 and OMIX013887. All sequencing data will be publicly available upon publication. Source data are provided with this paper.
