# Supplementary material for: Statins promote bone anabolism in estrogen deficiency by inducing osteogenic differentiation of skeletal stem/progenitor cells
Source: Nat Aging. 2026 Oct 27;6(11):2393–409. doi: 10.1038/s43587-026-01257-1 (PMC13754606; doi:10.1038/s43587-026-01257-1)

nature aging

Supplementary information https://doi.org/10.1038/s43587-026-01257-1

# Statins promote bone anabolism in estrogen deficiency by inducing osteogenic differentiation of skeletal stem/progenitor cells

In the format provided by the authors and unedited

# Unprocessed images in Figure 3

**k**

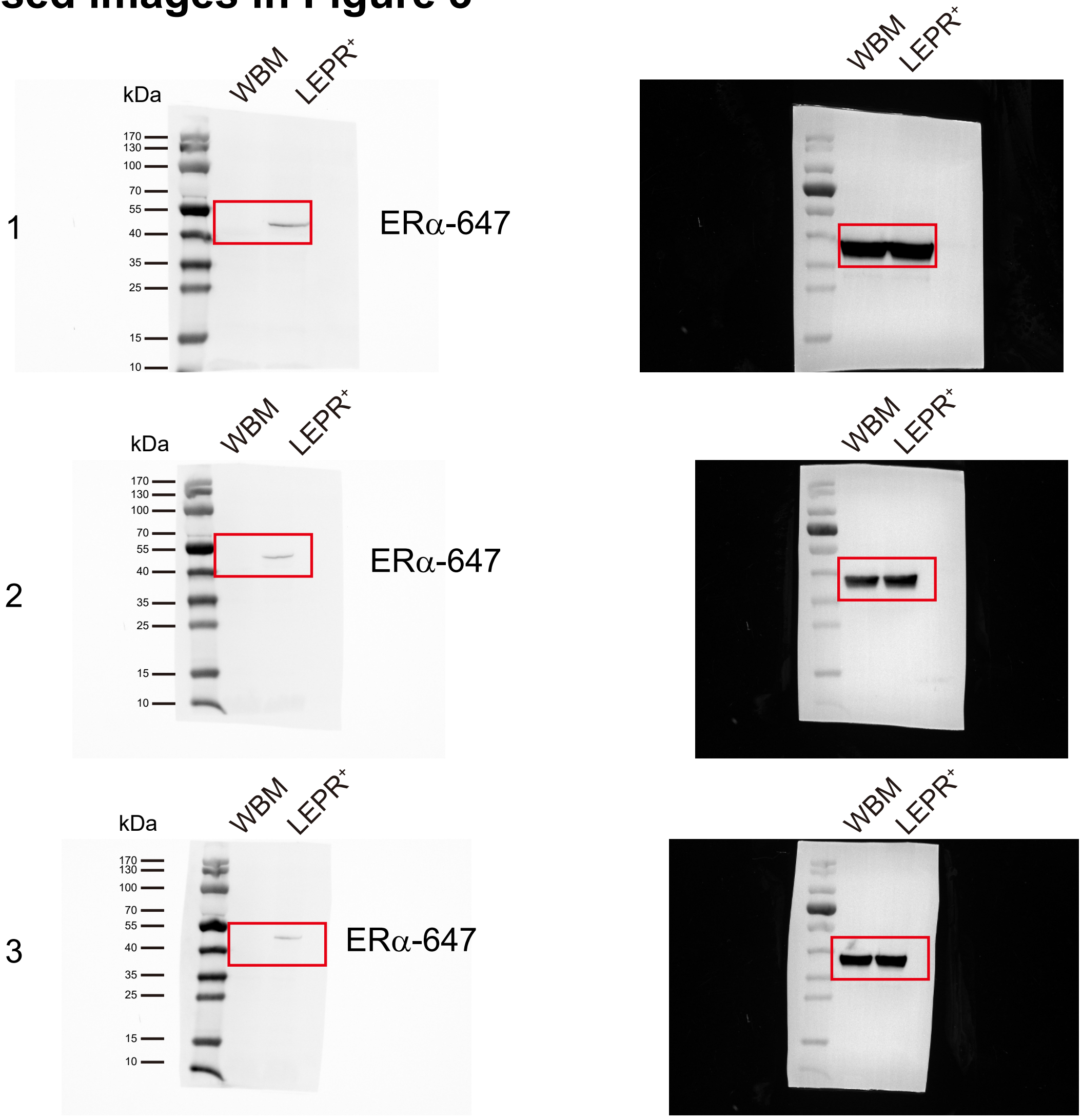


**l**

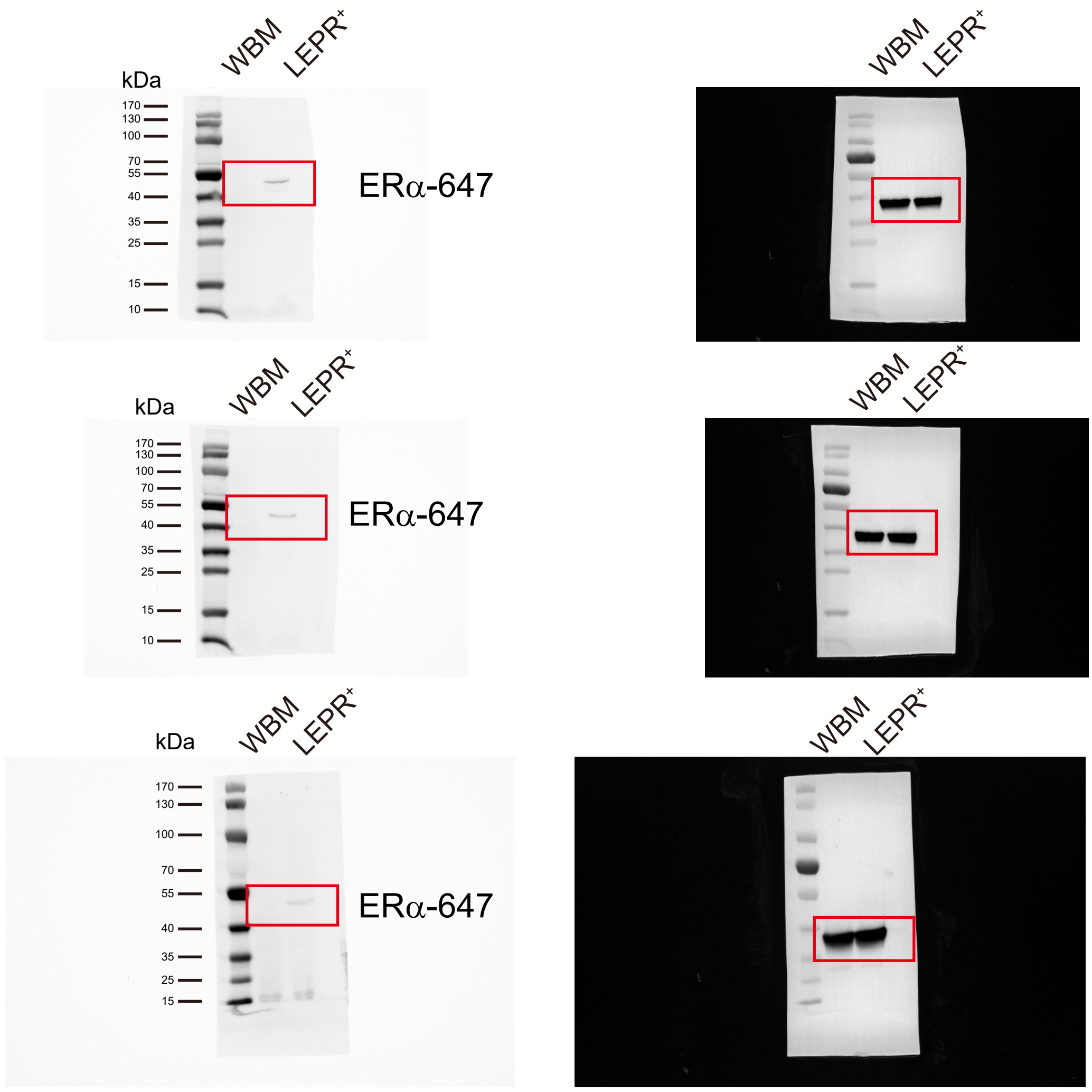

# Unprocessed images in Figure 6

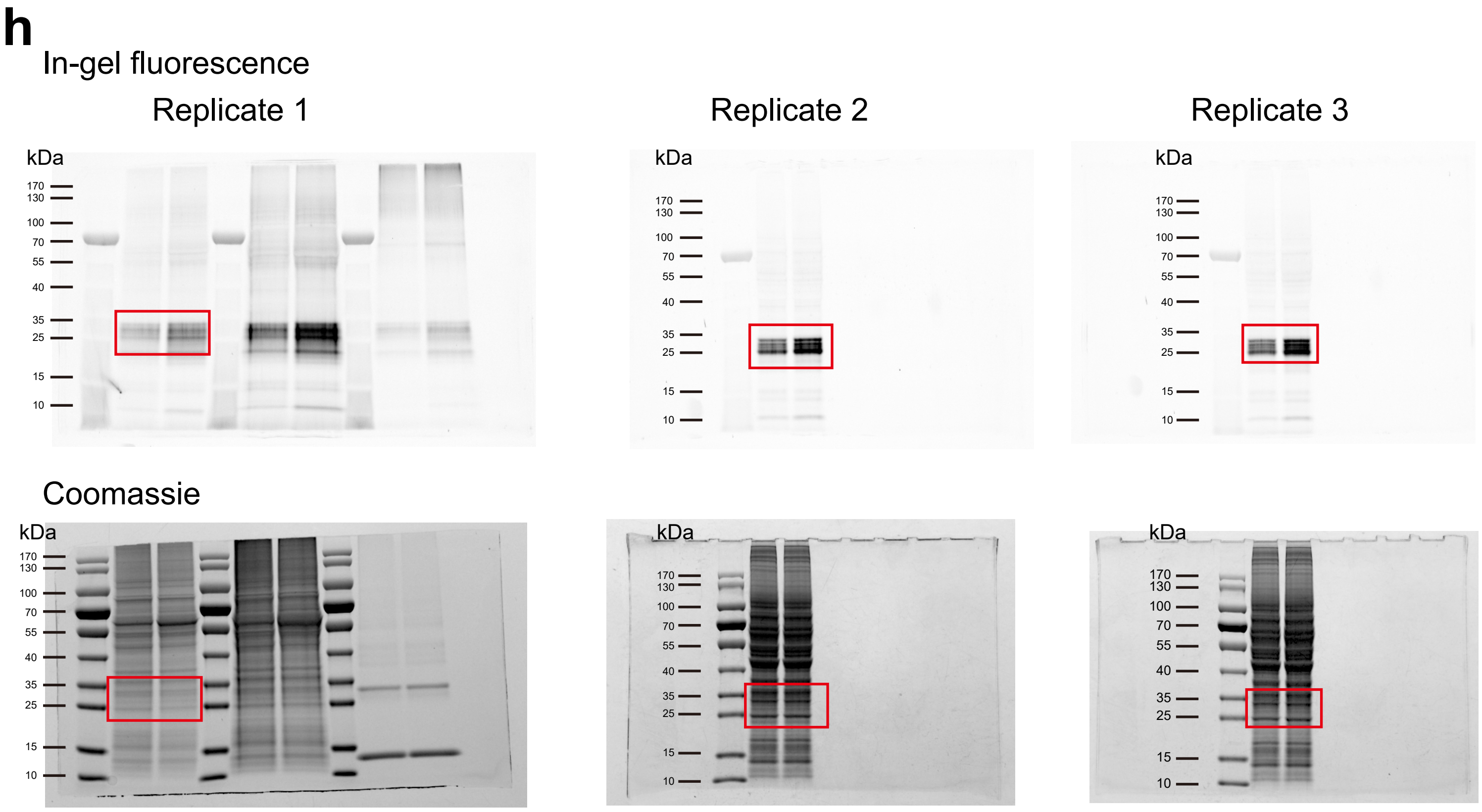


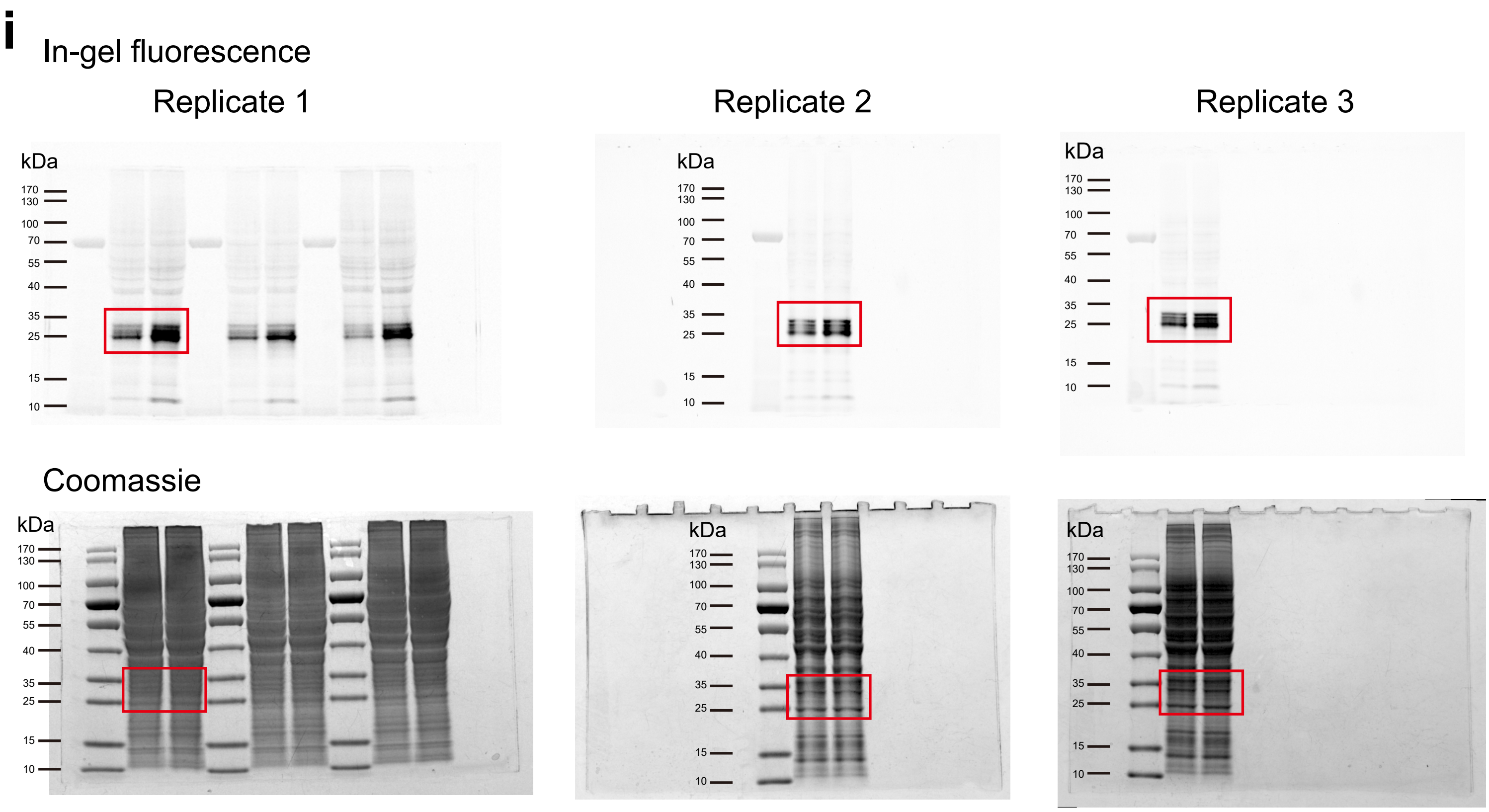

# Unprocessed images in Figure 7

**b** Replicate 1

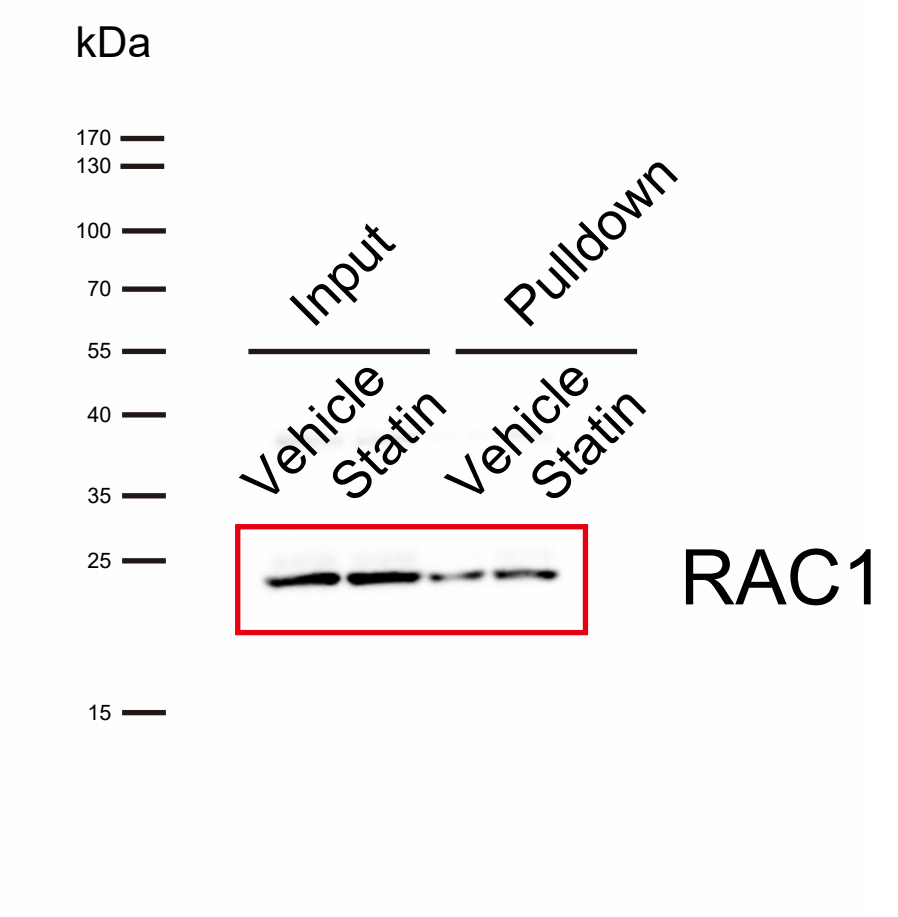


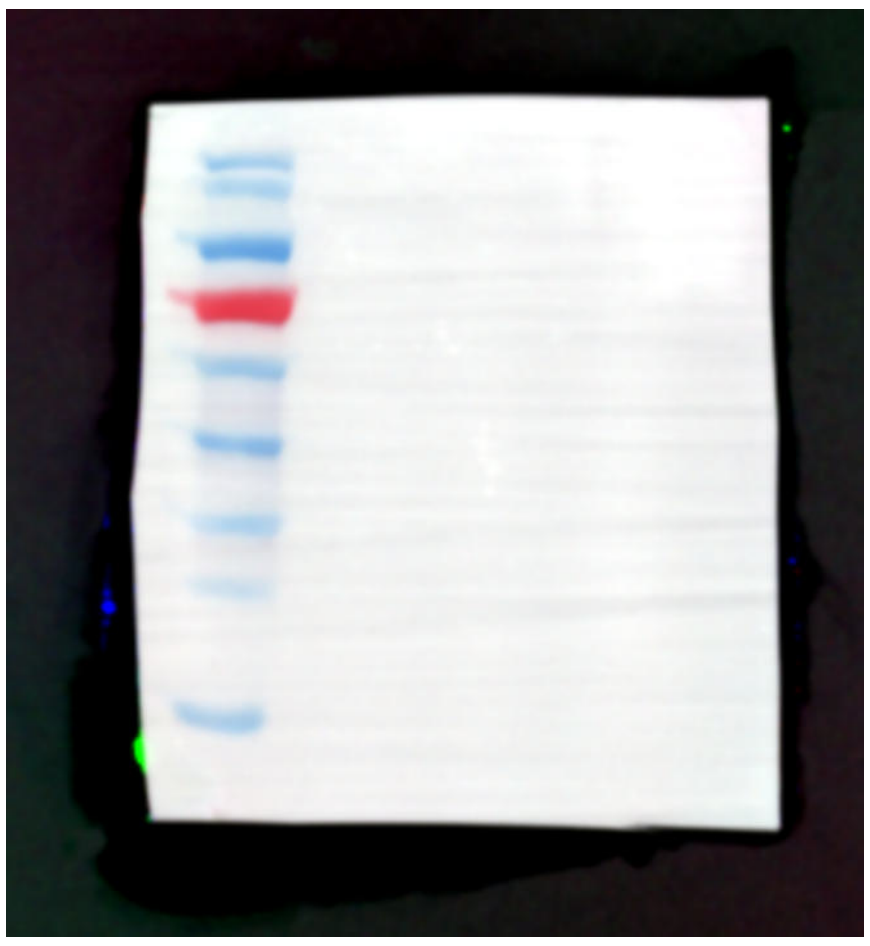

Replicate 2

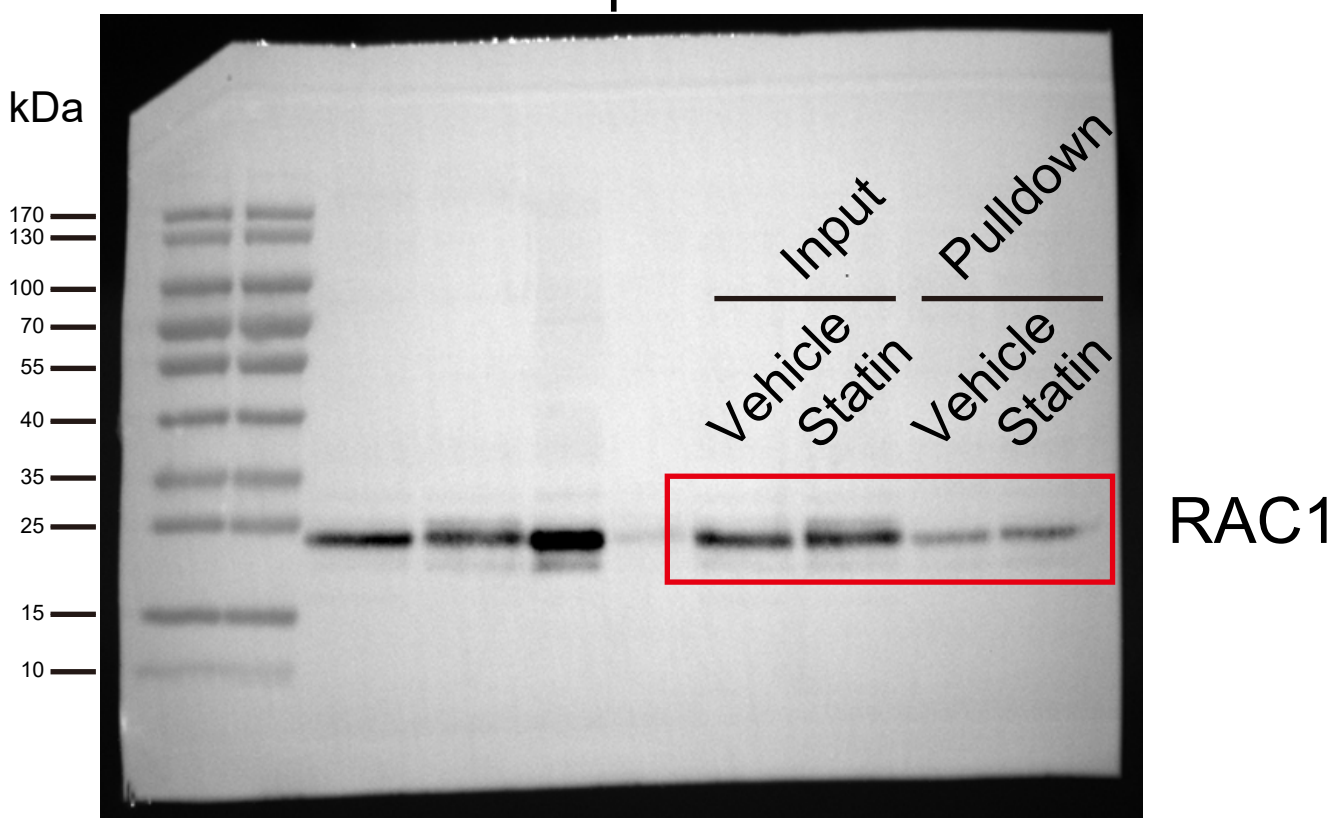


Replicate 3

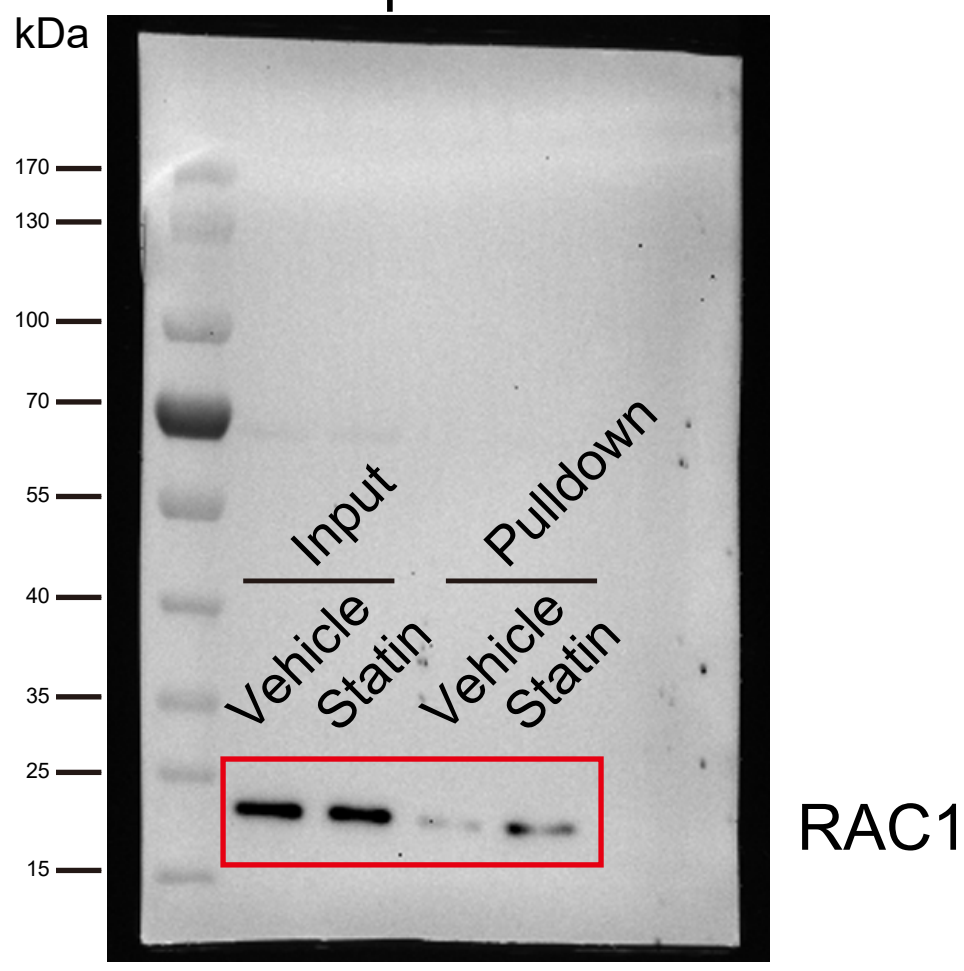

# Unprocessed images in Figure 7

**f**

Replicate 1

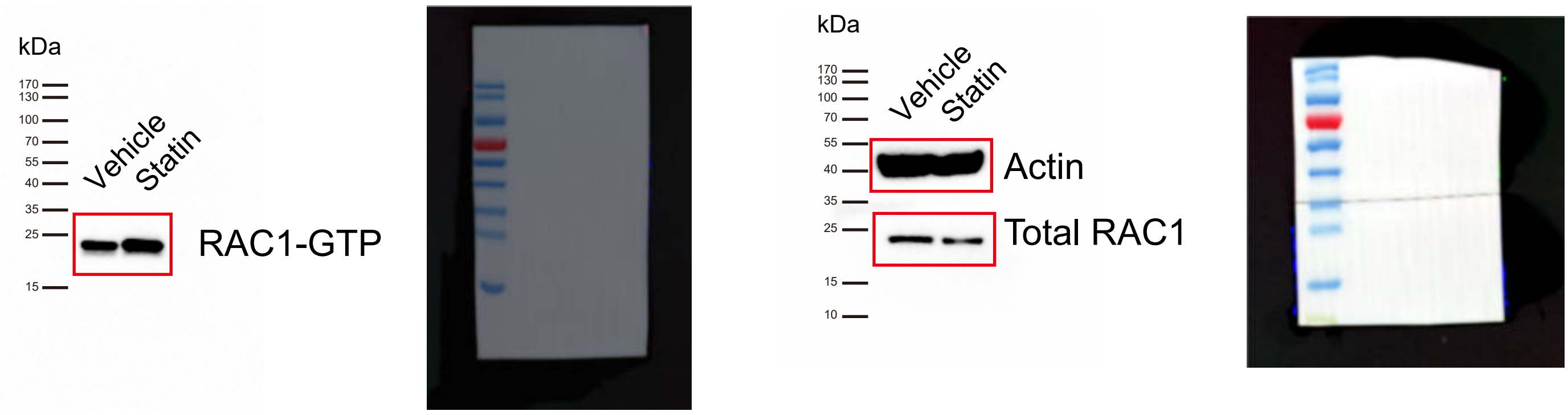


Replicate 2

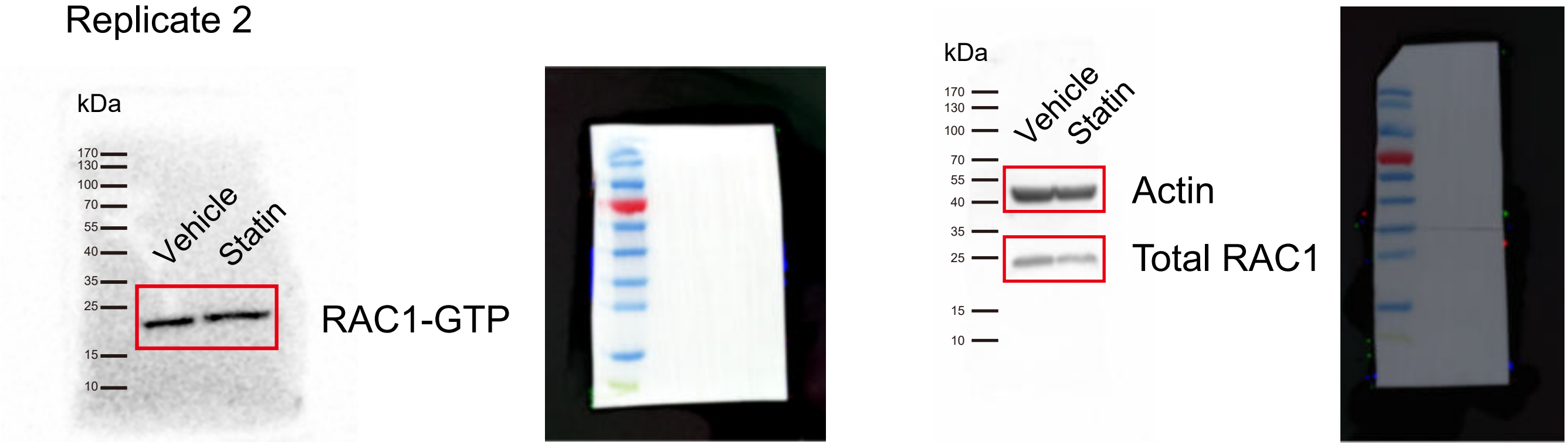


Replicate 3

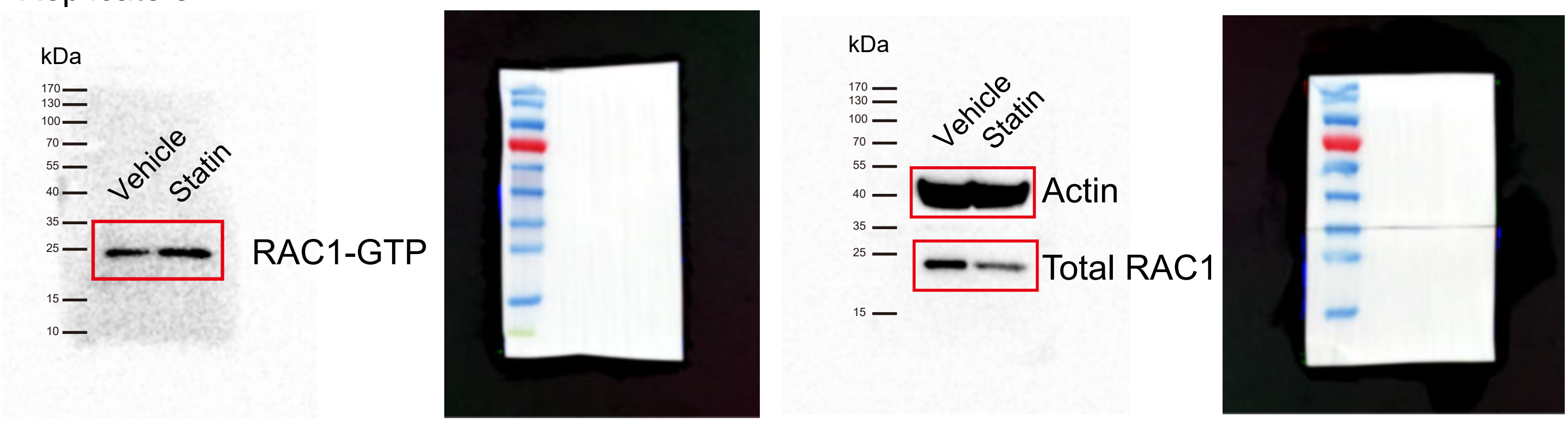

# Unprocessed images in Figure 7

**g**

Replicate 1

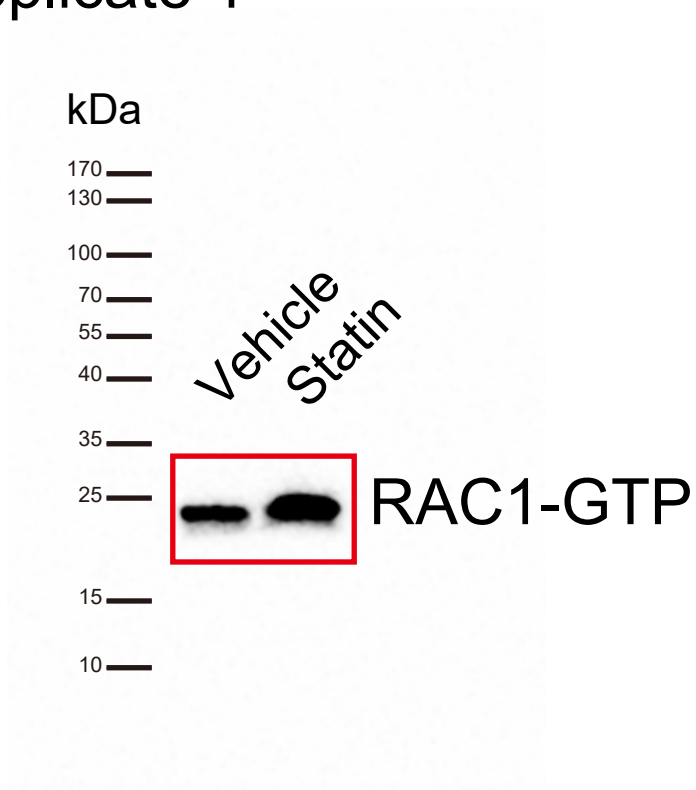


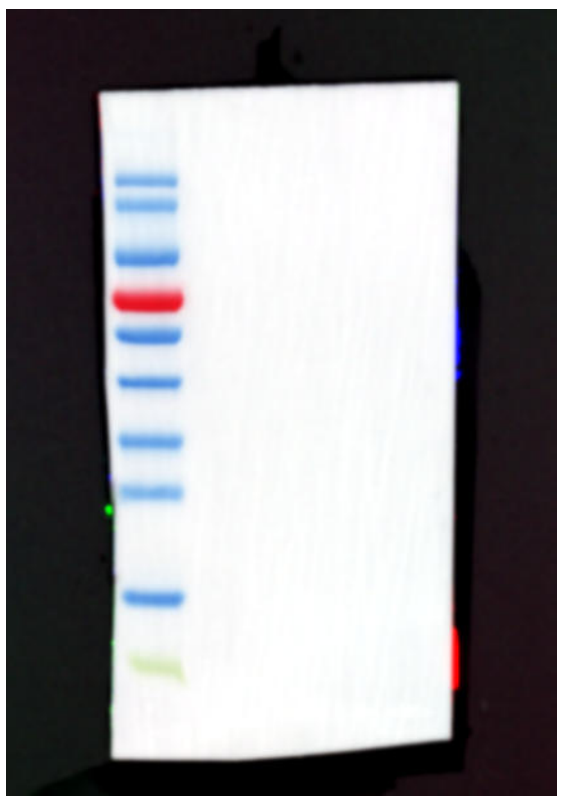

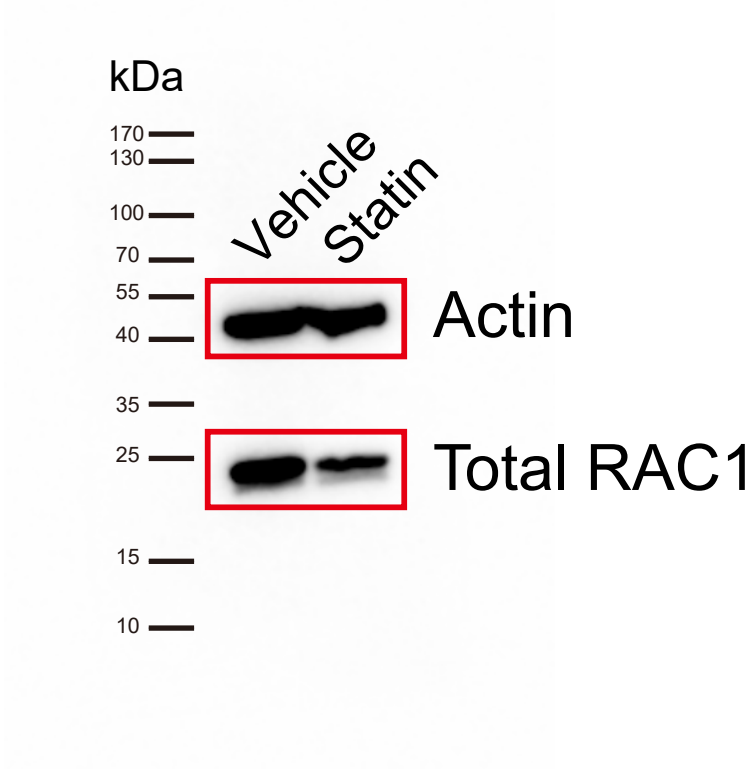


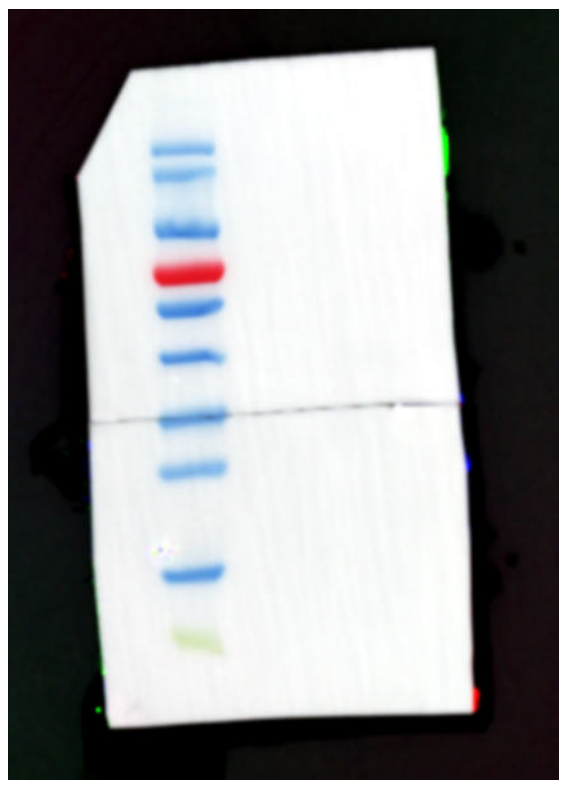

Replicate 2

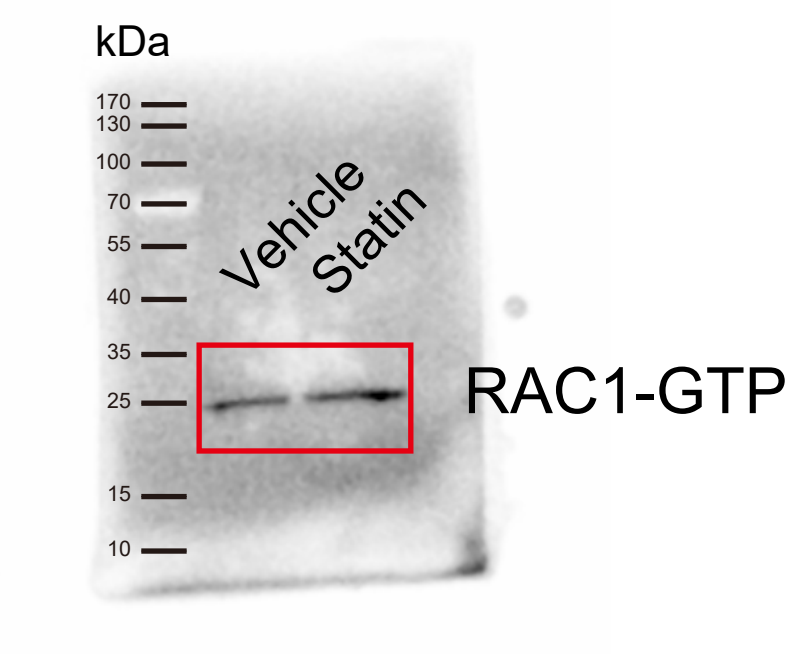


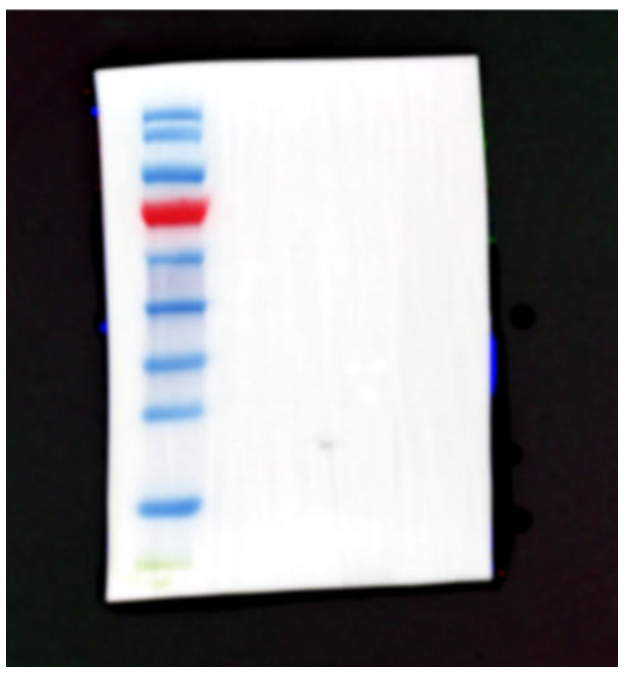

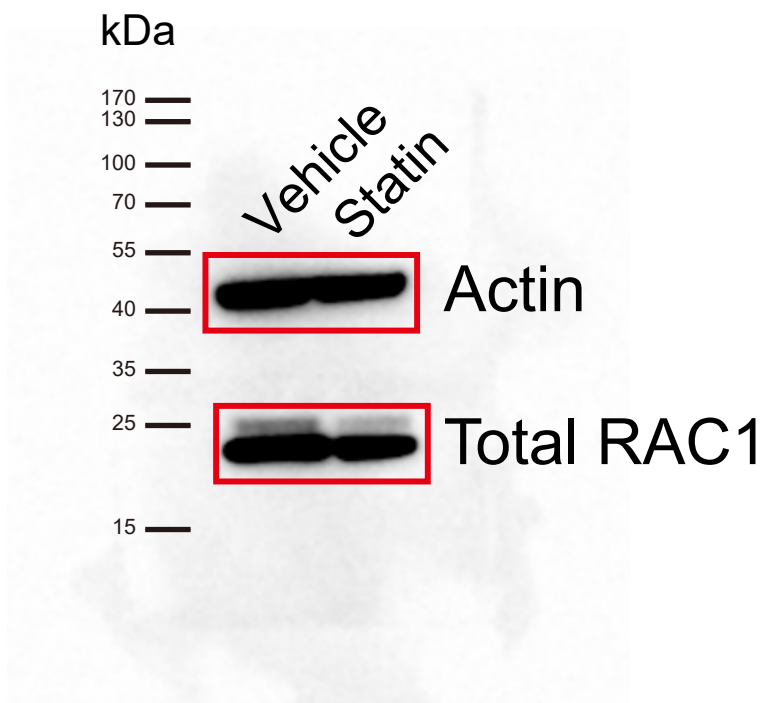


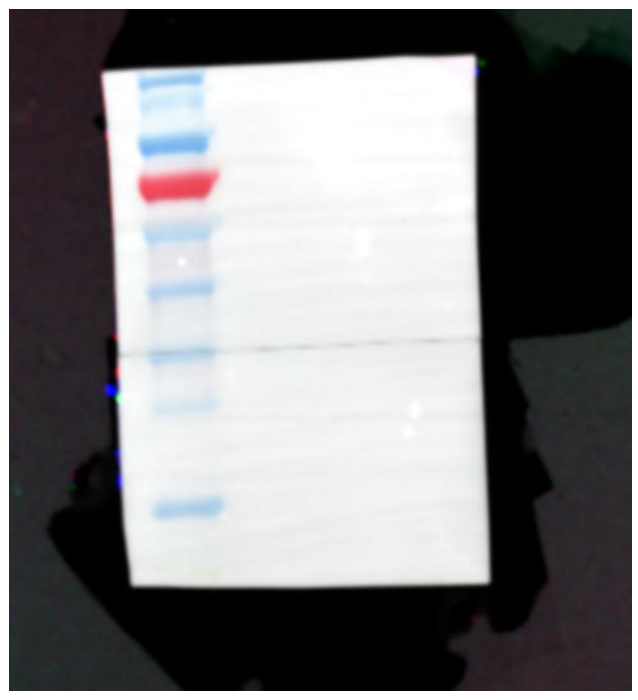

Replicate 3

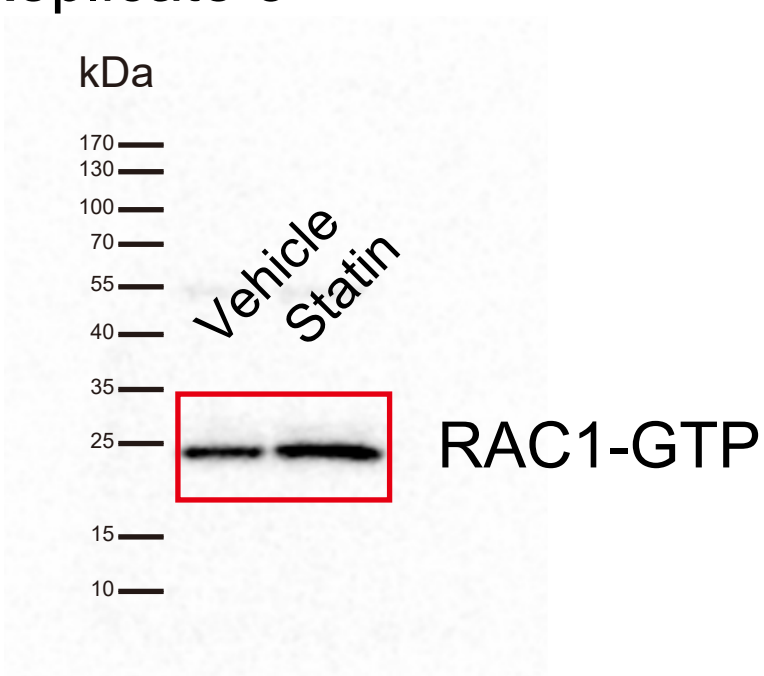


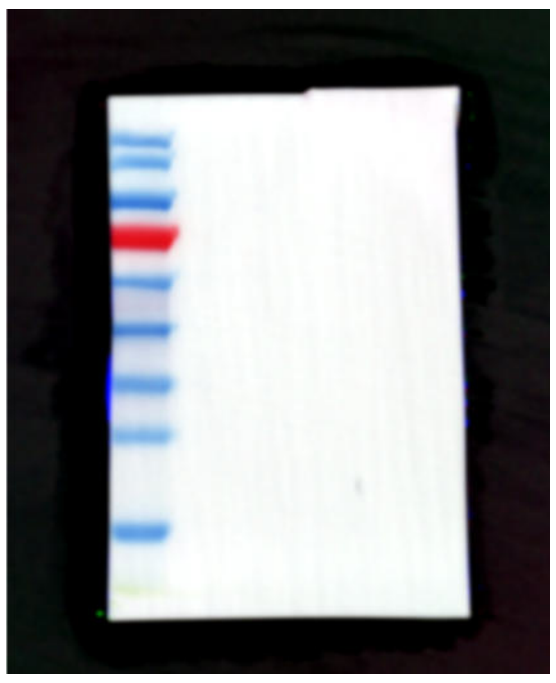

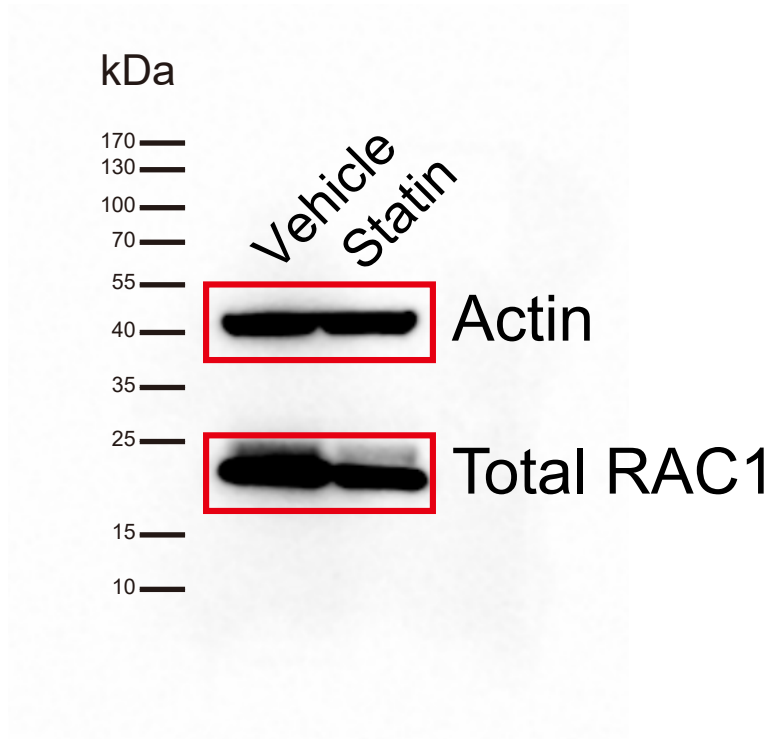


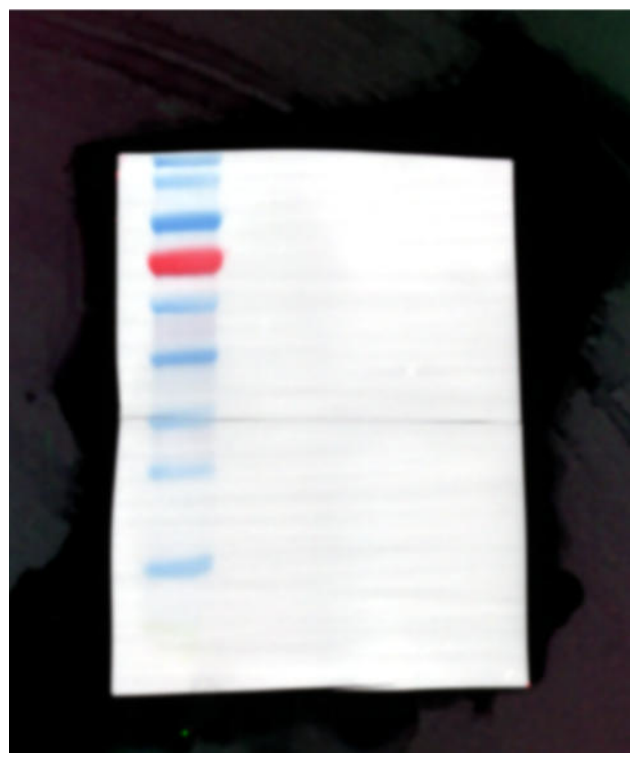

# Unprocessed images in Extended Data Figure 4

**b**

5' probe

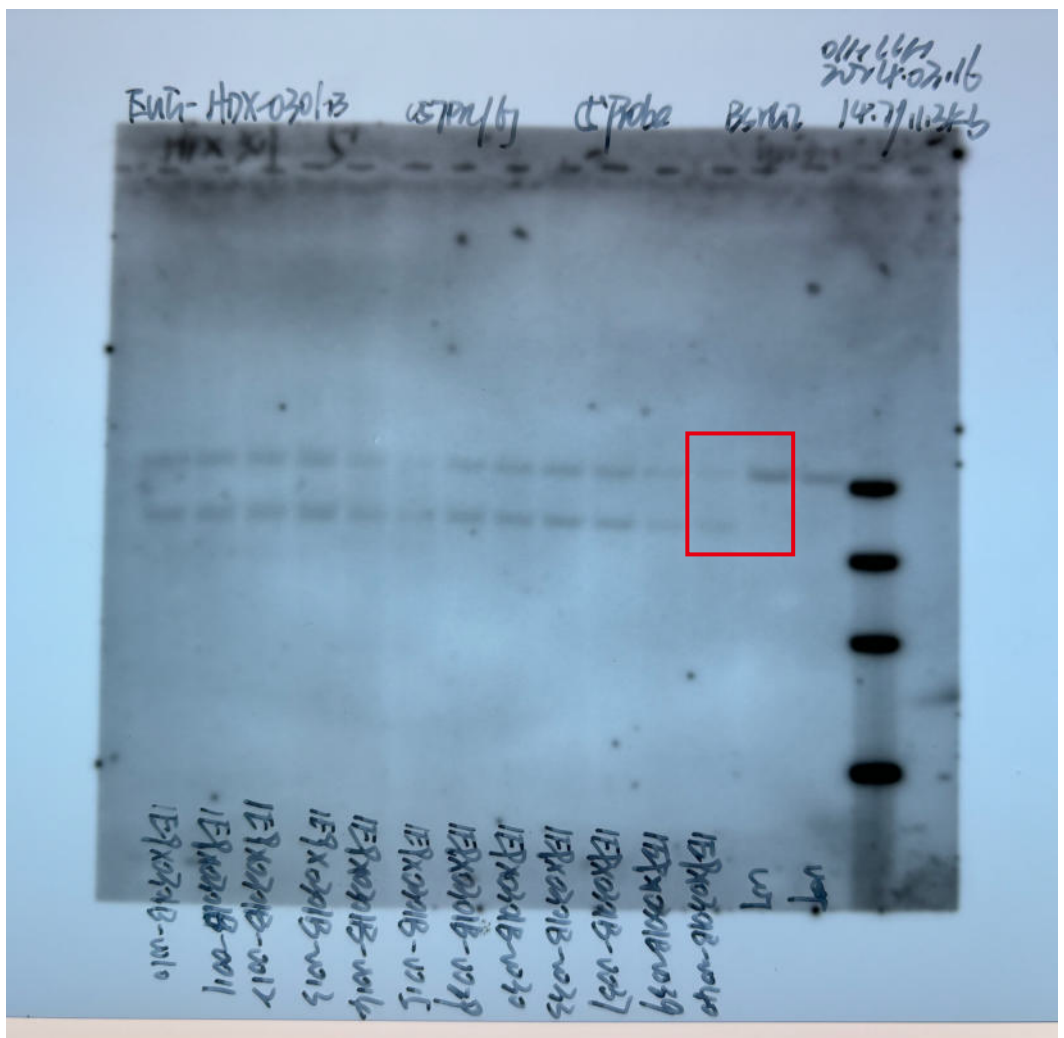

14.7 kb
11.3 kb

WPRE probe

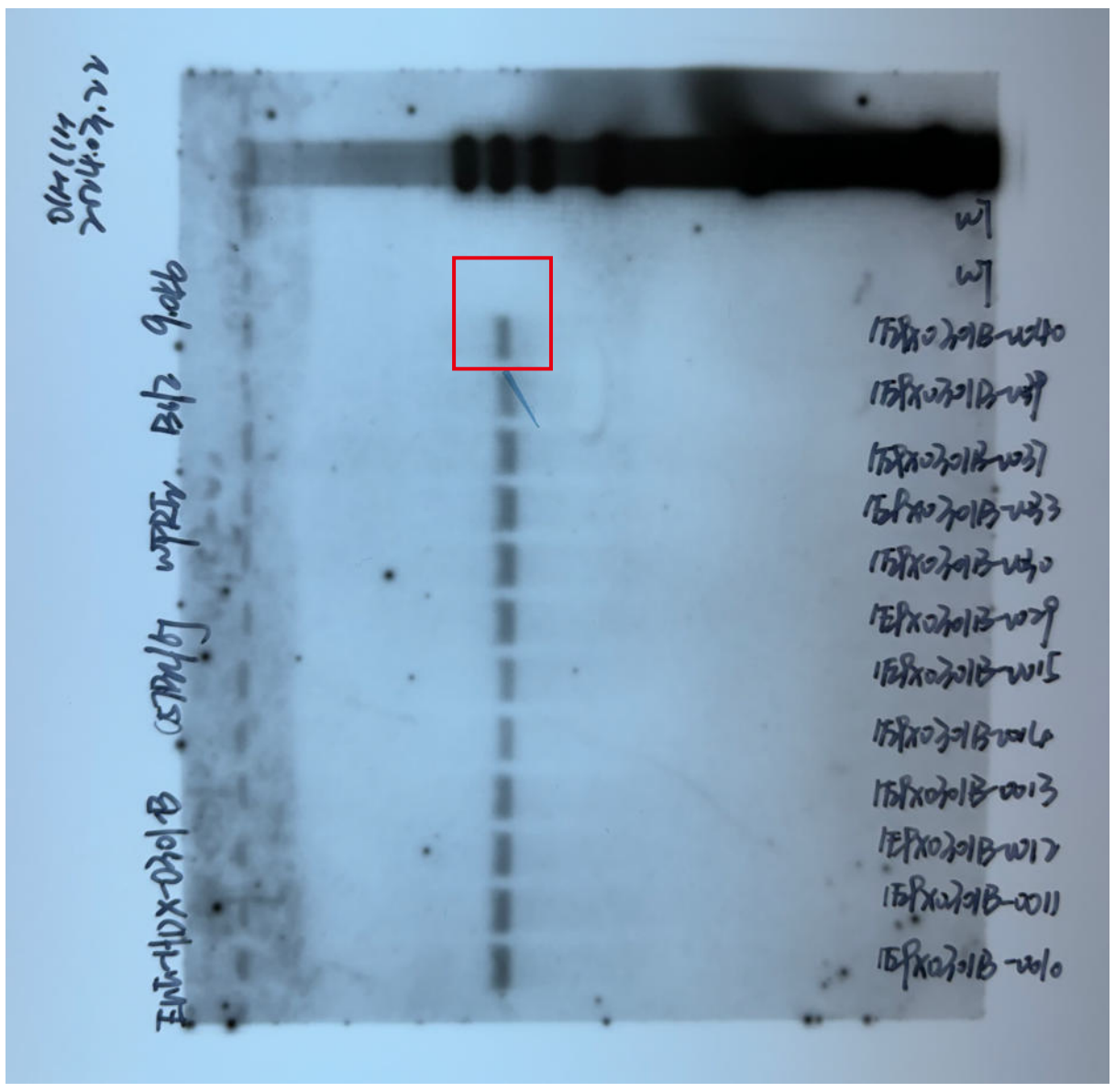

9.0 kb

**c**

*Esr1*$^{GFP/+}$ WT

381 bp
312 bp

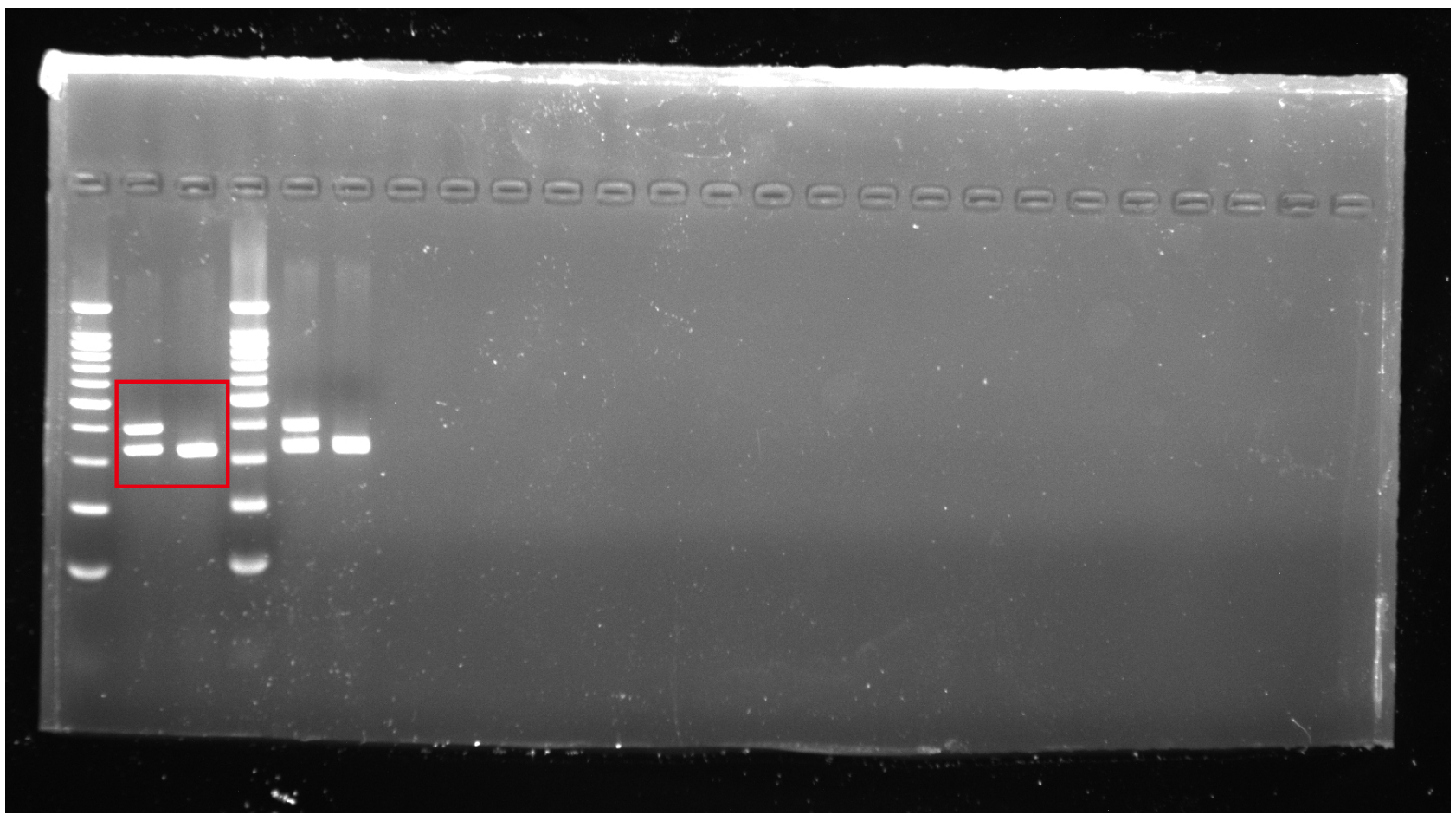

# Unprocessed images in Extended Data Figure 7

**b**

5' probe

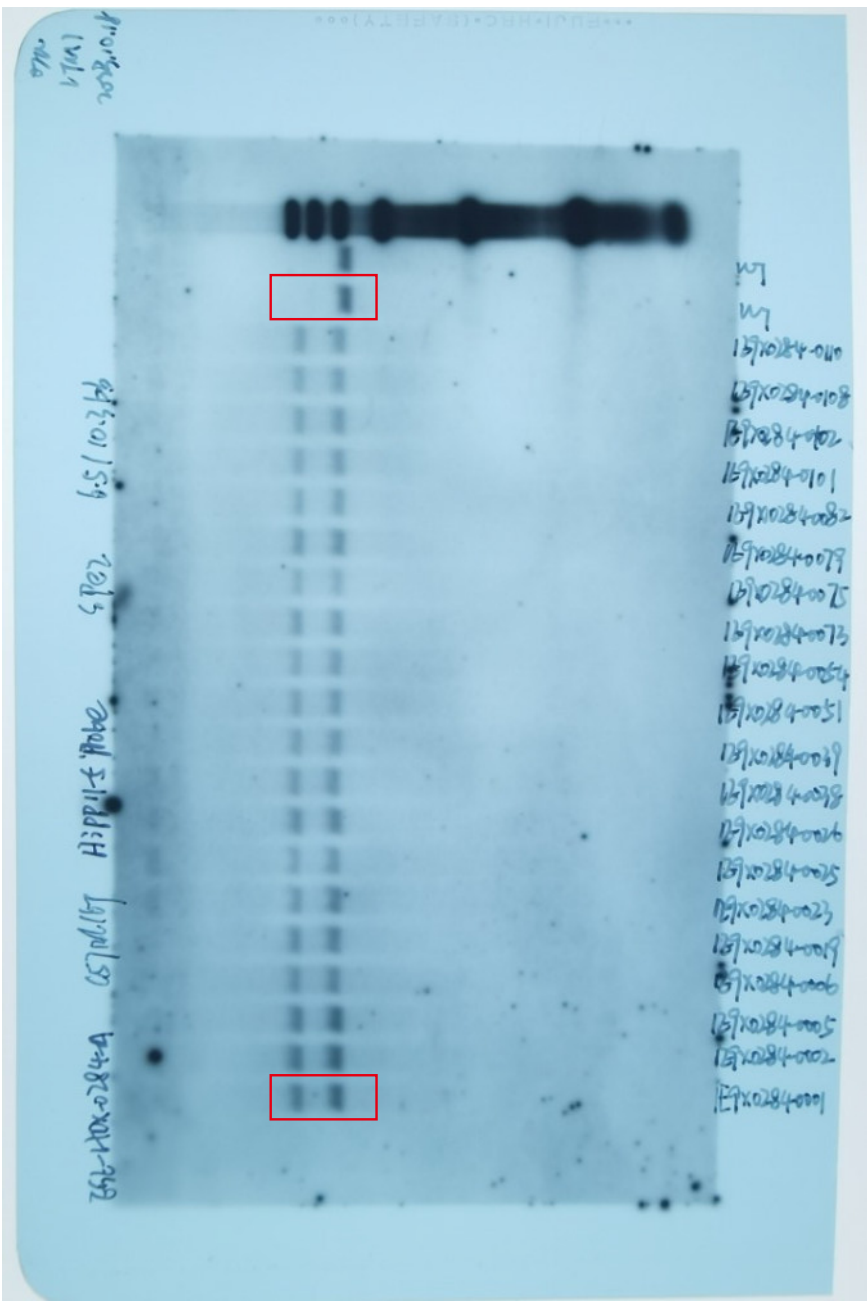

10.3 kb
6.5 kb

WPRE probe

9.3 kb

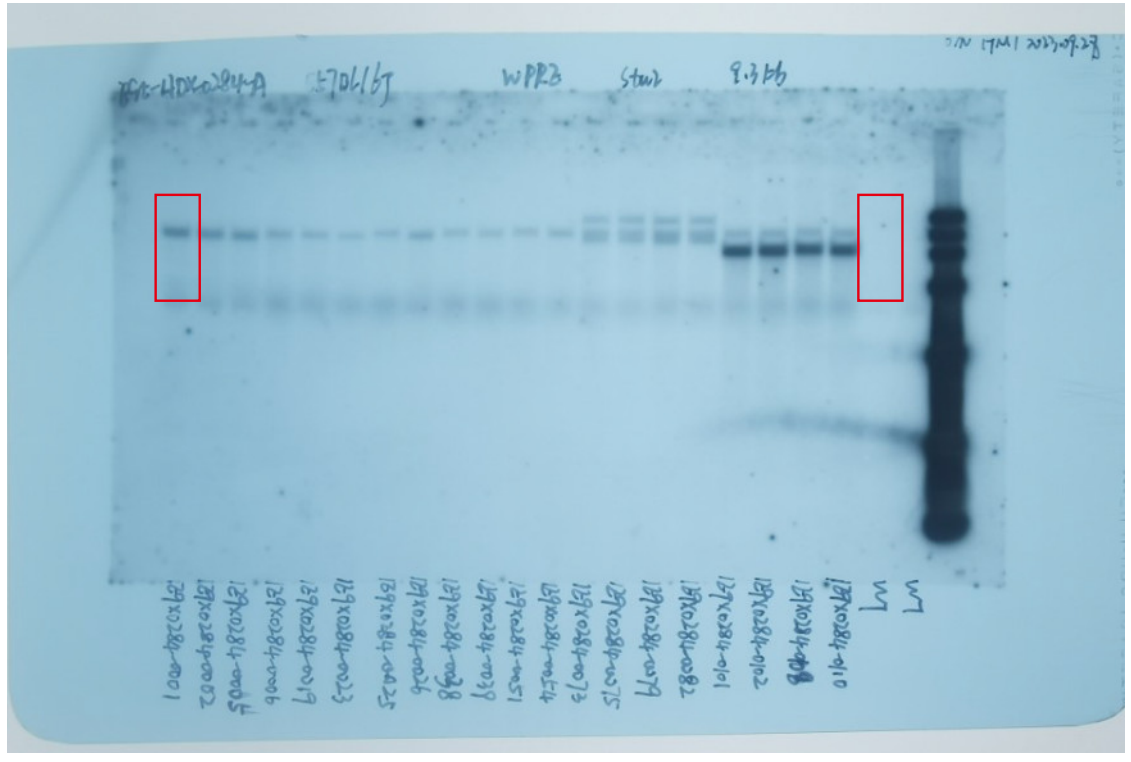

**c**

*Hipp11*$^{Oln\text{-}URE\text{-}LSL\text{-}mScarlet/+}$
WT

360 bp
209 bp

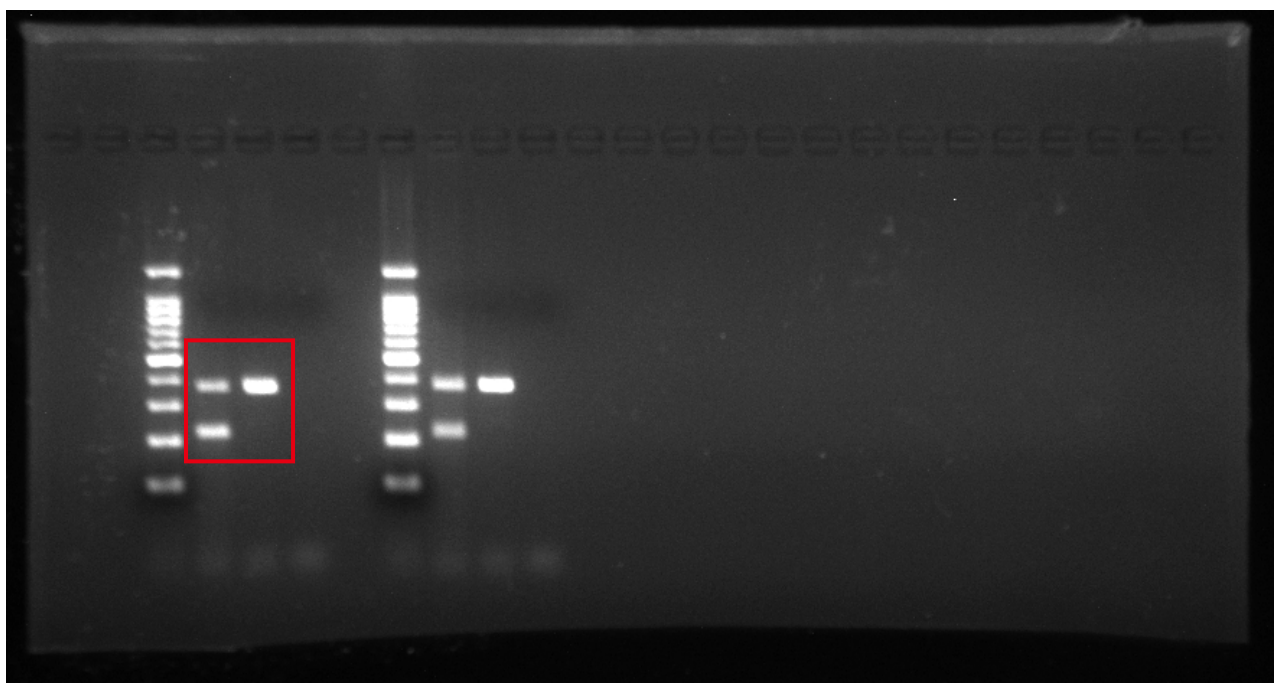

# Unprocessed images in Extended Data Figure 10

**b**

5’ probe

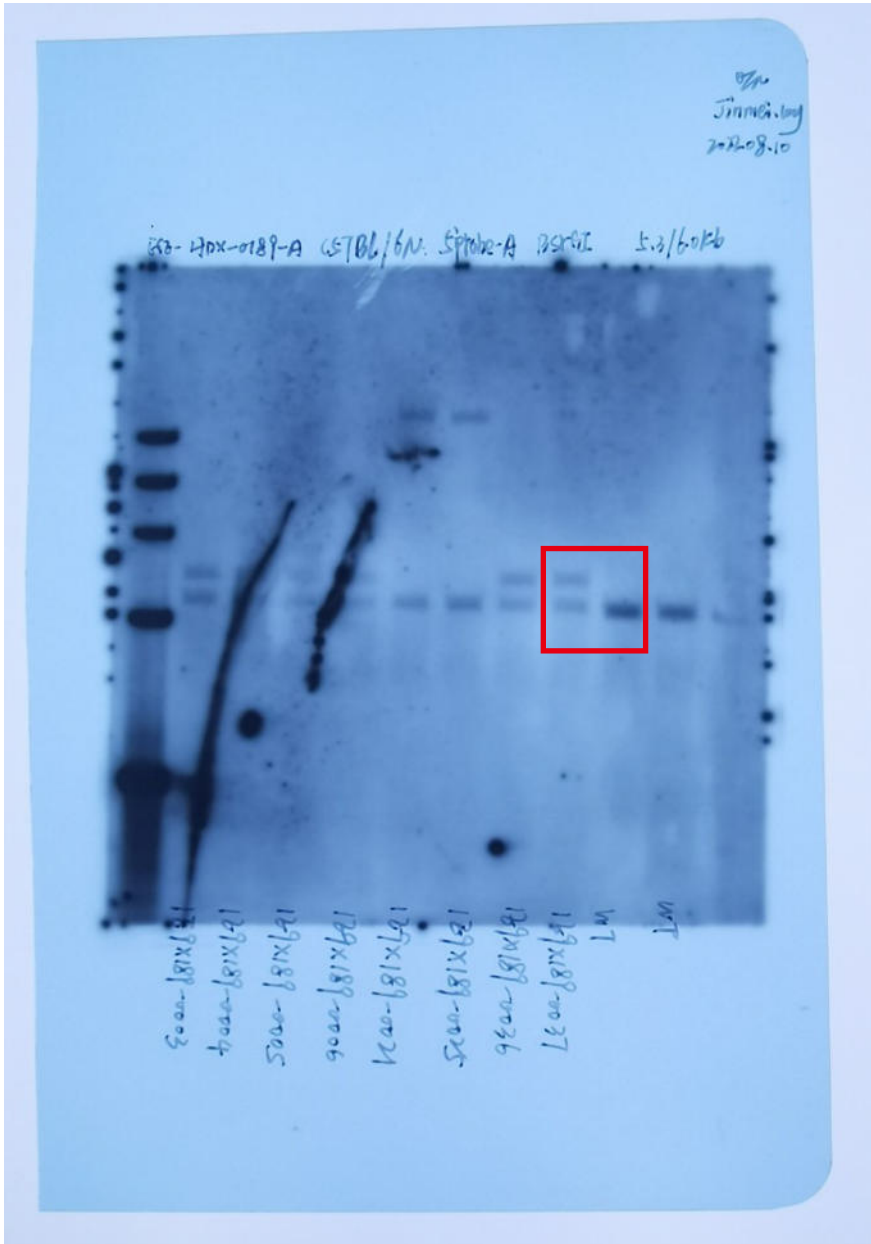

6.0 kb
5.3 kb

WPRE probe

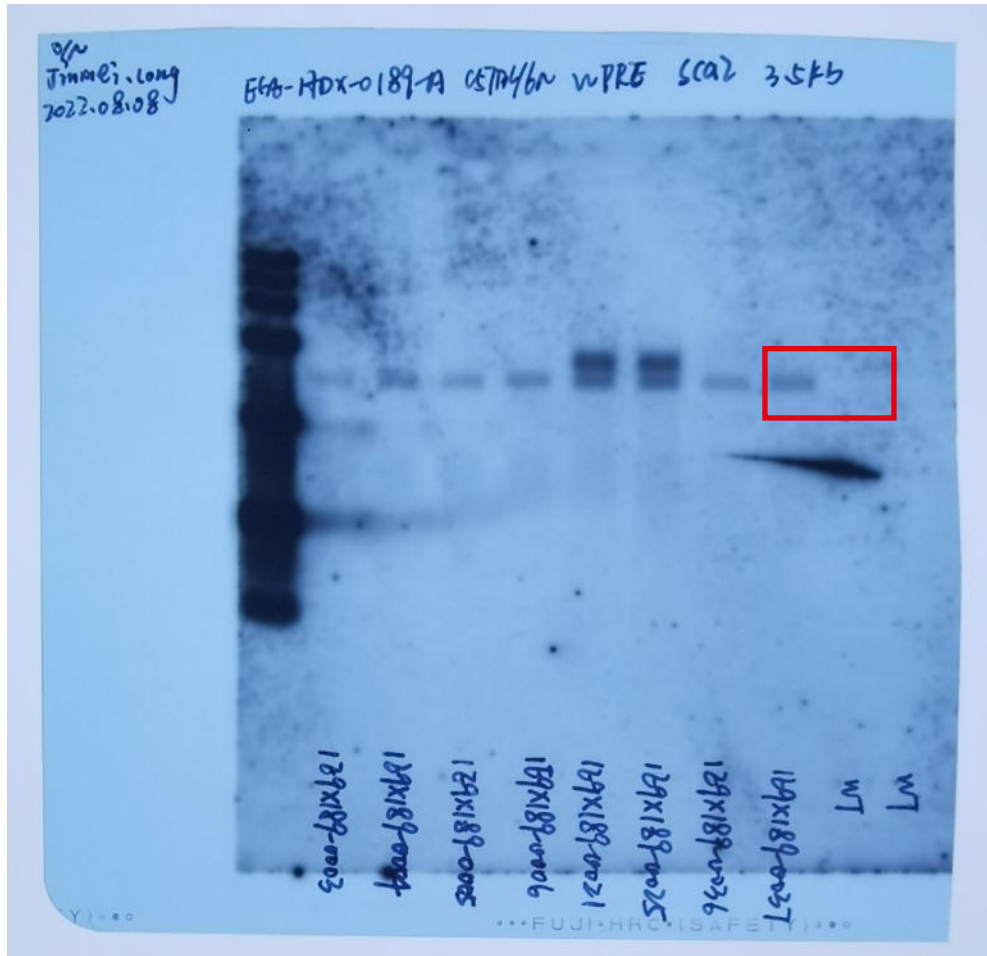

3.5 kb

**c**

*Oln*$^{NanoLuc/+}$
WT

499 bp
256 bp

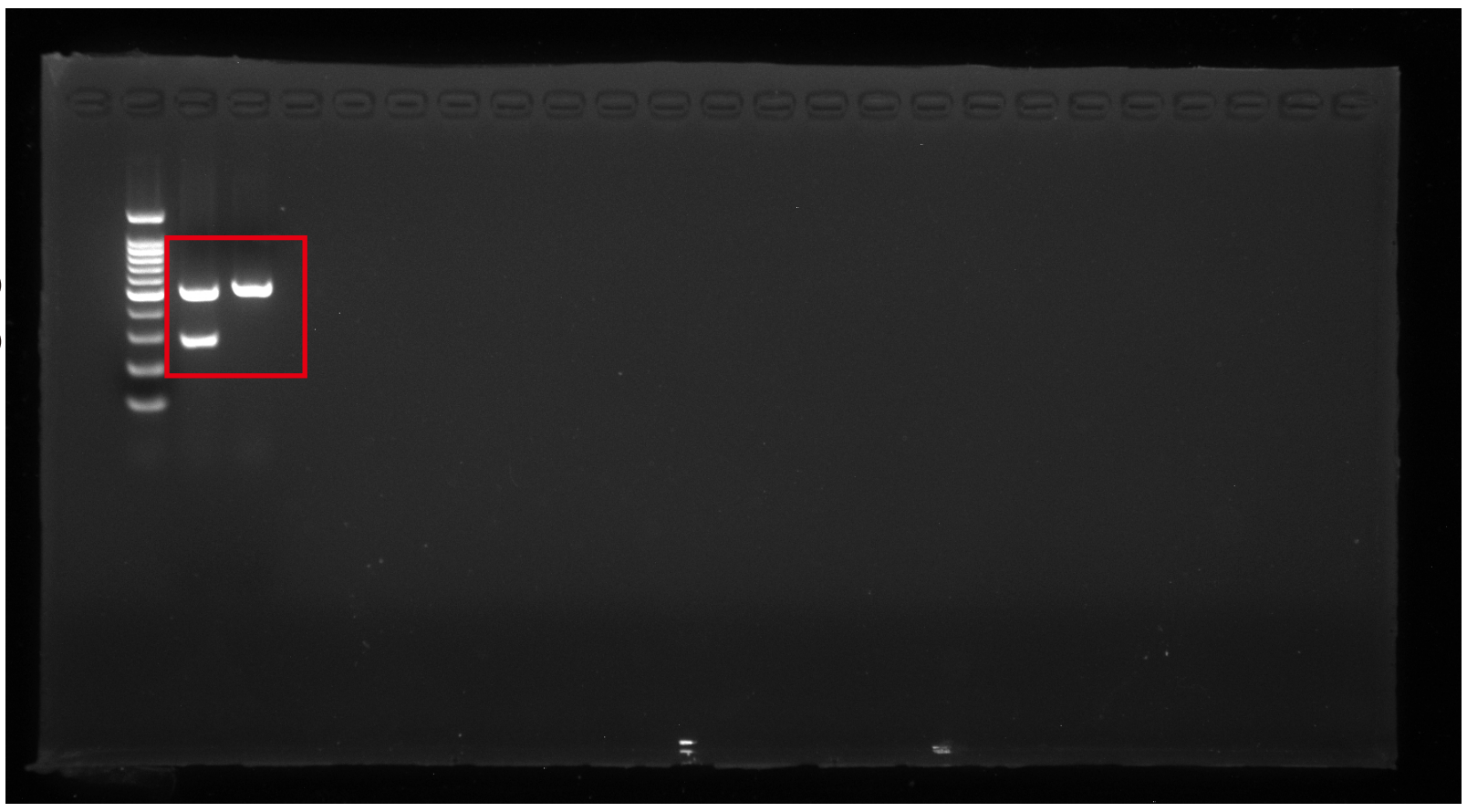

# Unprocessed images in Extended Data Figure 10

**f**

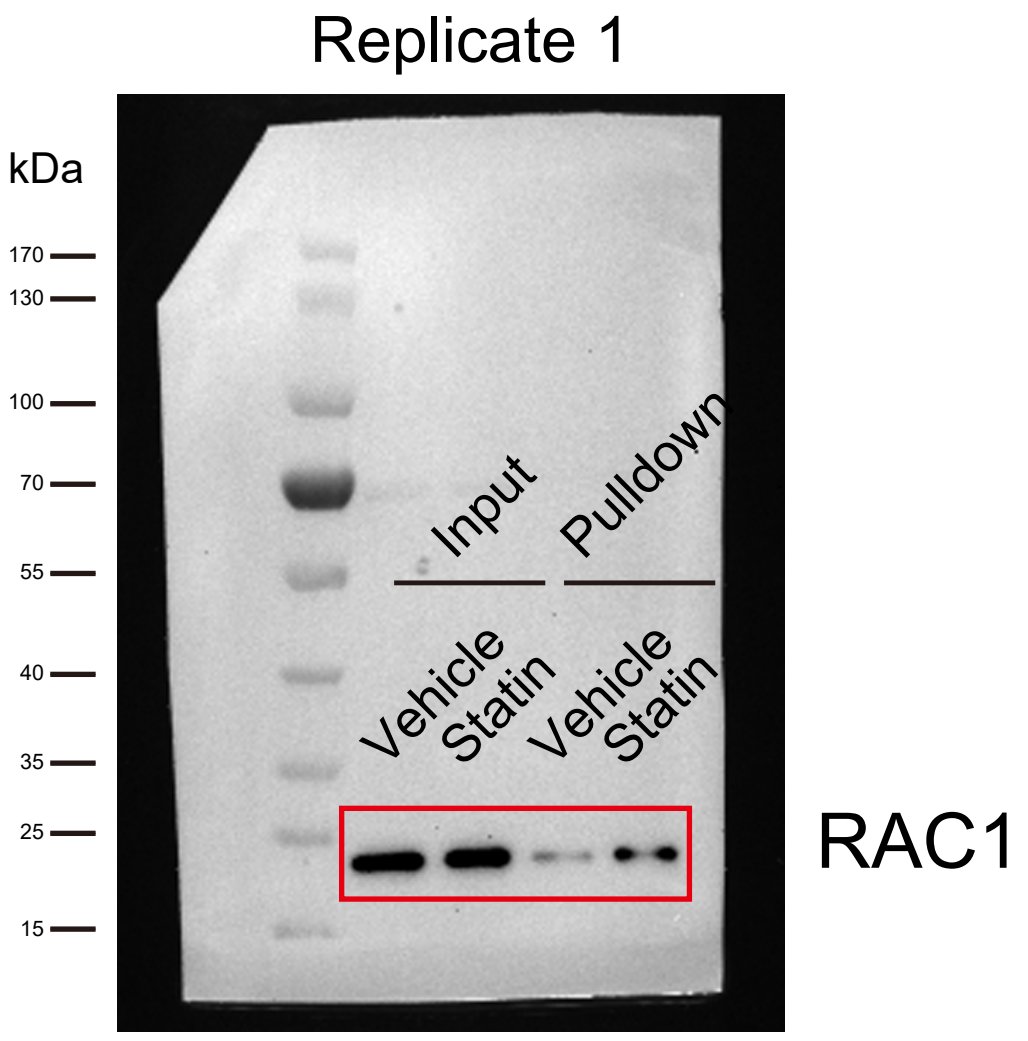


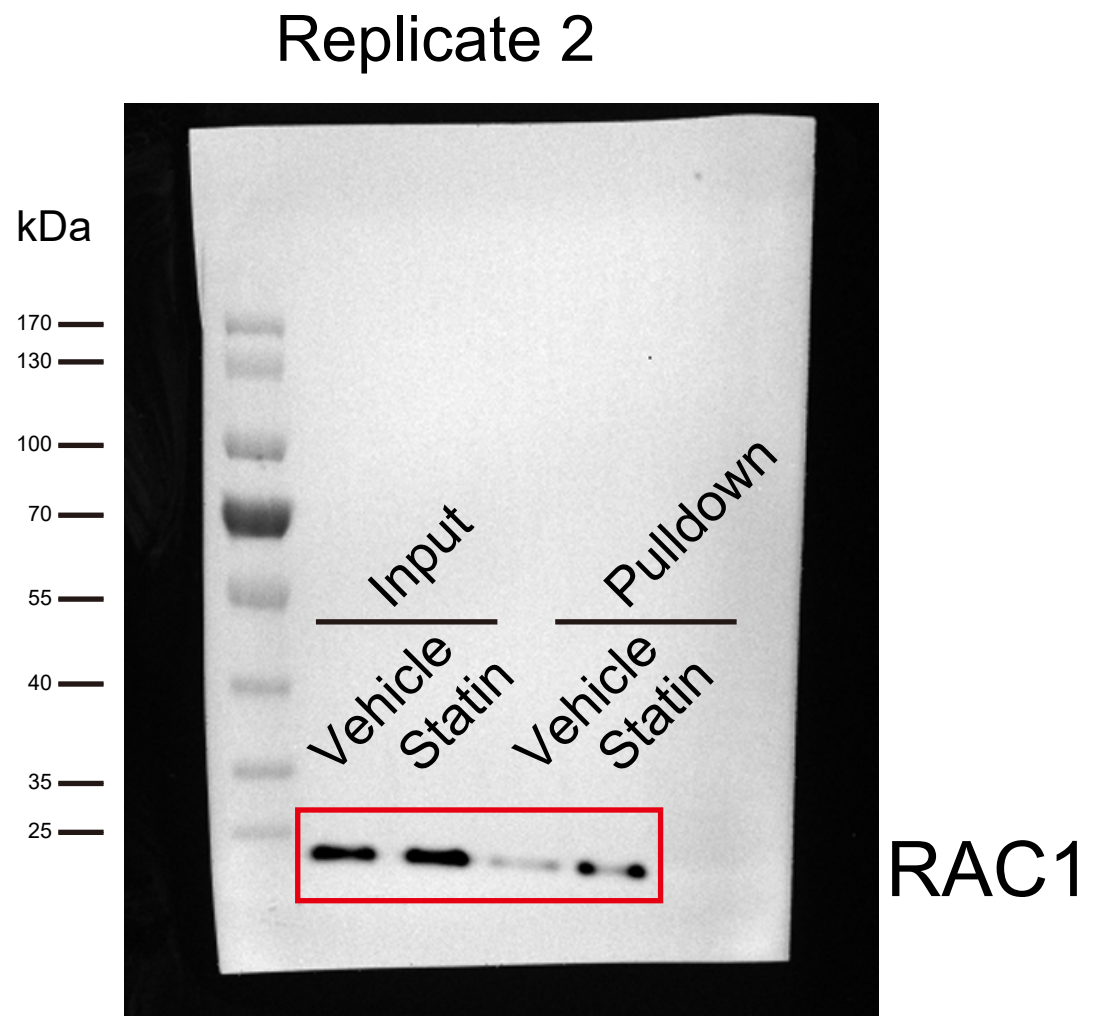


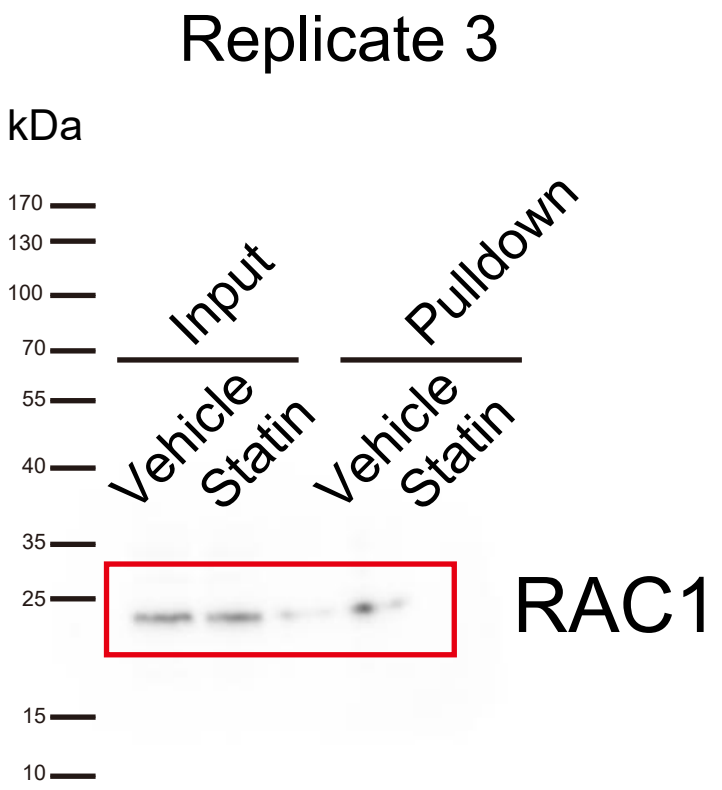


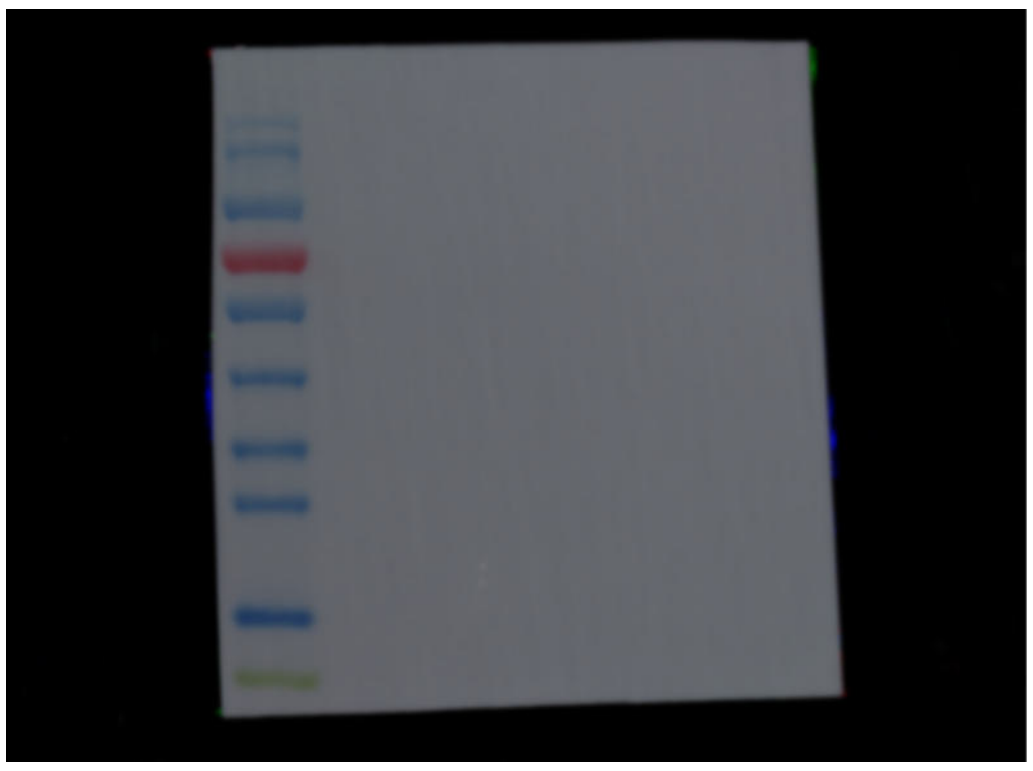

Supplement: Supplementary file 1 [file 43587_2026_1257_MOESM1_ESM.pdf]
