# Supplementary material for: Statins promote bone anabolism in estrogen deficiency by inducing osteogenic differentiation of skeletal stem/progenitor cells
Source: Nat Aging. 2026 Oct 27;6(11):2393–409. doi: 10.1038/s43587-026-01257-1 (PMC13754606; doi:10.1038/s43587-026-01257-1)
Supplement: Supplementary file 2 [file 43587_2026_1257_MOESM2_ESM.pdf]

nature portfolio

Corresponding author(s): Bo Shen

Last updated by author(s): Sep 17, 2026

Reporting Summary

Nature Portfolio wishes to improve the reproducibility of the work that we publish. This form provides structure for consistency and transparency in reporting. For further information on Nature Portfolio policies, see our Editorial Policies and the Editorial Policy Checklist.

Statistics

For all statistical analyses, confirm that the following items are present in the figure legend, table legend, main text, or Methods section.

n/a	Confirmed	
☐	☒	The exact sample size (*n*) for each experimental group/condition, given as a discrete number and unit of measurement
☐	☒	A statement on whether measurements were taken from distinct samples or whether the same sample was measured repeatedly
☐	☒	The statistical test(s) used AND whether they are one- or two-sided<br>*Only common tests should be described solely by name; describe more complex techniques in the Methods section.*
☒	☐	A description of all covariates tested
☐	☒	A description of any assumptions or corrections, such as tests of normality and adjustment for multiple comparisons
☐	☒	A full description of the statistical parameters including central tendency (e.g. means) or other basic estimates (e.g. regression coefficient) AND variation (e.g. standard deviation) or associated estimates of uncertainty (e.g. confidence intervals)
☐	☒	For null hypothesis testing, the test statistic (e.g. *F*, *t*, *r*) with confidence intervals, effect sizes, degrees of freedom and *P* value noted<br>*Give P values as exact values whenever suitable.*
☒	☐	For Bayesian analysis, information on the choice of priors and Markov chain Monte Carlo settings
☒	☐	For hierarchical and complex designs, identification of the appropriate level for tests and full reporting of outcomes
☒	☐	Estimates of effect sizes (e.g. Cohen's *d*, Pearson's *r*), indicating how they were calculated

Our web collection on statistics for biologists contains articles on many of the points above.

Software and code

Policy information about availability of computer code

Data collection	Flow cytometry data were collected using BD FACSDiva v8.0.2. Confocal images were acquired using Leica LAS X or NIS-Elements.
Data analysis	Statistical analyses were performed using GraphPad Prism v10.4.2 and R v4.2.3 with the tidyverse and broom packages. Flow cytometry data were analysed using BD FACSDiva v8.0.2 and FlowJo v10.10.0. Confocal images were processed and analysed using Leica LAS X or NIS-Elements. Three-dimensional rendering and further image analysis were performed using Bitplane Imaris v10.1.0.<br><br>The quality of RNA-seq raw reads was assessed using FastQC v0.12.1. Reads were trimmed using Cutadapt v5.0 and mapped to the mouse (GRCm39) or human (GRCh38) reference genome using STAR v2.7.11b. Gene-level counts were obtained using featureCounts v2.0.4. Differential expression analysis was performed using DESeq2 v1.38.3 in R v4.2.3. Gene set enrichment analysis (GSEA) was conducted on all genes ranked by log2 fold change using clusterProfiler v4.6.2. Functional enrichment analyses for Gene Ontology (GO) and KEGG pathways were performed on differentially expressed genes (adjusted P<0.05) using clusterProfiler v4.6.2.

For manuscripts utilizing custom algorithms or software that are central to the research but not yet described in published literature, software must be made available to editors and reviewers. We strongly encourage code deposition in a community repository (e.g. GitHub). See the Nature Portfolio guidelines for submitting code & software for further information.

Data

Policy information about availability of data

All manuscripts must include a data availability statement. This statement should provide the following information, where applicable:

- Accession codes, unique identifiers, or web links for publicly available datasets
- A description of any restrictions on data availability
- For clinical datasets or third party data, please ensure that the statement adheres to our policy

Source data files are provided with this paper. Mouse RNA sequencing and CUT&Tag raw sequencing data have been deposited in the Genome Sequence Archive (GSA) at the National Genomics Data Center (NGDC) (https://ngdc.cncb.ac.cn/gsa/) under accession number CRA036045 (PRJCA054117). Human RNA sequencing and CUT&Tag raw sequencing data have been deposited in the GSA-Human (https://ngdc.cncb.ac.cn/gsa-human/) under accession number HRA015691 (PRJCA054117), with processed data available in the OMIX database (see Data availability).

Research involving human participants, their data, or biological material

Policy information about studies with human participants or human data. See also policy information about sex, gender (identity/presentation), and sexual orientation and race, ethnicity and racism.

Reporting on sex and gender	Human samples were obtained from postmenopausal women.
Reporting on race, ethnicity, or other socially relevant groupings	Race and ethnicity were not reported in this study. Human samples were obtained from two independent hospital cohorts (Beijing Jishuitan Hospital and Suzhou Kowloon Hospital), and no race- or ethnicity-based stratification was performed, as the study focused on molecular mechanisms rather than population-level differences.
Population characteristics	Human samples included bone mineral density data from postmenopausal women aged 55–65 years and plasma samples from independent postmenopausal cohorts at Beijing Jishuitan Hospital and Suzhou Kowloon Hospital.
Recruitment	Postmenopausal women aged 55–65 years were recruited retrospectively from Beijing Jishuitan Hospital and Suzhou Kowloon Hospital based on available clinical and pharmacy records. Eligible participants had been amenorrhoeic for at least three years and had undergone at least two lumbar spine dual-energy X-ray absorptiometry (DXA) examinations separated by a minimum interval of six months. Participants were identified through hospital databases and stratified according to medication exposure records.
Ethics oversight	All human sample procurement and data analyses were conducted in accordance with the Declaration of Helsinki and were approved by the Ethics Committees of Beijing Jishuitan Hospital and Suzhou Kowloon Hospital. For retrospective, observational analyses of dual-energy X-ray absorptiometry (DXA) data and pharmacy dispensing and administration records, the requirement for informed consent was waived by the respective Ethics Committees. Written informed consent was obtained from all participants who provided plasma or bone marrow samples.

Note that full information on the approval of the study protocol must also be provided in the manuscript.

Field-specific reporting

Please select the one below that is the best fit for your research. If you are not sure, read the appropriate sections before making your selection.

☒ Life sciences ☐ Behavioural & social sciences ☐ Ecological, evolutionary & environmental sciences

For a reference copy of the document with all sections, see nature.com/documents/nr-reporting-summary-flat.pdf

Life sciences study design

All studies must disclose on these points even when the disclosure is negative.

Sample size	Samples sizes were not pre-determined based on statistical power calculations but were based on our experience with these assays. For assays in which variability is commonly high, we typically used n>5. For assays in which variability is commonly low, we typically used n<5.
Data exclusions	No data were excluded.
Replication	The experimental findings were reproduced in multiple independent experiments. The number of independent experiments and biological replicates for each data panel is indicated in the figure panel itself and in the source data files. Data in the figures represent the aggregate of all independent experiments in most cases. Data in a minority of panels are from a representative experiment (e.g. for Immunofluorescence imaging) and in those cases the number of independent experiments that reproduced the finding is also indicated in the figure legends.
Randomization	No formal randomization techniques were used; however, samples were allocated randomly to experiments and processed in an arbitrary order.
Blinding	No formal blinding was applied when performing the experiments or analyzing the data. Blinding was not performed during data collection or analysis.

Reporting for specific materials, systems and methods

We require information from authors about some types of materials, experimental systems and methods used in many studies. Here, indicate whether each material, system or method listed is relevant to your study. If you are not sure if a list item applies to your research, read the appropriate section before selecting a response.

Materials & experimental systems

n/a	Involved in the study
☐	☒ Antibodies
☐	☒ Eukaryotic cell lines
☒	☐ Palaeontology and archaeology
☐	☒ Animals and other organisms
☒	☐ Clinical data
☒	☐ Dual use research of concern
☒	☐ Plants

Methods

n/a	Involved in the study
☐	☒ ChIP-seq
☐	☒ Flow cytometry
☒	☐ MRI-based neuroimaging

Antibodies

Antibodies used

The following antibodies were used in this study.

Flow cytometry:
Anti-mouse CD31 (clone 390, FITC-conjugated, Invitrogen, Cat. #11-0311-85; 1:200); Anti-mouse CD45 (clone 30-F11, FITC-conjugated, Invitrogen, Cat. #11-0451-85; 1:200); Anti-mouse TER119 (clone TER-119, FITC-conjugated, BioLegend, Cat. #116206; 1:200); Anti-mouse CD31 (clone 390, BV421-conjugated, BioLegend, Cat. #102424; 1:200); Anti-mouse CD45 (clone 30-F11, BV421-conjugated, BioLegend, Cat. #103134; 1:200); Anti-mouse TER119 (clone TER-119, BV421-conjugated, BioLegend, Cat. #116234; 1:200); Anti-VE-cadherin (clone BV13, eFluor™ 660-conjugated, Invitrogen, Cat. #50-1441-82; 10 ug/mouse); Anti-mouse/human leptin receptor (clone EPR28293-79, Alexa Fluor® 647-conjugated; Abcam, Cat. #ab323265; 1:200).

Anti-human CD31 (clone WM59, FITC-conjugated, BioLegend, Cat. #303104; 1:200); Anti-human CD45 (clone 2D1, FITC-conjugated, BioLegend, Cat. #368508; 1:200); Anti-human CD71 (clone CY1G4, FITC-conjugated, BioLegend, Cat. #334104; 1:200).

Immunofluorescence:
Anti-Discosoma tdTomato (polyclonal, unconjugated; LSBio, Cat. #LS-C340696; 1:250); Anti-green fluorescent protein (GFP) (polyclonal, unconjugated; Aves Labs, Cat. #GFP-1020; 1:250); Anti-endomucin (polyclonal, unconjugated; R&D Systems, Cat. #AF4666; 1:250); Anti-perilipin-1 (clone D1D8, unconjugated; Cell Signaling Technology, Cat. #9349S; 1:1,000); Anti-tRFP/BFP (polyclonal, unconjugated; Evrogen, Cat. #AB233; 1:200).

Alexa Fluor 555-conjugated donkey anti-goat IgG (Invitrogen, Cat. #A-21432; 1:500); Alexa Fluor 647-conjugated AffiniPure F(ab')$_2$ donkey anti-rabbit IgG (Jackson ImmunoResearch, Cat. #711-606-152; 1:250); Alexa Fluor 488-conjugated AffiniPure F(ab')$_2$ donkey anti-chicken IgG (Jackson ImmunoResearch, Cat. #703-546-155; 1:250).

ChIP-seq:
Anti-ERα (clone F-10, unconjugated; Santa Cruz, Cat. #sc-8002; 1:50); Anti-ERα (polyclonal, unconjugated; EpiCypher, Cat. #13-2011; 1:100); Anti-NF-κB p65 (clone D14E12, unconjugated; Cell Signaling Technology, Cat. #8242; 1:50); Anti-NF-κB p65 (clone EPR21781, unconjugated; Abcam, Cat. #ab218533; 1:50).

Western blotting:
Anti-ERα (clone F-10, unconjugated; Santa Cruz, Cat. #sc-8002; 1:50); Anti-ERα (clone 33, unconjugated; Invitrogen, Cat. #MA1-310; 1:200); Anti-RAC1 (clone 4A4B11, unconjugated; Proteintech, Cat. #66122-1-PBS; 1:1000); Anti-GAPDH (polyclonal, unconjugated; Proteintech, Cat. #10494-1-AP; 1:1000); Anti-Actin (polyclonal, unconjugated; Proteintech, Cat. #20536-1-AP; 1:4000).

Alexa Fluor Plus 647-conjugated donkey anti-mouse IgG (Invitrogen, Cat. #A32787; 1:1000); Alexa Fluor 488-conjugated AffiniPure® F(ab')$_2$ donkey anti-rabbit IgG (Jackson ImmunoResearch, Cat. #711-546-152; 1:1000); HRP-conjugated goat anti-mouse IgG (Invitrogen, Cat. #31430; 1:5000); HRP-conjugated goat anti-rabbit IgG (Invitrogen, Cat. #31460; 1:10000).

Validation

All antibodies used in this study are commercially available and have been validated by the manufacturers and in previously published studies (for example, Nature 495:231). Antibodies central to the conclusions of this study were further validated in-house. In particular, the anti-leptin receptor antibody was validated using mouse bone marrow cells deficient in Lepr, as previously described (Bone Res 13:6).

Eukaryotic cell lines

Policy information about cell lines and Sex and Gender in Research

Cell line source(s)

HEK293T cells were obtained from China Center for Type Culture Collection (Cat. #GDC0187)

Authentication	Cell lines were authenticated by the suppliers. HEK293T cells were routinely monitored for morphology and growth characteristics.
Mycoplasma contamination	All cell lines used in this study tested negative for mycoplasma contamination.
Commonly misidentified lines (See ICLAC register)	HEK293T cells used in this study are not listed in the ICLAC database of commonly misidentified cell lines.

Animals and other research organisms

Policy information about studies involving animals; ARRIVE guidelines recommended for reporting animal research, and Sex and Gender in Research

Laboratory animals	Esr1 floxed (JAX: 032173), Rac1 floxed (GemPharmatech: T009642), Rosa26-CAG-loxP-stop-loxP-tdTomato (Ai14, JAX: 007914), Lepr-TriBFP (JAX: 040198), Rosa26-Cas9 (JAX: 026179), Col1a1*2.3-eGFP (JAX: 013134), and Col1a1-CreER (JAX: 016241) mouse lines were used in this study.<br><br>Tet-On Lepr-Cre, Esr1-TriGFP, Oln-NanoLuc and Hipp11-Oln-URE-LSL-mScarlet mouse lines were generated in this study.<br><br>All mice were maintained on a C57BL/6J genetic background and were aged 2–18 months at the time of experiments.
Wild animals	No wild animals were used.
Reporting on sex	Both male and female mice were used.
Field-collected samples	No field-collected samples were used.
Ethics oversight	All mouse experiments were conducted in compliance with institutional and national ethical guidelines and were approved by the Institutional Animal Care and Use Committee of the National Institute of Biological Sciences, Beijing (approval number NIBS2026M0042). Mice were maintained under pathogen-free conditions of a 12h light/dark cycle at controlled temperature (20-25°C) and humidity (50-70%), and were provided with food and water ad libitum.

Note that full information on the approval of the study protocol must also be provided in the manuscript.

Plants

Seed stocks	Not used
Novel plant genotypes	Not used
Authentication	Not used

ChIP-seq

Data deposition

☒ Confirm that both raw and final processed data have been deposited in a public database such as GEO.

☒ Confirm that you have deposited or provided access to graph files (e.g. BED files) for the called peaks.

Data access links<br>*May remain private before publication.*	Mouse CUT&Tag raw sequencing data have been deposited in the Genome Sequence Archive (GSA) at the National Genomics Data Center (NGDC) (https://ngdc.cncb.ac.cn/gsa/) under accession number CRA036045 (PRJCA054117). Human CUT&Tag raw sequencing data have been deposited in the GSA-Human (https://ngdc.cncb.ac.cn/gsa-human/) under accession number HRA015691 (PRJCA054117).<br><br>processed data:<br>OMIX013951<br>OMIX013945<br><br>raw data:<br>HRA015691<br>CRA036045
Files in database submission	M_Esr1_Vehicle_1.R1.fastq.gz<br>M_Esr1_Vehicle_1.R2.fastq.gz

M_Esr1_Vehicle_1_FE.bw
M_Esr1_Vehicle_2.R1.fastq.gz
M_Esr1_Vehicle_2.R2.fastq.gz
M_Esr1_Vehicle_2_FE.bw
M_Esr1_E2_1.R1.fastq.gz
M_Esr1_E2_1.R2.fastq.gz
M_Esr1_E2_1_FE.bw
M_Esr1_E2_2.R1.fastq.gz
M_Esr1_E2_2.R2.fastq.gz
M_Esr1_E2_2_FE.bw
M_Nfkb_Ctrl_1.R1.fastq.gz
M_Nfkb_Ctrl_1.R2.fastq.gz
M_Nfkb_Ctrl_1_FE.bw
M_Nfkb_Ctrl_2.R1.fastq.gz
M_Nfkb_Ctrl_2.R2.fastq.gz
M_Nfkb_Ctrl_2_FE.bw
M_Nfkb_Statin_1.R1.fastq.gz
M_Nfkb_Statin_1.R2.fastq.gz
M_Nfkb_Statin_1_FE.bw
M_Nfkb_Statin_2.R1.fastq.gz
M_Nfkb_Statin_2.R2.fastq.gz
M_Nfkb_Statin_2_FE.bw
H_ESR1_Vehicle_1.R1.fastq.gz
H_ESR1_Vehicle_1.R2.fastq.gz
H_ESR1_Vehicle_1_FE.bw
H_ESR1_Vehicle_2.R1.fastq.gz
H_ESR1_Vehicle_2.R2.fastq.gz
H_ESR1_Vehicle_2_FE.bw
H_ESR1_E2_1.R1.fastq.gz
H_ESR1_E2_1.R2.fastq.gz
H_ESR1_E2_1_FE.bw
H_ESR1_E2_2.R1.fastq.gz
H_ESR1_E2_2.R2.fastq.gz
H_ESR1_E2_2_FE.bw
H_NFKB_Ctrl1.R1.fastq.gz
H_NFKB_Ctrl1.R2.fastq.gz
H_NFKB_Ctrl_1_FE.bw
H_NFKB_Ctrl2.R1.fastq.gz
H_NFKB_Ctrl2.R2.fastq.gz
H_NFKB_Ctrl_2_FE.bw
H_NFKB_Statin_1.R1.fastq.gz
H_NFKB_Statin_1.R2.fastq.gz
H_NFKB_Statin_1_FE.bw
H_NFKB_Statin2.R1.fastq.gz
H_NFKB_Statin2.R2.fastq.gz
H_NFKB_Statin_2_FE.bw

Genome browser session
(e.g. UCSC)

no longer applicable

Methodology

Replicates

2 Replicates for each group

Sequencing depth

20 million reads, 150 bp length of reads, paired-end reads

Antibodies

Anti-ERα (clone F-10, unconjugated; Santa Cruz, Cat. #sc-8002; 1:50);
Anti-ERα (polyclonal, unconjugated; EpiCypher, Cat. #13-2011; 1:100);
Anti-NF-κB p65 (clone D14E12, unconjugated; Cell Signaling Technology, Cat. #8242; 1:50);
Anti-NF-κB p65 (clone EPR21781, unconjugated; Abcam, Cat. #ab218533; 1:50).

Peak calling parameters

Peak calling was performed using MACS2 (parameter- q limit: 0.05). Baseline noise was estimated using MACS2's internal background modeling function. The conversion from bedGraph (bdg) to bigWig (bw) format is typically performed using bedGraphToBigWig, a utility from the UCSC Genome Browser tools suite.

Data quality

Adapter sequences and low-quality bases were removed using Trim Galore (parameter- quality limit: 30), and peak calling was performed using MACS2 (parameter- q limit: 0.05).

Software

TrimGalore v0.6.6, Bowtie2 v2.3.5.1, SAMtools v1.7, MACS2 v2.2.7.1, IGV v2.19.4

Flow Cytometry

Plots

Confirm that:

☒ The axis labels state the marker and fluorochrome used (e.g. CD4-FITC).

☒ The axis scales are clearly visible. Include numbers along axes only for bottom left plot of group (a 'group' is an analysis of identical markers).

☒ All plots are contour plots with outliers or pseudocolor plots.

☒ A numerical value for number of cells or percentage (with statistics) is provided.

Methodology

Sample preparation	For flow cytometric analysis of stromal cells, bone marrow was flushed using HBSS-free with 2% bovine serum. Then whole bone marrow was digested with type I collagenase (3 mg/ml), dispase (4 mg/ml) and DNase I (1 U/ml) at 37°C for 30 min. Samples were then stained with antibodies and analyzed by flow cytometry.
Instrument	BD FACS Aria Fusion or III (for cell sorting or analysis)
Software	BD FACSDiva v8.0, FlowJo v10
Cell population abundance	The abundance of the relevant cell populations within post-sort fractions was 90-100% in experiments
Gating strategy	Mouse bone marrow stromal cells were typically isolated as cells that were positive for Leptin Receptor and negative for mouse endothelial and hematopoietic markers (CD31, CD45, TER119) as shown in Extended Data Fig. 4f. Osteoblasts were isolated as cells that were positive for Col1a1*2.3-eGFP and negative for hematopoietic markers (CD31, CD45, TER119) as shown in Extended Data Fig. 4g. Endothelial cells were isolated as cells that were positive for CDH5 and negative for hematopoietic markers (CD45, TER119) as shown in Extended Data Fig. 4h. Mouse whole bone marrow cells were isolated as negative for TER119 as shown in Extended Data Fig. 4i.<br><br>Human bone marrow stromal cells were typically isolated as cells that were positive for Leptin Receptor and negative for human endothelial and hematopoietic markers (CD31, CD45, CD71) as shown in Extended Data Fig. 4j. Human whole bone marrow cells were isolated as negative for CD71 as shown in Extended Data Fig. 4k.<br><br>To eliminate dead cells from sorts and analyses, cells were stained with 4',6-diamidino-2-phenylindole (DAPI) or propidum iodide (PI).

☒ Tick this box to confirm that a figure exemplifying the gating strategy is provided in the Supplementary Information.
