# Supplementary material for: Statins promote bone anabolism in estrogen deficiency by inducing osteogenic differentiation of skeletal stem/progenitor cells
Source: Nat Aging. 2026 Oct 27;6(11):2393–409. doi: 10.1038/s43587-026-01257-1 (PMC13754606; doi:10.1038/s43587-026-01257-1)
Supplement: Supplementary file 3 [file 43587_2026_1257_MOESM3_ESM.pdf]

nature portfolio

Peer Review File

Statins promote bone anabolism in estrogen deficiency by inducing osteogenic differentiation of skeletal stem/progenitor cells

Corresponding Author: Dr Bo Shen

Version 0:

Reviewer comments:

Reviewer #1

(Remarks to the Author)
This is a well-conducted and timely study addressing an important clinical and biological question, namely the link between statin use and bone mineral density in estrogen-deficient conditions. The integration of human cohort data with mechanistic investigations in mouse models and cellular systems is a clear strength and provides a compelling framework for the proposed mechanism involving osteolectin. I also appreciate the relatively concise discussion, which is appropriate given that the results are clear and directly support the main conclusions. Overall, the manuscript is of broad interest and provides valuable insight.

I suggest the following comments to further strengthen clarity and interpretation:

- The manuscript states that three or four independent experiments were performed for most animal studies. It would be important to clarify how these experiments were conducted, how variability between them was addressed, and on what basis one dataset was chosen as representative.

- Figures 2f and 2k: Please ensure that the representative images display trabecular bone from the same anatomical location within the metaphyseal region for vehicle versus E2 in Fig. 2f, and for sham versus OVX in Fig. 2k.

- It would strengthen the analysis to include bone formation rate (BFR) as part of the dynamic histomorphometric assessment presented in Fig. 5f.

- The study reports clear and consistent sex differences in the response to statins, with bone anabolic effects and enhanced osteogenic differentiation largely restricted to aged or estrogen-deficient female mice, while males appear largely unresponsive. While this is an important and biologically interesting observation, the manuscript currently treats these differences mainly as a consequence of estrogen deficiency without further exploration. It would strengthen the paper to expand the discussion of these sex-specific effects, including whether they are solely attributable to estrogen status or might also reflect intrinsic differences in LEPR$^+$ cell biology, OLN regulation, or statin responsiveness between sexes. Clarifying this point would also enhance the translational relevance of the findings.

- LEPR$^+$ bone marrow stromal cells represent a plausible primary source of circulating osteolectin, as has also been demonstrated previously. It would be important to consider whether circulating osteolectin serves a physiological role per se, or whether its presence in the bloodstream predominantly reflects local production within the bone marrow niche.

Reviewer #2

(Remarks to the Author)
The manuscript by Dr Yang and colleagues identifies a novel estrogen-deficiency-specific anabolic response to statins in bone; although statin effect on bone has been known for many years, the core findings of the manuscript show that estrogen and statins promote bone anabolism by converging on a shared osteogenic program in LEPR$^+$ marrow stromal cells through distinct upstream signaling mechanisms, marking a substantial step forward in the mechanistic explanation of the direct statin regulation of bone progenitor cells beyond cholesterol management and on the mechanism and cellular targets of estrogens action on bone.
The experimental design is impressive and fairly sophisticated, and the quality of the resulting data is excellent, making the

conclusions appropriate and well supported by mechanistic findings. Statistical approach is equally rigorous and appropriate.
A few concerns require further attention and some refinement in the discussion of findings:

1. While the authors' revision of the earliest epidemiological literature on the regulation of bone mass by statins is complete and accurate (Refs 4-8), the capacity of statins to promote bone anabolism has been questioned in more recent RCT and meta-analysis, potentially limiting the translational implication of this work (see doi: 10.1016/j.jamda.2019.06.027; doi:10.1001/jamainternmed.2014.6388). The authors are encouraged to discuss these more recent clinical data and to contextualize their findings with respect to the potential opportunities and limitations for future clinical translation.
2. Observational clinical data on human cohorts: what was the mean and median duration of statin therapy in both the Beijing and Suzhou cohorts prior to or during the tracking of annual BMD loss? Please add this important information to the description of the overall criteria followed to screen the population
3. Although the manuscript brilliantly identifies LEPR$^+$ cells as a site of estrogen/statin integration it does not address the question on which LEPR$^+$ subsets mediate the phenotype; the authors should more clearly acknowledge this limitation of their findings.
4. Exogenous E2 promotes osteogenic differentiation of LEPR$^+$ cells and increases bone mass in both male and female mice. However, the anabolic effects of statins appear restricted to estrogen-deficient cells or animals, including aged females and ovariectomized mice. Given that males possess the same LEPR$^+$ cell population and downstream signaling machinery, what accounts for the apparent sex- and endocrine-state specificity of the statin response? The authors are encouraged to discuss potential mechanisms underlying this context dependence.
5. Lines 151-152: it is unclear whether it was assumed that human cells express LEPR, whether the authors checked the osteoprogenitor cells for the expression of LEPR+ or whether cells were sorted based on LEPR expression. Please clarify.
6. Minor point: for the sake of clarity, it is suggested to add a line stating the years-after-menopause in Extended Data Figure 1a and 1b.

Reviewer #3

(Remarks to the Author)
This excellent study by Yang et al. delineates the mechanisms of statin-induced bone anabolism in estrogen deficiency with a focus on leptin receptor (LEPR)-expressing bone marrow stromal cells. The authors identify that estrogen primarily targets LEPR+ cells rather than osteoblasts/cytes and promotes the expression of the osteogenic growth factor osteolectin (OLN) through ER-alpha-mediated transcriptional activation. In estrogen-deficiency, statins elevate OLN expression by activating RAC1 by inhibiting prenylation via the mevalonate pathway, and promoting NF-kB-mediated OLN expression. The study, therefore, sheds light on an alternative statin-RAC1-NF-kB pathway that maintains OLN expression under estrogen deficiency.

The study is significant as it reexamines a long-standing controversial theory (statin-induced bone anabolism) using new and sophisticated approaches, including a new fate-mapping tool (Tet-On Lepr-Cre), reporter alleles (Esr1-GFP, Lepr-BFP, Oln-NanoLuc), conditional gene deletions (Esr1, Rac1), and CRISPR screening. The study is extremely well-designed, the data are very clearly presented, and the manuscript is carefully written. My enthusiasm for this study is extremely high. The following points can be considered to further strengthen the study.

1. Sexual dimorphism of OLN expression: The authors presented convincing data that OLN is a direct target of estrogen signaling in LEPR+ cells and mediates its anabolic actions. It is important to determine whether this machinery is specific to females and whether the backup pathway through RAC1, which statins tap into, is also present in males. The critical question is what drives OLN expression in males. Do male sex hormones, such as androgen, similarly drive OLN expression? Do Oln-/- mice show any sexual dimorphism in trabecular bone mass? Additionally, it is of great interest to address whether statins can also rescue osteoporosis caused by androgen deficiency. Augmenting the sexual dimorphism aspect will significantly strengthen the study.

2. Estrogen signaling in LEPR+ cells and bone resorption: It is surprising that bone resorption was not altered in Lepr-specific Esr1 mutant mice, as accelerated bone resorption is the hallmark of estrogen deficiency-induced osteoporosis. It is well established that LEPR+ cells abundantly express pro-osteoclastogenic factors, such as RANKL. Does Lepr-specific Esr1 deletion elevate RANKL expression? How does statin regulate RANKL expression in these cells? Describing in more detail the regulation of bone resorption by estrogen signaling and by statins will further enhance the study's impact.

3. Effects of statins on bone marrow adipocytes: Another hallmark of estrogen deficiency-induced osteoporosis is an increase in bone marrow adiposity. Considering that LEPR+ bone marrow stromal cells contain precursors for both osteoblasts and adipocytes, the authors should examine whether LEPR+ cells are biased toward adipocyte differentiation in the conditional mutant models (Esr1, Rac1), and whether statins can reverse adipocyte fates. It would be of great interest to examine if Oln-/- mice exhibit an increase in bone marrow adipocytes.

- Line 40: It is advised to avoid a double negative ("reduced BMD loss") to avoid confusion. It can be replaced by "higher BMD in postmenopausal women".
- Line 47: Based on the authors' findings, it might not be accurate to describe that estrogen and statin converge to promote bone anabolism, as statin does not have any effects in estrogen-sufficient subjects. Statin may activate a compensatory pathway that operates only when the primary estrogen-mediated pathway becomes dysfunctional.
- Line 73: Again, the authors should avoid a double negative where appropriate – higher BMD, instead of reduced BMD loss.

- Line 76: Fate mapping may be a more appropriate term than lineage tracing in this context.
- Line 114: The authors should clarify the regimen of atorvastatin administration, including the frequency, duration, and dose of the treatment. It is not described anywhere in the manuscript, including in the methods.
- Line 134: The increase in CTX-1 upon ovariectomy appears to be unusually modest. Do the authors have any speculation?
- Line 170: It is very interesting to see that E2 has a strong anabolic effect on male mice as well. Has this been noted previously?
- Line 182: Again, fate-mapping may be a more appropriate term.
- Line 206: The authors report that 85% of Esr1+ cells are LEPR+. Can the authors also show the percentage of Esr1+ cells among LEPR+ cells? It looks like almost 100%.
- Line 237-247: It is indeed a paradigm-shifting discovery that estrogen receptor in LEPR+ cells, but not osteoblasts/cytes, is what makes E2-induced anabolism possible. The authors undertook an admirable task of carefully dissecting the lineage requirement.
- Line 262: It should be noted and emphasized that OLN is not entirely responsible for E2-induced bone anabolism.
- Line 264: Sex of the CFU-F donor should be specified, both in mice and humans.
- Line 285: The authors should note that the Esr1-independent decline in bone mass is more significant than the Esr1-dependent one.
- Line 295: The statement that the observed osteopenia is driven by impaired bone formation rather than increased resorption is interesting. Do the authors postulate that estrogen's direct action on osteoclasts is responsible for enhanced bone resorption in estrogen deficiency, rather than an indirect mechanism through the RANKL-RANK system?
- Line 309: How does a circulating OLN look in male patients as well?
- Figure 4h: Can the authors also show the graph with the male and female data separately?
- Figure 7e: The authors should clarify that these mice were ovariectomized.

Reviewer #4

(Remarks to the Author)
Yang, Guo, Ye et al present a thorough examination of statin effects on bone mass using both human and mouse models. The data on the effects of statins on BMD and fracture risk in humans is mixed, but they generally suggest there is a positive effect in at least some settings. These authors show that statins improve bone mass in mice, but only in settings of estrogen deficiency, and in two fairly small cohorts of postmenopausal women. They demonstrate that both statins and estrogen can promote osteogenic differentiation of LepR+ progenitor cells, but, somewhat surprisingly, the statins do not appear to affect osteoclast activity. They go on to show that ERalpha expression is enriched in LepR+ cells, and propose that osteolectin is an important modulator of both estrogen and statin activity, either through direct activation, or through Rac1-mediated NFkB signaling. This study is novel with robust approaches and the use of several novel mouse lines. Statistics appear appropriate for the most part, and while some results are overstated, the overall conclusions are appropriate.

Major comments
1. There are a lot of methodological details that are important to understand the results that are not covered in the results or figures. Figure 2 in particular is hard to understand, but in many cases for mouse studies it would be helpful to add an experimental outline diagram so it is clear when treatments and/or surgery are performed in relation to each other, or at least state this in the figure legend (such as timing of doxycycline, atorvastatin etc).
2. The clinical data shown in Fig 1 should be introduced as a retrospective study. It is surprising that the control women in this age group do not really appear to be losing BMD. Other studies in this age group typically show some annual decrease (about 1% per year in
doi: 10.1056/NEJMoa2407031). Is data from other sites than lumbar spine also available? The violin plots give a nice indication of the spread of the data, but the authors could consider showing the change per year as a %, and adding both change/year and % change to the table in the extended data.
3. ssRNAseq analysis of bone marrow stromal populations have indicated some diversity in LepR+ cells, and suggest that they include both adipo-CAR (likely in the central marrow) and osteo-CAR (closer to the bone surface) cells. Can the authors reanalyze data from other studies to determine which cell subsets express osteolectin? The authors should at least acknowledge that the osteogenic effect of these treatments may be primarily on the more bone-adjacent cell populations rather than the central marrow cells that do not typically become osteoblasts unless there is an injury or other major perturbation (e.g. DOI: 10.1038/s41467-019-14029-w).
4. The effect of statin treatment and estrogen deficienty in mice on osteoclasts is only ever examined using CTX, which shows a relatively small change with OVX (Fig 1k). Given that the prenylation pathway is a well-established regulator of osteoclast activity, the authors should confirm if there are differences in trabecular or endocortical osteoclast numbers in these animals, and the conditional receptor knockout animals in Figure 5.
5. The data showing enhanced in vitro differentiation in response to statins is insufficient (Fig 1l-o). Additional verification of enhanced osteogenic differentiation such as expression of marker genes should be shown, and ideally the mouse studies should be done in males and females.
6. I want to commend the authors for making a the tet-on LepR-Cre for this study. However can they please include some type of data to evaluate the recombination efficiency, and some idea of how well it recapitulates LepR-CreER. The % of osteoblasts labeled in Figure 2 sounds lower than the previous reports, although determining the timeframe for these studies is not straightforward (see comment 1).
7. For the studies looking at LepR-Tomato contribution to GFP+ osteoblasts in Figure 2, quantification of trabecular and endocortical surfaces should also be performed using the histology as these compartments can have different responses to estrogen deficiency and contributions of LepR cells.
8. Why do the CUT&Tag studies in Figure 4 lack an untreated control? ERalpha may be able to perform ligand independent binding to some sites.

Minor comments
1. CFU-F is used to describe many of the culture experiments, and while it sounds like they are grown initially as CFU-F, they are then pooled and reseeded for many of the assays. Perhaps the authors could rename to passage 1 bone marrow CFU-F or something like that.
2. Line 126: this title does not accurately describe the results in this section.
3. Line 176-177 (and several other places): Almeida et al 2013 (and a review by similar authors in 2017) suggests that a lot of the activity of estrogen on osteogenesis is targeting earlier stages of the lineage since they see essentially no phenotype in their mature osteoblast model. Please tone down these statements.
4. Extended Figure 4, Extended Figure 6 and associated results: can further cortical parameters be shown such as the perimeter, or the total area and marrow area for all these studies. It would be useful to understand how much the estrogen-related changes are mediated by periosteal vs endosteal expansion.
5. The official gene symbol for osteolectin should be mentioned when it is introduced
6. Line 260-261/interpretation of Figure 4h: this knockout mouse clearly has a pre-existing phenotype. They still have a substantial response to estrogen, so it is difficult to justify this statement
7. Osteolectin levels in different conditions: for extended Fig 7c, can plasma levels from the sham group be included for reference?
8. Methods: For the retrospective human study, please include additional information about how the participants were chosen, the beginning and end dates of the study cohort data, and the maximum amount of time between Dexa scans.
9. Methods: The human studies with serum collection appear to be different patients than for the dexa study, please state how these patients were recruited, and if there were any differences in inclusion/exclusion criteria.
10. Methods: how the human marrow samples were collected and cultured is not covered, not are any of the demographics of these patients.
11. Methods line 864: please elaborate on what 'freshly dissociated' means, is this flushed marrow, or enzymatically dissociated marrow/endosteum?
12. Methods: flow cytometry is described for flushed bone marrow, but how were samples where Col1a1-GFP was analyzed prepared?
13. Figure 1a-b and Fig 6a-b - please include the n for each group in either the figure or the legend.
14. Figure 4f-g data: can the more extended list of differentially expressed genes be provided as supplemental data. Are Rankl and Opg regulated as would be expected from previous reports?
15. Figure 4i-j and 7h-l (cut and tag data) - can some sort of scale be included on both axes of these plots.
16. Figure 7 western blots: quantification should be included somewhere for these. For b in particular the difference is subtle.
17. Ref 48 should be PubMed ID:11771662

References
A lot of the references are quite old, particularly the clinical references, which partly reflects that the high quality work in this field was mostly published some time ago. However, with regards to the clinical evidence, there are several meta analyses, at least one of which would be appropriate to cite.

Version 1:

Reviewer comments:

Reviewer #1

(Remarks to the Author)
Thank the authors for their efforts to address my comments. I have no further comments and recommend acceptance of the manuscript.

Reviewer #2

(Remarks to the Author)
The authors have satisfactorily addressed my concerns and have improved the manuscript as requested

Reviewer #3

(Remarks to the Author)
It is admirable that the authors returned such an extensively revised manuscript in a very short amount of time. In response to my critiques, the authors provided several important pieces of supporting evidence for the revised manuscript, including 1) a lack of statin-induced anabolic responses in bone in orchiectomized males, 2) unaltered RANKL and OPG in Esr1-deficient LepR+ cells, and 3) increased bone marrow adipocytes in estrogen-deficient mice rescued by statins. Overall, this remains an extremely well-conducted study demonstrating the compensatory actions of statins in estrogen-deficient LepR+ cells. A few minor suggestions are listed below.

1. Abstract, Line 40-42. The authors should clarify that atorvastatin's anabolic effect can only be observed in estrogen deficiency.
2. Abstract, Line 45. As I pointed out in the initial round of peer review, it is not entirely accurate that estrogen and statins "converge" to promote bone anabolism. A more accurate description would be that statins "compensate" for the signaling

defect induced by estrogen deficiency through mechanistically distinct routes.
3. Additional results on bone marrow adipocytes are greatly appreciated. It would be important to show the images of bone marrow adipocytes in each group. How was the adipocyte number determined? Did the authors use H&E or immunostaining?

Reviewer #4

(Remarks to the Author)
The authors have done a good job addressing most comments. Addition of the data establishing efficiency of the Tet-On LepR model is a really helpful addition technically, and as the authors point out, hints at confounding results from the use of tamoxifen.

Two of my comments were not fully addressed as outlined below, which could be rectified by minor changes to text and/or figures.

Major comment 1
The authors provided a little more detail in the methods section, but not a diagram in the figures or outline of the study design in the figure legend which was what was requested. It would be good to include this at least for Figure 2.
Major comment 7
Thank you for including the histological evaluation, but the results should mention the compartment-specific data in the text at least for Fig S3e-f where the effect on trabecular bone predominates.

Open Access This Peer Review File is licensed under a Creative Commons Attribution 4.0 International License, which permits use, sharing, adaptation, distribution and reproduction in any medium or format, as long as you give appropriate credit to the original author(s) and the source, provide a link to the Creative Commons license, and indicate if changes were made.
In cases where reviewers are anonymous, credit should be given to 'Anonymous Referee' and the source.
The images or other third party material in this Peer Review File are included in the article's Creative Commons license, unless indicated otherwise in a credit line to the material. If material is not included in the article's Creative Commons license and your intended use is not permitted by statutory regulation or exceeds the permitted use, you will need to obtain permission directly from the copyright holder.
To view a copy of this license, visit https://creativecommons.org/licenses/by/4.0/

June 25, 2026

Qingzhong Ren, PhD
Senior Editor, Nature Aging

Dear Qingzhong,

Thank you to the reviewers for reading our paper and providing thoughtful comments. We have added new data, clarifications, and revisions that address all of the reviewer comments. Below is a point-by-point response in which reviewer comments are presented in full in *italics* followed by our responses in normal type.

Referee #1:

This is a well-conducted and timely study addressing an important clinical and biological question, namely the link between statin use and bone mineral density in estrogen-deficient conditions. The integration of human cohort data with mechanistic investigations in mouse models and cellular systems is a clear strength and provides a compelling framework for the proposed mechanism involving osteolectin. I also appreciate the relatively concise discussion, which is appropriate given that the results are clear and directly support the main conclusions. Overall, the manuscript is of broad interest and provides valuable insight. I suggest the following comments to further strengthen clarity and interpretation:

The manuscript states that three or four independent experiments were performed for most animal studies. It would be important to clarify how these experiments were conducted, how variability between them was addressed, and on what basis one dataset was chosen as representative.

RESPONSE: We thank the reviewer for this helpful suggestion. We have clarified that independent animal experiments corresponded to separate cohorts of mice generated, treated, and analyzed at different times using the same experimental protocol. Data from all animals in these independent experiments were pooled for statistical analysis. To make this transparent, we have added the number of animals contributed by each independent experiment to the Source Data tables.

We have also clarified how representative images and blots were selected. For immunofluorescence images, representative panels were selected from samples with technically comparable staining quality, matched anatomical location, and staining patterns consistent with the group-level results observed across independent experiments. For immunoblots, representative blots were selected from experiments showing the reproducible banding pattern and direction of effect observed across biological replicates. In accordance with Nature Portfolio requirements, we have provided uncropped images for all replicate immunoblots in the Source Data files.

Figures 2f and 2k: Please ensure that the representative images display trabecular bone from the same anatomical location within the metaphyseal region for vehicle versus E2 in Fig. 2f, and for sham versus OVX in Fig. 2k.

RESPONSE: We thank the reviewer for pointing this out. We have re-examined the representative immunofluorescence images shown in Fig. 2f and Fig. 2k and re-selected the higher-magnification images from comparable trabecular regions within the distal femoral metaphysis for each comparison. Specifically, the trabecular areas shown in the vehicle and E2 images in Fig. 2f, as well as in the sham and OVX images in Fig. 2k, were selected from matched anatomical locations with similar orientation and magnification.

It would strengthen the analysis to include bone formation rate (BFR) as part of the dynamic histomorphometric assessment presented in Fig. 5f.

RESPONSE: We thank the reviewer for this helpful suggestion. We have now added bone formation rate (BFR) measurements in new Fig. 5g to complement the mineral apposition rate (MAR) analysis shown in Fig. 5f. BFR was calculated from the same dynamic histomorphometric calcein/Alizarin Red double-labeling analyses used for MAR quantification. Consistent with the reduced MAR and decreased plasma P1NP levels, BFR was reduced following *Esr1* deletion in LEPR^{+} cells, further supporting the conclusion that the accelerated bone loss is driven by impaired bone formation. We have revised the Results, Methods, and figure legend accordingly.

The study reports clear and consistent sex differences in the response to statins, with bone anabolic effects and enhanced osteogenic differentiation largely restricted to aged or estrogen-deficient female mice, while males appear largely unresponsive. While this is an important and biologically interesting observation, the manuscript currently treats these differences mainly as a consequence of estrogen deficiency without further exploration. It would strengthen the paper to expand the discussion of these sex-specific effects, including whether they are solely attributable to estrogen status or might also reflect intrinsic differences in LEPR^{+} cell biology, OLN regulation, or statin responsiveness between sexes. Clarifying this point would also enhance the translational relevance of the findings.

RESPONSE: We thank the reviewer for this important and constructive suggestion. We agree that the sex-specific response to statins should not be interpreted as a simple consequence of estrogen deficiency alone. We have revised the Discussion to clarify that our data support estrogen deficiency as a permissive endocrine context for atorvastatin-induced bone anabolism, rather than as the sole determinant of responsiveness. Specifically, atorvastatin increased *Oln* expression, osteogenic differentiation of LEPR^{+} cells, and bone mass in aged or ovariectomized female mice. By contrast, aged males, young adult mice, and orchiectomized males did not exhibit a comparable bone anabolic response. These observations, together with the new data showing that androgen treatment did not detectably increase *Oln* transcription in CFU-Fs (new Extended Data Fig. 5o), and that atorvastatin did not increase bone mass in orchiectomized males (new Extended Data Fig. 2e−h), indicate

that atorvastatin-induced bone anabolism is specifically associated with estrogen deficiency in females, whereas androgen deficiency in males does not confer a comparable response. We have also added a brief statement to the Discussion noting that sex-dependent differences in LEPR$^+$ cells or OLN regulation may also contribute to this phenotype. These revisions clarify that the skeletal effects of statins may vary with sex, age, and endocrine status, and therefore may not occur uniformly across all physiological or clinical contexts.

LEPR$^+$ bone marrow stromal cells represent a plausible primary source of circulating osteolectin, as has also been demonstrated previously. It would be important to consider whether circulating osteolectin serves a physiological role per se, or whether its presence in the bloodstream predominantly reflects local production within the bone marrow niche.

RESPONSE: We thank the reviewer for this helpful comment. We agree that LEPR$^+$ cells represent a plausible source of circulating OLN, consistent with previous work showing that OLN is a secreted osteogenic factor produced by LEPR$^+$ cells and that it promotes osteogenic differentiation of these cells. In our study, statins increased *Oln* transcription in LEPR$^+$ cells and increased circulating OLN levels in postmenopausal women, ovariectomized mice, and aged female mice. Together with the loss of statin-induced bone anabolism in *Oln*$^{-/-}$ mice, these findings support a model in which atorvastatin promotes OLN production by LEPR$^+$ cells in the bone marrow. We agree that the current study does not determine whether circulating OLN itself has a physiological endocrine role. We therefore interpret circulating OLN as a systemic correlate of OLN induction, whereas the functional data support a role for OLN in the local bone marrow environment. Whether circulating OLN also has an endocrine function remains an interesting question for future studies.

Referee #2:

The manuscript by Dr Yang and colleagues identifies a novel estrogen-deficiency-specific anabolic response to statins in bone; although statin effect on bone has been known for many years, the core findings of the manuscript show that estrogen and statins promote bone anabolism by converging on a shared osteogenic program in LEPR$^+$ marrow stromal cells through distinct upstream signaling mechanisms, marking a substantial step forward in the mechanistic explanation of the direct statin regulation of bone progenitor cells beyond cholesterol management and on the mechanism and cellular targets of estrogens action on bone.

The experimental design is impressive and fairly sophisticated, and the quality of the resulting data is excellent, making the conclusions appropriate and well supported by mechanistic findings. Statistical approach is equally rigorous and appropriate.
A few concerns require further attention and some refinement in the discussion of findings:

1. While the authors' revision of the earliest epidemiological literature on the regulation of bone mass by statins is complete and accurate (Refs 4-8), the capacity of statins to promote bone anabolism has been questioned in more recent RCT and meta-analysis, potentially

limiting the translational implication of this work (see doi: 10.1016/j.jamda.2019.06.027; doi:10.1001/jamainternmed.2014.6388). The authors are encouraged to discuss these more recent clinical data and to contextualize their findings with respect to the potential opportunities and limitations for future clinical translation.

RESPONSE: We thank the reviewer for raising this important point. We agree that more recent clinical evidence has tempered the interpretation of earlier observational associations between statin use and skeletal outcomes. We have now cited and discussed the JUPITER randomized clinical trial (JAMA Intern Med 175:171), in which rosuvastatin did not reduce incident fracture risk, and the updated meta-analysis (J Am Med Dir Assoc 20:1566), which found that observational studies suggested an association between statin use and reduced fracture risk, whereas randomized trial data did not support a significant fracture-preventive effect. We have added a concise discussion noting that the context dependence observed in our study may help explain why clinical studies of statins and skeletal outcomes have yielded variable results, with observational associations in some cohorts but no consistent fracture-preventive effect in randomized or meta-analytic studies. Our study was not designed to establish that statins reduce fracture risk or should be used as osteoporosis therapies. Rather, our clinical analysis focused on annualized lumbar spine BMD loss in postmenopausal women, and our mechanistic studies identify estrogen deficiency as a permissive context in which atorvastatin promotes bone formation through $LEPR^+$ cells.

2. Observational clinical data on human cohorts: what was the mean and median duration of statin therapy in both the Beijing and Suzhou cohorts prior to or during the tracking of annual BMD loss? Please add this important information to the description of the overall criteria followed to screen the population

RESPONSE: We thank the reviewer for this helpful suggestion. We have now added the duration of lipid-lowering therapy in both clinical cohorts. In our retrospective analysis, medication exposure was defined as continuous use of statin or evolocumab throughout the interval between the paired DXA scans used to calculate annualized BMD change. The mean and median duration of therapy for statin users and evolocumab users are now included in the revised Extended Data Fig. 1a, b; control participants had no lipid-lowering therapy. We have also clarified the screening criteria for the retrospective cohort analysis, including how medication exposure was defined relative to the DXA measurement interval. In addition, we have added menopause age, years since menopause, DXA interval, annual absolute BMD change, and annual percentage BMD change to Extended Data Fig. 1a, b. Age, menopause age, years since menopause, DXA interval, and other baseline variables were comparable among control participants, evolocumab users, and statin users in both cohorts. These additions clarify the duration of medication exposure and the comparability of menopause-related baseline variables across groups.

3. Although the manuscript brilliantly identifies $LEPR^+$ cells as a site of estrogen/statin integration it does not address the question on which $LEPR^+$ subsets mediate the phenotype; the authors should more clearly acknowledge this limitation of their findings.

RESPONSE: We thank the reviewer for raising this important point. We agree that LEPR⁺ cells are heterogeneous and that our current genetic and fate-mapping approaches do not resolve which LEPR⁺ subsets mediate the estrogen- and statin-associated osteogenic response. To provide additional context, we re-analyzed a previously published single-cell RNA-seq dataset of bone marrow stromal populations (Nature 569:222). This analysis showed that *Esr1*, *Oln*, *Sp7* and *Bglap* were expressed at higher levels in an overlapping LEPR⁺ cell subset (new Extended Data Fig. 5k–n). These data suggest that *Esr1* and *Oln* expression is not uniform across all LEPR⁺ cells and is associated with a LEPR⁺ cell subset with osteogenic features. We have revised the Discussion to explicitly acknowledge that our current genetic approaches targeting LEPR⁺ cells do not determine whether estrogen and statins act broadly across this population or preferentially through a more restricted subset. Because OLN is secreted, the OLN-producing and OLN-responsive cells may not be identical. Future subset-specific genetic approaches targeting distinct LEPR⁺ subsets will be needed to test whether particular LEPR⁺ subpopulations preferentially mediate these responses.

4. Exogenous E2 promotes osteogenic differentiation of LEPR⁺ cells and increases bone mass in both male and female mice. However, the anabolic effects of statins appear restricted to estrogen-deficient cells or animals, including aged females and ovariectomized mice. Given that males possess the same LEPR⁺ cell population and downstream signaling machinery, what accounts for the apparent sex- and endocrine-state specificity of the statin response? The authors are encouraged to discuss potential mechanisms underlying this context dependence.

RESPONSE: We thank the reviewer for raising this important point. We agree that the sex- and endocrine-state specificity of the statin response is an important feature of the study, and we have added a brief discussion of this context dependence. Our data indicate that statin-induced bone anabolism is associated with estrogen deficiency in females. Atorvastatin increased *Oln* expression, osteogenic differentiation of LEPR⁺ cells, and bone mass in ovariectomized and aged female mice, but not in aged males, young adult mice, or orchiectomized males. Consistent with this context dependence, atorvastatin increased *Oln* expression in LEPR⁺ cells and circulating OLN levels in aged females, but not in aged males. By contrast, exogenous E2 promoted osteogenic differentiation of LEPR⁺ cells and increased bone mass in both sexes, indicating that male LEPR⁺ cells retain the capacity to respond to direct E2–ERα signaling.

We do not view these findings as contradictory, because exogenous E2 and atorvastatin act through different upstream mechanisms. E2 directly activates ERα signaling, whereas atorvastatin induces *Oln* transcription through inhibition of the mevalonate pathway, reduced RAC1 prenylation, and NF-κB activation. Thus, male LEPR⁺ cells can respond to E2–ERα activation, but this does not necessarily mean that the indirect statin-mediated pathway is similarly induced in males. Our new data further support this distinction: androgen treatment did not detectably increase *Oln* transcription in CFU-Fs (new Extended Data Figure 5o), and atorvastatin did not increase bone mass in orchiectomized males (new Extended Data Figure

2e−h). These findings indicate that androgen deficiency does not produce a comparable statin response. We now state in the Discussion that estrogen deficiency in females appears to provide a permissive endocrine context for this response, although sex-dependent differences in LEPR$^+$ cells or OLN regulation may also contribute.

5. Lines 151-152: it is unclear whether it was assumed that human cells express LEPR, whether the authors checked the osteoprogenitor cells for the expression of LEPR+ or whether cells were sorted based on LEPR expression. Please clarify.

RESPONSE: We thank the reviewer for pointing out this ambiguity. We have revised the Results and figure legend to clarify the relationship between human CFU-F cultures and LEPR expression. The human osteogenic differentiation assays were performed using CFU-Fs established from enzymatically dissociated human bone marrow cells under CFU-F culture conditions. To determine whether CFU-F activity in human bone marrow is associated with the LEPR$^+$ cell population, we performed a separate prospective sorting experiment. Human bone marrow cells were sorted by flow cytometry, and CFU-F activity was detected predominantly in the LEPR$^+$ fraction, whereas unfractionated whole bone marrow generated few colonies under the same culture conditions (new Extended Data Fig. 2n). These data show that human CFU-F activity is enriched in LEPR$^+$ cells, supporting the connection between the human CFU-F assays and the LEPR$^+$ cell population. We have revised the text to state this more precisely and have avoided implying that LEPR expression alone fully defines human skeletal stem/progenitor identity.

6. Minor point: for the sake of clarity, it is suggested to add a line stating the years-after-menopause in Extended Data Figure 1a and 1b.

RESPONSE: We thank the reviewer for this suggestion. We have now added years since menopause to the baseline characteristics shown in Extended Data Fig. 1a, b for both clinical cohorts and clarified this variable in the Methods section describing the retrospective cohort analysis.

<u>Referee #3</u>:

This excellent study by Yang et al. delineates the mechanisms of statin-induced bone anabolism in estrogen deficiency with a focus on leptin receptor (LEPR)-expressing bone marrow stromal cells. The authors identify that estrogen primarily targets LEPR+ cells rather than osteoblasts/cytes and promotes the expression of the osteogenic growth factor osteolectin (OLN) through ER-alpha-mediated transcriptional activation. In estrogen-deficiency, statins elevate OLN expression by activating RAC1 by inhibiting prenylation via the mevalonate pathway, and promoting NF-kB-mediated OLN expression. The study, therefore, sheds light on an alternative statin-RAC1-NF-kB pathway that maintains OLN expression under estrogen deficiency.

The study is significant as it reexamines a long-standing controversial theory (statin-induced bone anabolism) using new and sophisticated approaches, including a new fate-mapping tool (Tet-On Lepr-Cre), reporter alleles (Esr1-GFP, Lepr-BFP, Oln-NanoLuc), conditional gene deletions (Esr1, Rac1), and CRISPR screening. The study is extremely well-designed, the data are very clearly presented, and the manuscript is carefully written. My enthusiasm for this study is extremely high. The following points can be considered to further strengthen the study.

1. Sexual dimorphism of OLN expression: The authors presented convincing data that OLN is a direct target of estrogen signaling in LEPR+ cells and mediates its anabolic actions. It is important to determine whether this machinery is specific to females and whether the backup pathway through RAC1, which statins tap into, is also present in males. The critical question is what drives OLN expression in males. Do male sex hormones, such as androgen, similarly drive OLN expression? Do Oln-/- mice show any sexual dimorphism in trabecular bone mass? Additionally, it is of great interest to address whether statins can also rescue osteoporosis caused by androgen deficiency. Augmenting the sexual dimorphism aspect will significantly strengthen the study.

RESPONSE: We thank the reviewer for raising this important point. We agree that the sex specificity of OLN regulation and the statin response is an important aspect of the study. To address whether androgen signaling regulates *Oln* transcription in a manner similar to E2, we treated CFU-Fs with dihydrotestosterone and measured *Oln* transcript levels. In contrast to E2, dihydrotestosterone did not detectably increase *Oln* transcription under these conditions (new Extended Data Fig. 5o). These data suggest that androgen signaling does not substitute for E2 in inducing *Oln* expression. We next tested whether androgen deficiency, like estrogen deficiency, allows statins to increase bone mass. Adult male mice were subjected to orchiectomy (ORX), a model of androgen-deficiency-induced bone loss, and were then treated with atorvastatin or vehicle. In contrast to ovariectomized female mice, atorvastatin did not increase bone mass in orchiectomized male mice compared with vehicle-treated controls (new Extended Data Fig. 2e–h). Consistent with this result, 18-month-old male mice also did not show the statin-induced bone anabolic response observed in age-matched females. These findings indicate that androgen deficiency in males does not confer a comparable response to atorvastatin.

We also analyzed male and female *Oln*$^{-/-}$ mice separately to determine whether the skeletal requirement for OLN differs by sex. *Oln* deficiency caused a more pronounced reduction in bone mass in females than in males (new Extended Data Fig. 6a–d), suggesting that the female skeleton is more sensitive to loss of OLN. Together with our finding that atorvastatin increased *Oln* transcription in LEPR^{+} cells in aged females but not in aged males, these new data support sex-dependent differences in OLN regulation and in the skeletal response to atorvastatin.

We have revised the Results to incorporate these new experiments and added a brief statement to the Discussion. We now clarify that, although male LEPR^{+} cells retain the ability

to respond to direct E2–ERα signaling, as shown by the anabolic response to exogenous E2, this does not necessarily mean that the indirect statin-regulated pathway is engaged similarly in males. Our data do not identify the mechanisms that maintain basal *Oln* expression in males, and they do not exclude sex-dependent differences in $LEPR^+$ cell biology, basal RAC1 prenylation, NF-κB responsiveness, or other aspects of statin responsiveness. These remain important questions for future study.

2. Estrogen signaling in LEPR+ cells and bone resorption: It is surprising that bone resorption was not altered in Lepr-specific Esr1 mutant mice, as accelerated bone resorption is the hallmark of estrogen deficiency-induced osteoporosis. It is well established that LEPR+ cells abundantly express pro-osteoclastogenic factors, such as RANKL. Does Lepr-specific Esr1 deletion elevate RANKL expression? How does statin regulate RANKL expression in these cells? Describing in more detail the regulation of bone resorption by estrogen signaling and by statins will further enhance the study's impact.

RESPONSE: We thank the reviewer for raising this important point. We agree that, because $LEPR^+$ cells can express pro-osteoclastogenic factors such as RANKL, it was important to determine whether ERα loss or statin treatment altered osteoclastogenic signals from these cells. We therefore analyzed *Rankl* and *Opg* expression in sorted $LEPR^+$ cells. *Esr1* deletion in $LEPR^+$ cells did not significantly increase *Rankl* expression or reduce *Opg* expression in $LEPR^+$ cells (new Extended Data Fig. 8e, f). Similarly, atorvastatin treatment in ovariectomized mice did not significantly alter *Rankl* or *Opg* expression in $LEPR^+$ cells (new Extended Data Fig. 2i, j). We also performed additional osteoclast analyses in the relevant models. Quantification of osteoclast number on trabecular bone surfaces showed that *Esr1* deletion in $LEPR^+$ cells did not significantly increase osteoclast number relative to controls (new Extended Data Fig. 8g), and atorvastatin did not significantly reduce osteoclast number in ovariectomized mice (new Extended Data Fig. 2k). These results are consistent with the serum CTX-1 measurements and indicate that the skeletal phenotypes examined here are not associated with detectable changes in *Rankl* and *Opg* expression in $LEPR^+$ cells, osteoclast number, or systemic bone resorption markers.

We have revised the Results to incorporate these findings, showing that in the models examined here, ERα signaling in $LEPR^+$ cells and atorvastatin treatment mainly affect the bone-formation phenotype, without detectable changes in the resorption parameters we measured. We also clarify that our conclusion refers specifically to *Esr1* deletion in $LEPR^+$ cells and statin-treated models analyzed in this study, rather than to osteoporosis caused by systemic estrogen deficiency in general. Systemic estrogen deficiency may increase bone resorption through mechanisms outside the $LEPR^+$ cell population, including osteoclast-lineage cells or other estrogen-responsive cell types. Thus, our data do not exclude established mechanisms by which estrogen regulates osteoclast function, but indicate that altered osteoclastogenesis or bone resorption is unlikely to be the major mechanism underlying the phenotypes observed in the $LEPR^+$ cell-specific knockout and statin-treated models analyzed in this study.

3. Effects of statins on bone marrow adipocytes: Another hallmark of estrogen deficiency-induced osteoporosis is an increase in bone marrow adiposity. Considering that LEPR+ bone marrow stromal cells contain precursors for both osteoblasts and adipocytes, the authors should examine whether LEPR+ cells are biased toward adipocyte differentiation in the conditional mutant models (Esr1, Rac1), and whether statins can reverse adipocyte fates. It would be of great interest to examine if Oln-/- mice exhibit an increase in bone marrow adipocytes.

RESPONSE: We thank the reviewer for this important suggestion. We agree that bone marrow adiposity is an important component of estrogen-deficiency-associated bone loss and that LEPR$^+$ cells include progenitors with both osteogenic and adipogenic potential. We have therefore added new analyses of marrow adipocyte accumulation in the relevant genetic and pharmacological models. *Esr1* deletion in LEPR$^+$ cells increased marrow adipocyte numbers compared with littermate controls (new Extended Data Fig. 8h), indicating that impaired ERα signaling in LEPR$^+$ cells is associated with increased adipocyte accumulation as well as reduced osteogenic differentiation. We further examined whether atorvastatin counteracts the increased marrow adipocyte accumulation associated with estrogen deficiency. OVX increased bone marrow adipocyte number, whereas atorvastatin treatment attenuated this increase (new Extended Data Fig. 2l), consistent with atorvastatin suppressing the increased adipogenic differentiation of LEPR$^+$ cells while promoting their osteogenic differentiation under estrogen-deficient conditions.

We next asked whether this effect of atorvastatin required RAC1 in LEPR$^+$ cells. In *Rac1* cKO mice, atorvastatin no longer increased bone mass under estrogen-deficient conditions. Atorvastatin also did not reduce bone marrow adipocyte number in these mice (new Extended Data Fig. 10k). These findings indicate that RAC1 is required for the effects of atorvastatin on bone anabolism and marrow adipocyte accumulation in estrogen-deficient mice.

Finally, previous work showed that *Oln*$^{-/-}$ mice exhibit increased bone marrow adiposity (eLife 5:e18782). This finding is consistent with our model that OLN promotes the osteogenic differentiation of LEPR$^+$ cells and that reduced OLN activity is associated with increased marrow adipocyte accumulation. We have revised the Results to incorporate the new adipocyte analyses in the *Esr1* and *Rac1* conditional knockout models. Together with the previously reported increase in marrow adiposity in *Oln*$^{-/-}$ mice, these findings support an opposing relationship between osteogenic differentiation and marrow adipocyte accumulation in these models.

- Line 40: It is advised to avoid a double negative ("reduced BMD loss") to avoid confusion. It can be replaced by "higher BMD in postmenopausal women".

RESPONSE: We thank the reviewer for this helpful suggestion. Because our clinical analysis assessed annualized BMD change rather than cross-sectional BMD, we did not replace these statements with "higher BMD" when referring to our cohort data. Instead, we revised

the relevant text to avoid the double-negative phrasing and now describe the finding as a “slower annual decline in BMD” or “attenuated annual BMD loss,” as appropriate.

- Line 47: Based on the authors’ findings, it might not be accurate to describe that estrogen and statin converge to promote bone anabolism, as statin does not have any effects in estrogen-sufficient subjects. Statin may activate a compensatory pathway that operates only when the primary estrogen-mediated pathway becomes dysfunctional.

RESPONSE: We agree with the reviewer that the original wording could be interpreted as implying that statins promote bone anabolism under estrogen-sufficient conditions. We have revised the text to clarify the intended meaning. Specifically, “converge” refers to molecular convergence on *Oln*/*OLN* regulation in $LEPR^+$ cells, rather than equivalent anabolic effects across endocrine states. Our data indicate that E2 and statins promote *Oln* transcription through distinct upstream mechanisms: E2 acts through ERα, whereas statins engage the RAC1–NF-κB pathway. We have revised the Abstract and related text to clarify that the statin-induced bone anabolism is observed under estrogen-deficient conditions.

- Line 73: Again, the authors should avoid a double negative where appropriate – higher BMD, instead of reduced BMD loss.

RESPONSE: We thank the reviewer for this helpful suggestion. Because our clinical analysis assessed annualized BMD change rather than cross-sectional BMD alone, we did not replace these statements with “higher BMD” when referring to our cohort data. Instead, we revised the relevant text to avoid double-negative phrasing where possible and now describe the finding as a “slower annual decline in BMD” or “attenuated annual BMD loss,” as appropriate.

- Line 76: Fate mapping may be a more appropriate term than lineage tracing in this context.

RESPONSE: We agree with the reviewer and have revised the text to use “fate mapping” where referring to the contribution of $LEPR^+$ cells to osteoblasts and osteocytes.

- Line 114: The authors should clarify the regimen of atorvastatin administration, including the frequency, duration, and dose of the treatment. It is not described anywhere in the manuscript, including in the methods.

RESPONSE: We have now added the atorvastatin administration regimen, including dose, route, frequency, and duration, to the Methods section and clarified treatment timing in the relevant figure legends.

- Line 134: The increase in CTX-1 upon ovariectomy appears to be unusually modest. Do the authors have any speculation?

RESPONSE: We thank the reviewer for this comment. We agree that the increase in serum CTX-1 after ovariectomy was modest in our experiment. We speculate that this may reflect the timing and conditions of sample collection. CTX-1 is a systemic and time-sensitive marker of bone resorption, and its magnitude can be influenced by the interval after OVX, mouse age, fasting status, circadian timing, and assay conditions (Bone 29:361, Bone 31:57, Clin Biochem 45:928, Osteoporos Int 28:2541). It is also possible that the peak increase in bone resorption occurred earlier after OVX than the time point at which serum was collected in our study. To strengthen the assessment of bone resorption, we have now added quantification of osteoclast numbers on trabecular bone surfaces in ovariectomized mice (new Extended Data Fig. 2k). Consistent with the CTX-1 measurements, atorvastatin did not significantly reduce osteoclast numbers in OVX mice. Together with the increased bone formation and LEPR$^+$ cell osteogenic differentiation observed after atorvastatin treatment, these data support the conclusion that the statin-induced increase in bone mass in ovariectomized mice is a result of enhanced bone formation rather than a reduction in bone resorption.

- Line 170: It is very interesting to see that E2 has a strong anabolic effect on male mice as well. Has this been noted previously?

RESPONSE: We thank the reviewer for this comment. Yes, the anabolic effect of exogenous E2 in male mice has been noted previously (Bone Res. 12:1 and PNAS 100:13573). Our observation that exogenous E2 increases bone mass in males is consistent with these prior reports. In the context of our study, this finding is important because it indicates that male LEPR$^+$ cells retain the capacity to respond to direct E2–ERα signaling. However, this does not imply that males respond similarly to statins, because atorvastatin engages an indirect mevalonate–RAC1–NF-κB pathway to induce *Oln* expression. Consistent with this distinction, aged males and orchiectomized males did not exhibit the statin-induced anabolic response observed in aged or ovariectomized females.

- Line 182: Again, fate-mapping may be a more appropriate term.

RESPONSE: We agree with the reviewer and have replaced “lineage tracing” with “fate mapping”.

- Line 206: The authors report that 85% of Esr1+ cells are LEPR+. Can the authors also show the percentage of Esr1+ cells among LEPR+ cells? It looks like almost 100%.

RESPONSE: We thank the reviewer for this suggestion. We have now added quantification showing that 81 ± 9.4% of LEPR$^+$ cells express *Esr1*-GFP (new Extended Data Fig. 4e). This new analysis complements the original quantification showing that 85% of Esr1-GFP$^+$ cells are LEPR$^+$.

- Line 237-247: It is indeed a paradigm-shifting discovery that estrogen receptor in LEPR+ cells, but not osteoblasts/cytes, is what makes E2-induced anabolism possible. The authors undertook an admirable task of carefully dissecting the lineage requirement.

RESPONSE: We thank the reviewer for this encouraging comment. We are pleased that the reviewer found the genetic dissection of the cellular site of E2–ERα action to be informative.

- Line 262: It should be noted and emphasized that OLN is not entirely responsible for E2-induced bone anabolism.

RESPONSE: We agree with the reviewer. We have revised the text to clarify that OLN contributes to, but does not fully account for, E2-induced bone anabolism. Specifically, E2 increased bone mass in both wild-type littermate controls and *Oln*$^{-/-}$ mice, although the response was attenuated in the mutants, supporting a contributory role for OLN while indicating that additional OLN-independent mechanisms also participate in the anabolic response to E2.

- Line 264: Sex of the CFU-F donor should be specified, both in mice and humans.

RESPONSE: We have provided the sex of the mouse and human CFU-F donors for each experiment in the Source Data tables. Where cultures from both sexes were included and pooled, this is indicated explicitly.

- Line 285: The authors should note that the Esr1-independent decline in bone mass is more significant than the Esr1-dependent one.

RESPONSE: We thank the reviewer for this helpful point. We agree that the *Esr1*-independent component of adult bone loss is larger than the *Esr1*-dependent component observed after *Esr1* deletion in LEPR$^+$ cells. We did not intend to imply that ERα signaling in LEPR$^+$ cells accounts for the entire age-associated decline in bone mass. We have revised the text to state that longitudinal microCT analyses revealed age-associated bone loss in both genotypes, with accelerated bone loss following *Esr1* deletion in LEPR$^+$ cells. This clarifies that ERα signaling in LEPR$^+$ cells contributes to adult bone maintenance but does not account for all adult bone loss.

- Line 295: The statement that the observed osteopenia is driven by impaired bone formation rather than increased resorption is interesting. Do the authors postulate that estrogen's direct action on osteoclasts is responsible for enhanced bone resorption in estrogen deficiency, rather than an indirect mechanism through the RANKL-RANK system?

RESPONSE: We thank the reviewer for raising this important mechanistic point. We agree that systemic estrogen deficiency is classically associated with increased bone resorption. Our statement was intended to refer specifically to the *Esr1* deletion in LEPR$^+$ cells and statin-treated models analyzed in this study, rather than to estrogen deficiency-induced osteoporosis in general. To address whether altered osteoclastogenic signaling from LEPR$^+$ cells contributes to these phenotypes, we analyzed *Rankl* and *Opg* expression in sorted LEPR$^+$ cells. *Esr1* deletion in LEPR$^+$ cells did not significantly increase *Rankl* expression or reduce

Opg expression in LEPR⁺ cells (new Extended Data Fig. 8e, f). Similarly, atorvastatin treatment in ovariectomized mice did not significantly alter *Rankl* or *Opg* expression in LEPR⁺ cells (new Extended Data Fig. 2i, j). We also quantified osteoclast number on trabecular bone surfaces in the relevant models (new Extended Data Fig 2k and new Extended Data Fig. 8g). These analyses, together with the serum CTX-1 measurements, did not reveal detectable changes in osteoclastogenic or bone-resorptive parameters that would explain the skeletal phenotypes observed after *Esr1* deletion or atorvastatin treatment.

We do not mean to imply that enhanced bone resorption after systemic estrogen deficiency is mediated exclusively by direct estrogen action on osteoclasts, or exclusively through an indirect RANKL-RANK mechanism. Rather, our data indicate that the phenotypes examined here are not associated with detectable changes in *Rankl* and *Opg* expression in LEPR⁺ cells or bone resorption. We have revised the text to clarify that systemic estrogen deficiency may increase bone resorption through mechanisms outside the LEPR⁺ cell population analyzed in this study.

- Line 309: How does a circulating OLN look in male patients as well?

RESPONSE: We thank the reviewer for this helpful suggestion. The human cohorts analyzed in this study were designed to examine statin-associated changes in postmenopausal women and did not include a matched male cohort with plasma samples suitable for circulating OLN analysis. We therefore cannot determine from the current human data whether circulating OLN is altered in male statin users. As an experimental complement, we analyzed aged male mice. In contrast to aged females, atorvastatin did not increase *Oln* transcription in LEPR⁺ cells or circulating OLN levels in aged males (new Extended Data Fig. 9e, f; previously Extended Data Fig. 7e, f). Future clinical studies will be needed to determine whether circulating OLN is regulated by statins in men.

- Figure 4h: Can the authors also show the graph with the male and female data separately?

RESPONSE: We thank the reviewer for pointing out this ambiguity. The experiment shown in Fig. 4h was performed in ovariectomized female mice, and male mice were not included in this experiment. We have revised the figure legend to make this clear.

- Figure 7e: The authors should clarify that these mice were ovariectomized.

RESPONSE: We thank the reviewer for this suggestion. We have revised the Fig. 7e legend and the corresponding Results text to clarify that the mice analyzed in this experiment were ovariectomized.

<u>Referee #4</u>:

Yang, Guo, Ye et al present a thorough examination of statin effects on bone mass using both human and mouse models. The data on the effects of statins on BMD and fracture risk

in humans is mixed, but they generally suggest there is a positive effect in at least some settings. These authors show that statins improve bone mass in mice, but only in settings of estrogen deficiency, and in two fairly small cohorts of postmenopausal women. They demonstrate that both statins and estrogen can promote osteogenic differentiation of LepR+ progenitor cells, but, somewhat surprisingly, the statins do not appear to affect osteoclast activity. They go on to show that ERalpha expression is enriched in LepR+ cells, and propose that osteolectin is an important modulator of both estrogen and statin activity, either through direct activation, or through Rac1-mediated NFkB signaling. This study is novel with robust approaches and the use of several novel mouse lines. Statistics appear appropriate for the most part, and while some results are overstated, the overall conclusions are appropriate.

Major comments
1. There are a lot of methodological details that are important to understand the results that are not covered in the results or figures. Figure 2 in particular is hard to understand, but in many cases for mouse studies it would be helpful to add an experimental outline diagram so it is clear when treatments and/or surgery are performed in relation to each other, or at least state this in the figure legend (such as timing of doxycycline, atorvastatin etc).

RESPONSE: We thank the reviewer for this helpful suggestion. We agree that the timing of surgery, doxycycline or tamoxifen induction, hormone or drug treatment, and endpoint analyses is important for interpreting the mouse experiments, particularly the fate-mapping studies in Fig. 2. We have therefore revised the Methods and, where appropriate, the relevant figure legends to state the experimental timelines more clearly. These revisions include the timing of doxycycline or tamoxifen administration, OVX or sham surgery, E2 pump implantation, atorvastatin or evolocumab treatment, and endpoint tissue collection for the atorvastatin/evolocumab treatment experiments, LEPR^{+} cell fate-mapping experiments, E2 and OVX experiments, and conditional *Esr1* and *Rac1* deletion experiments. These additions clarify the temporal relationships among induction, surgery, treatment, and analysis across the mouse studies.

2. The clinical data shown in Fig 1 should be introduced as a retrospective study. It is surprising that the control women in this age group do not really appear to be losing BMD. Other studies in this age group typically show some annual decrease (about 1% per year in doi: 10.1056/NEJMoa2407031). Is data from other sites than lumbar spine also available? The violin plots give a nice indication of the spread of the data, but the authors could consider showing the change per year as a %, and adding both change/year and % change to the table in the extended data.

RESPONSE: We thank the reviewer for these important suggestions. We have now explicitly described the human cohort analysis as a retrospective study in the Results and Methods. We have also revised Fig. 1a, b to present the annual percentage change in lumbar spine BMD. Both absolute annualized BMD change and annual percentage BMD change are included in the revised Extended Data Fig. 1a, b, together with the corresponding baseline and

menopause-related clinical variables. After re-expressing the data as annual percentage change, the control groups showed an annual decline in lumbar spine BMD that is consistent with the magnitude reported in prior longitudinal studies of postmenopausal women, including the study cited by the reviewer (NEJM 392:239). This revised presentation makes the longitudinal BMD changes and the differences among control participants, evolocumab users, and statin users clearer.

Longitudinal DXA measurements suitable for paired annualized analysis were consistently available across both cohorts for lumbar spine BMD. Comparable paired longitudinal measurements from other skeletal sites, including total hip or femoral neck, were not available to support a parallel analysis. We have revised the Methods to provide the beginning and end dates of cohort data collection for each hospital, the maximum interval between paired DXA scans, the definition of medication exposure, and the calculation of absolute and percentage annualized BMD change.

3. ssRNAseq analysis of bone marrow stromal populations have indicated some diversity in LepR+ cells, and suggest that they include both adipo-CAR (likely in the central marrow) and osteo-CAR (closer to the bone surface) cells. Can the authors reanalyze data from other studies to determine which cell subsets express osteolectin? The authors should at least acknowledge that the osteogenic effect of these treatments may be primarily on the more bone-adjacent cell populations rather than the central marrow cells that do not typically become osteoblasts unless there is an injury or other major perturbation (e.g. DOI: 10.1038/s41467-019-14029-w).

RESPONSE: We thank the reviewer for raising this important point. We agree that LEPR^{+} cells are heterogeneous and that our current genetic and fate-mapping approaches do not by themselves resolve which LEPR^{+} subpopulation mediates the estrogen- and statin-associated osteogenic response. To provide additional context, we re-analyzed a previously published single-cell RNA-seq dataset of bone marrow niche cell populations with annotated stromal subsets (Nature 569:222). This analysis showed that *Oln* and *Esr1* were expressed at higher levels in an overlapping LEPR^{+} cell subset that also expressed osteogenic markers, including *Sp7* and *Bglap*, rather than being uniformly expressed across all LEPR^{+} cells (new Extended Data Fig. 5k–n). We have revised the text to acknowledge LEPR^{+} cell heterogeneity and the model raised by the reviewer, including prior work suggesting that central marrow stromal cell populations and bone-adjacent osteogenic stromal populations can have distinct osteogenic potential under homeostatic and injury conditions (Nat Comm 11:332). Our analysis is consistent with the possibility that *Oln*-expressing LEPR^{+} cells overlap with osteogenic-biased or bone-adjacent subsets. However, because OLN is secreted and because our current genetic approaches target the broader LEPR^{+} population, the LEPR^{+} subset that is necessary and/or sufficient for the phenotype remains unresolved. Future subset-specific and spatially resolved approaches will be required to determine which LEPR^{+} subpopulation mediates the estrogen- and statin-induced bone anabolic effects.

4. The effect of statin treatment and estrogen deficienty in mice on osteoclasts is only ever

examined using CTX, which shows a relatively small change with OVX (Fig 1k). Given that the prenylation pathway is a well-established regulator of osteoclast activity, the authors should confirm if there are differences in trabecular or endocortical osteoclast numbers in these animals, and the conditional receptor knockout animals in Figure 5.

RESPONSE: We thank the reviewer for this important suggestion. We agree that, because the mevalonate/prenylation pathway is a well-established regulator of osteoclast function, plasma CTX-1 alone is not sufficient to assess whether statins or *Esr1* deletion alter bone resorption. We have therefore performed additional osteoclast analyses in the relevant models. Specifically, we quantified osteoclast number on trabecular bone surfaces in OVX mice treated with vehicle, atorvastatin, or evolocumab, as well as in *Esr1* cKO mice and littermate controls. These analyses showed that atorvastatin did not significantly reduce osteoclast number in OVX mice (new Extended Data Fig. 2k), and that *Esr1* deletion in LEPR$^+$ cells did not significantly increase osteoclast number on bone surfaces relative to controls (new Extended Data Fig. 8g). We also analyzed *Rankl* and *Opg* expression in sorted LEPR$^+$ cells. Neither *Esr1* deletion in LEPR$^+$ cells nor atorvastatin treatment in OVX mice significantly altered *Rankl* or *Opg* expression in LEPR$^+$ cells (new Extended Data Fig. 8e, f and new Extended Data Fig. 2i, j). Together with the CTX-1 measurements, these analyses indicate that the phenotypes examined here are not associated with detectable changes in bone resorption.

We have revised the Results and figure legends accordingly. Although statins can inhibit osteoclast-mediated bone resorption *in vitro*, prior work showed that this effect does not necessarily translate into inhibition of bone resorption *in vivo* (J Bone Miner Res 18:88–96). By contrast, nitrogen-containing bisphosphonates have high affinity for mineralized bone surfaces and are released during osteoclastic resorption, enabling selective uptake by resorbing osteoclasts and potent inhibition of small GTPase prenylation (Bone 42:848). Atorvastatin does not have this bone-mineral-targeting property. Thus, the absence of a measurable reduction in osteoclast number or CTX-1 in our OVX model is consistent with the conclusion that systemic atorvastatin treatment under these conditions primarily increases bone mass through enhanced osteogenic differentiation of LEPR$^+$ cells rather than through a detectable anti-resorptive effect.

5. The data showing enhanced in vitro differentiation in response to statins is insufficient (Fig 1l-o). Additional verification of enhanced osteogenic differentiation such as expression of marker genes should be shown, and ideally the mouse studies should be done in males and females.

RESPONSE: We thank the reviewer for this helpful suggestion. We agree that, although Alizarin Red staining provides evidence of matrix mineralization, osteogenic marker expression provides an important independent validation of enhanced osteogenic differentiation. We have therefore added quantitative gene-expression analyses in CFU-F cultures treated with atorvastatin. Atorvastatin increased the expression of osteogenic markers, including *Bglap*, *Ibsp*, and *Dmp1*, consistent with enhanced osteogenic differentiation (new Extended Data Fig. 2m, o). These additional marker gene analyses strengthen the conclusion

that atorvastatin promotes osteogenic differentiation of CFU-Fs. The mouse CFU-F experiments included cultures derived from both male and female mice. We have revised the figure legends and Source Data files to specify donor sex for the mouse and human CFU-F experiments. In these assays, we did not detect a consistent sex-dependent difference in osteogenic differentiation or in the response to E2 or atorvastatin. We therefore present the CFU-F data with male- and female-derived cultures combined, while providing donor-sex information in the Source Data tables.

6. I want to commend the authors for making a the tet-on LepR-Cre for this study. However can they please include some type of data to evaluate the recombination efficiency, and some idea of how well it recapitulates LepR-CreER. The % of osteoblasts labeled in Figure 2 sounds lower than the previous reports, although determining the timeframe for these studies is not straightforward (see comment 1).

RESPONSE: We thank the reviewer for this helpful comment and for recognizing the value of the Tet-On Lepr-Cre tool. We agree that additional validation of recombination efficiency and specificity is important for interpreting the fate-mapping experiments. We have now added new data evaluating Tet-On Lepr-Cre-mediated recombination after doxycycline administration. Flow cytometric analysis showed that approximately 80% of LEPR^{+} cells were labeled by tdTomato after doxycycline induction (new Extended Data Fig. 3c), whereas labeling of hematopoietic and endothelial cells was minimal (new Extended Data Fig. 3a, b). These data support efficient and specific recombination in the LEPR^{+} cell population.

We agree that the fraction of osteoblasts labeled in our study is lower at early chase time points than reported in some previous studies using Lepr-CreER (Cell Stem Cell 28:2122). We have revised the manuscript to clarify this point. Mature osteoblasts do not themselves express LEPR at appreciable levels (Nature 481:457; Bone Res. 13:6); therefore, osteoblasts labeled by Lepr-CreER or Tet-On Lepr-Cre reflect osteoblasts formed from LEPR^{+} cells labeled immediately after tamoxifen or doxycycline induction, respectively. Importantly, our Tet-On Lepr-Cre system was designed to enable tamoxifen-independent fate mapping, because tamoxifen is a selective estrogen receptor modulator with well-documented estrogen-agonist activity in bone and bone anabolic effects (Endocrinology 146:1060). Thus, the goal of this allele was to avoid tamoxifen-associated skeletal confounding in studies of estrogen signaling and bone formation, rather than to maximize early osteoblast labeling efficiency.

Indeed, the labeling efficiency of Tet-On Lepr-Cre and Lepr-CreER in LEPR^{+} cells was comparable immediately after doxycycline or tamoxifen induction, respectively, with approximately 80% labeling in both systems. Thus, the lower fraction of labeled osteoblasts at early chase time points in the Tet-On Lepr-Cre system does not reflect inefficient labeling of LEPR^{+} cells themselves. Rather, we interpret this difference as reflecting the absence of tamoxifen-associated stimulation of bone formation in the doxycycline-inducible Tet-On Lepr-Cre system. In Lepr-CreER fate-mapping experiments, tamoxifen induction labels LEPR^{+} cells at the same time that tamoxifen can stimulate bone formation, thereby increasing the apparent early contribution of labeled LEPR^{+} cells to newly formed osteoblasts. By contrast, Tet-On

Lepr-Cre labels LEPR$^+$ cells without this tamoxifen-associated osteogenic stimulus, resulting in lower early osteoblast labeling despite comparable labeling of the LEPR$^+$ stromal population itself.

To further evaluate how well Tet-On Lepr-Cre recapitulates the long-term osteogenic output reported for Lepr-CreER, we added a longitudinal fate-mapping analysis following doxycycline induction at 2 months of age. Consistent with previous Lepr-CreER studies (Cell Stem Cell 28:2122), Tet-On Lepr-Cre-labeled LEPR$^+$ cells persisted during adulthood and progressively contributed to osteoblasts and osteocytes over time, with approximately 54% of osteoblasts derived from labeled LEPR$^+$ cells at the longest chase time point (new Extended Data Fig. 3c, d). These data show that Tet-On Lepr-Cre captures the long-term osteogenic contribution of LEPR$^+$ cells while avoiding tamoxifen exposure.

Finally, because all experimental groups in our statin and estrogen studies were analyzed using the same doxycycline induction and chase strategy, the relative changes in LEPR$^+$ contribution to osteoblasts and osteocytes remain interpretable despite the lower early osteoblast labeling in the Tet-On Lepr-Cre system. We have added these validation data and clarified these points in the revised Results and figure legends.

7. For the studies looking at LepR-Tomato contribution to GFP+ osteoblasts in Figure 2, quantification of trabecular and endocortical surfaces should also be performed using the histology as these compartments can have different responses to estrogen deficiency and contributions of LepR cells.

RESPONSE: We thank the reviewer for this important suggestion. We agree that trabecular and endocortical bone surfaces can respond differently to estrogen deficiency and may receive different contributions from LEPR$^+$ cells. We have now performed separate histological quantification of tdTomato$^+$ contribution to surface-associated Col1a1*2.3-eGFP$^+$ osteoblasts at trabecular and endocortical surfaces in the relevant fate-mapping experiments. These analyses showed that changes in osteoblast formation from LEPR$^+$ cells were generally more pronounced at trabecular surfaces. In ovariectomized mice, atorvastatin increased the tdTomato$^+$ contribution to Col1a1*2.3-eGFP$^+$ osteoblasts primarily at trabecular surfaces. E2 increased LEPR$^+$-derived osteoblast formation at both trabecular and endocortical surfaces, with a stronger effect at trabecular surfaces (new Extended Data Fig. 3l, m). Conversely, ovariectomy reduced osteoblast formation from LEPR$^+$ cells most prominently at trabecular surfaces, with a similar but less pronounced reduction at endocortical surfaces (new Extended Data Fig. 3e, f). We have revised the Results and figure legends to describe these analyses.

8. Why do the CUT&Tag studies in Figure 4 lack an untreated control? ERalpha may be able to perform ligand independent binding to some sites.

RESPONSE: We thank the reviewer for this important point. We agree that ERα can exhibit basal or ligand-independent occupancy at some genomic sites, and that comparison with a vehicle-treated control is important for interpreting E2-induced ERα binding. We have

therefore added vehicle-treated controls to the ERα CUT&Tag analysis in murine and human CFU-Fs. We have also expanded the Methods to specify the culture conditions used for these experiments. Vehicle- and E2-treated cells were cultured under the same estrogen-depleted conditions, using phenol red-free medium supplemented with charcoal-stripped serum before treatment. These conditions were used to reduce background estrogenic activity from the culture medium and serum, consistent with prior studies showing that phenol red can have weak estrogenic activity and that charcoal stripping reduces steroid hormones in serum (PNAS 83:2496, Endocrinology 157:3760). The new data show that ERα occupancy at the upstream mouse *Oln* and human *OLN* regulatory regions was minimal under vehicle-treated conditions and was strongly increased after E2 treatment (revised Fig. 4i, j). This upstream E2-induced ERα-bound region corresponds to the regulatory element tested in our reporter analysis, supporting an E2-dependent regulatory function at this genomic site. By contrast, the ERα signal within the *Oln*/*OLN* gene body did not show an E2-dependent increase. We therefore focused on the upstream E2-induced ERα-bound region as the candidate regulatory element mediating *Oln*/*OLN* transcriptional activation. We have revised the Results, Methods, and figure legends to clarify the inclusion of vehicle-treated controls, the estrogen-depleted culture conditions, and the conclusion that E2 specifically increases ERα occupancy at the upstream regulatory region relative to vehicle-treated controls.

Minor comments:
1. CFU-F is used to describe many of the culture experiments, and while it sounds like they are grown initially as CFU-F, they are then pooled and reseeded for many of the assays. Perhaps the authors could rename to passage 1 bone marrow CFU-F or something like that.

RESPONSE: We thank the reviewer for this helpful suggestion. We have revised the Methods to clarify that bone marrow cells were first plated under CFU-F conditions and that the resulting passage 1 CFU-F-derived cultures were pooled and reseeded for osteogenic differentiation, gene expression, and pharmacological assays. Thus, the functional assays were performed using passage 1 CFU-F-derived cultures rather than individual primary CFU-F colonies.

2. Line 126: this title does not accurately describe the results in this section.

RESPONSE: We thank the reviewer for pointing this out. We agree that the original organization could make the section title appear premature, because the direct *in vivo* fate-mapping evidence for statin-induced osteogenic differentiation of $LEPR^+$ cells was presented at the beginning of the following section. We have therefore reorganized the Results so that the statin fate-mapping data are now included in this subsection. The section now contains the bone-formation/resorption analyses, CFU-F osteogenic differentiation assays, and direct Tet-On Lepr-Cre fate-mapping evidence showing that atorvastatin promotes osteogenic differentiation of $LEPR^+$ cells under estrogen-deficient conditions.

3. Line 176-177 (and several other places): Almeida et al 2013 (and a review by similar authors in 2017) suggests that a lot of the activity of estrogen on osteogenesis is targeting

earlier stages of the lineage since they see essentially no phenotype in their mature osteoblast model. Please tone down these statements.

RESPONSE: We thank the reviewer for this important clarification. We agree that prior work, including studies by Almeida and colleagues (J Clin Invest 123:394, Physiol Rev 97:135), has suggested that estrogen action on bone formation may not be confined to mature osteoblasts or osteocytes and may involve earlier stages of the osteogenic lineage. We have revised the text to avoid implying that this general concept was unknown. Instead, we now clarify that the unresolved question addressed by our study is whether E2–ERα signaling directly regulates osteogenic differentiation of adult bone marrow skeletal stem/progenitor cells. By combining tamoxifen-independent Tet-On Lepr-Cre fate mapping, $LEPR^+$ cell-specific *Esr1* deletion, and osteoblast/osteocyte-restricted *Esr1* deletion using Col1a1-CreER, our study supports an important contribution of ERα signaling in $LEPR^+$ cells to E2-induced osteogenic differentiation and bone anabolism. We have revised the relevant Results and Discussion text to tone down our original statements.

4. Extended Figure 4, Extended Figure 6 and associated results: can further cortical parameters be shown such as the perimeter, or the total area and marrow area for all these studies. It would be useful to understand how much the estrogen-related changes are mediated by periosteal vs endosteal expansion.

RESPONSE: We thank the reviewer for this helpful suggestion. We have now added additional cortical microCT parameters, including cortical area and cortical area/total area, for the studies shown in new Extended Data Fig. 5d, e, i, and j, new Extended Data Fig. 6h, i, and new Extended Data Fig. 8c, d, l, and m, and described these analyses in the revised figure legends. These additional cortical analyses showed that the cortical phenotypes were more modest and less consistent than the trabecular phenotypes, and did not identify a dominant periosteal or endosteal expansion pattern that explained the estrogen-related skeletal changes. This supports our interpretation that the major skeletal effects in these models are detected primarily in trabecular bone, whereas cortical geometric changes are comparatively limited.

5. The official gene symbol for osteolectin should be mentioned when it is introduced

RESPONSE: We thank the reviewer for this suggestion. We have revised the text to introduce osteolectin together with its official gene symbol, *Clec11a*, at first mention.

6. Line 260-261/interpretation of Figure 4h: this knockout mouse clearly has a pre-existing phenotype. They still have a substantial response to estrogen, so it is difficult to justify this statement

RESPONSE: We thank the reviewer for this important point. We agree that $Oln^{-/-}$ mice have a pre-existing skeletal phenotype and retain a substantial response to E2 treatment. We have therefore revised the interpretation of Fig. 4h to avoid implying that OLN fully accounts for E2-

induced bone anabolism. We now state that E2 increased bone mass in both wild-type littermate controls and *Oln*$^{-/-}$ mice, although the response was attenuated in the mutants. Thus, OLN contributes to the full anabolic response to E2, but the residual E2 response in *Oln*$^{-/-}$ mice indicates that OLN-independent mechanisms also participate.

7. Osteolectin levels in different conditions: for extended Fig 7c, can plasma levels from the sham group be included for reference?

RESPONSE: We have now added plasma OLN levels from sham-operated mice as a reference group in Extended Data Fig. 9c (previously Extended Data Fig. 7c), and revised the figure legend accordingly.

8. Methods: For the retrospective human study, please include additional information about how the participants were chosen, the beginning and end dates of the study cohort data, and the maximum amount of time between Dexa scans.

RESPONSE: We thank the reviewer for requesting these methodological details. We have revised the Methods to explicitly state that the human BMD analysis was retrospective and to describe how participants were selected. We have added the beginning and end dates of cohort data collection for each hospital, the inclusion and exclusion criteria, the definition of medication exposure, and the maximum interval between paired DXA scans used to calculate annualized BMD change. These details are now provided in the Methods and summarized where appropriate in Extended Data Fig. 1a, b.

9. Methods: The human studies with serum collection appear to be different patients than for the dexa study, please state how these patients were recruited, and if there were any differences in inclusion/exclusion criteria.

RESPONSE: We thank the reviewer for this helpful comment. We have clarified in the Methods that the plasma samples used for OLN measurements were obtained from a separate cohort of postmenopausal participants recruited from the same hospitals. The plasma cohort used the same general inclusion and exclusion criteria as the retrospective DXA cohort, except that plasma availability was required and paired longitudinal DXA measurements were not required. We have revised the Methods accordingly.

10. Methods: how the human marrow samples were collected and cultured is not covered, not are any of the demographics of these patients.

RESPONSE: We thank the reviewer for this suggestion. We have revised the Methods to clarify that human bone marrow samples were obtained during total hip replacement surgery, enzymatically dissociated immediately after collection, and plated directly under the same CFU-F culture conditions used for murine bone marrow. LEPR^{+} cells were prospectively isolated by flow cytometry only for the indicated sorting experiments. We have also provided the available donor demographic information, including age and sex, in the

Source Data tables. These revisions clarify the origin, processing, and culture of the human marrow samples used for CFU-F assays, $LEPR^+$ cell sorting, and downstream analyses.

11. Methods line 864: please elaborate on what 'freshly dissociated' means, is this flushed marrow, or enzymatically dissociated marrow/endosteum?

RESPONSE: We thank the reviewer for pointing this out. Freshly dissociated bone marrow cells refer to bone marrow cells processed immediately after collection and analyzed or cultured without prior expansion. In the experiments described here, this referred to enzymatically dissociated bone marrow cells used directly for CFU-F culture or flow cytometry.

12. Methods: flow cytometry is described for flushed bone marrow, but how were samples where Col1a1-GFP was analyzed prepared?

RESPONSE: We thank the reviewer for this request for clarification. We have revised the Methods to specify how samples were prepared for flow cytometric analysis of Col1a1*2.3-$eGFP^+$ cells. For these analyses, crushed bone fragments were enzymatically dissociated to recover bone-associated osteoblasts before flow cytometry. We have now clarified this procedure in the Methods.

13. Figure 1a-b and Fig 6a-b - please include the n for each group in either the figure or the legend.

RESPONSE: We have revised the figure legends to include the n numbers for each group in Fig. 1a, b and Fig. 6a, b.

14. Figure 4f-g data: can the more extended list of differentially expressed genes be provided as supplemental data. Are Rankl and Opg regulated as would be expected from previous reports?

RESPONSE: We thank the reviewer for this helpful suggestion. We have now provided the extended list of differentially expressed genes from the $LEPR^+$ cell RNA-seq analyses in the Source Data table for Fig. 4f, g. We also specifically examined *Rankl* and *Opg* expression in these datasets. In $LEPR^+$ cells, E2 treatment increased transcription of both *Rankl* and *Opg*, whereas OVX did not significantly alter either transcript (revised Fig. 4f, g). In additional experiments relevant to the statin and *Esr1* conditional knockout phenotypes, *Esr1* deletion in $LEPR^+$ cells did not significantly alter *Rankl* or *Opg* expression (new Extended Data Fig. 8e, f), and atorvastatin treatment in ovariectomized mice did not significantly alter *Rankl* or *Opg* expression in $LEPR^+$ cells (new Extended Data Fig. 2i, j). Together with the CTX-1 measurements and new osteoclast analyses, these data indicate that the skeletal phenotypes observed in the *Esr1* conditional knockout and statin-treated ovariectomized mice are not associated with detectable changes in *Rankl* and *Opg* expression in $LEPR^+$ cells or bone resorption. We have revised the Results and text accordingly.

Figure 4i-j and 7h-I (cut and tag data) - can some sort of scale be included on both axes of these plots.

RESPONSE: We have added genomic coordinate and signal-intensity scales to the CUT&Tag tracks shown in Fig. 4i, j and Fig. 7h, i.

16. Figure 7 western blots: quantification should be included somewhere for these. For b in particular the difference is subtle.

RESPONSE: We thank the reviewer for this suggestion. We have now added quantification of the C15AlkOPP-labeling data shown in Fig. 7b in new Extended Data Fig. 10e. The quantified data are presented from three independent biological replicates. We have also provided all independent repeats of the representative western blots and in-gel fluorescence images shown in Fig. 7 as original uncropped blots and gels in the Source Data files.

17. Ref 48 should be PubMed ID:11771662

RESPONSE: We thank the reviewer for identifying this error. We have corrected the cited reference to the article corresponding to PubMed ID 11771662 (J Bone Miner Res 17:15).

References
A lot of the references are quite old, particularly the clinical references, which partly reflects that the high quality work in this field was mostly published some time ago. However, with regards to the clinical evidence, there are several meta analyses, at least one of which would be appropriate to cite.

RESPONSE: We thank the reviewer for this helpful suggestion. We have updated the clinical discussion by citing more recent clinical evidence, including the JUPITER randomized clinical trial analysis (JAMA Intern Med 175:171) and a recent meta-analysis of statin use and fracture risk (J Am Med Dir Assoc 20:1566). We have revised the Discussion to note that clinical studies of statins and skeletal outcomes have yielded variable results, with observational associations in some cohorts but no consistent fracture-preventive effect in randomized or meta-analytic studies. We therefore emphasize that our human cohort data are observational and that our study provides a mechanistic framework for context-dependent skeletal responses to statins, rather than evidence that statins should be used clinically as osteoporosis or fracture-prevention therapies.

We hope that these new data, additional analyses, and clarifications adequately address the reviewers' concerns, improve the manuscript, and render it suitable for publication.

Sincerely,

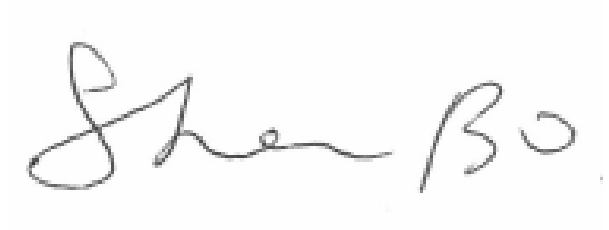

Bo Shen, Ph. D.
Assistant Investigator, National Institute of Biological Sciences, Beijing (NIBS)
Assistant Professor, Tsinghua Institute of Multidisciplinary Biomedical Research

Sep 8, 2026

Qingzhong Ren, PhD
Senior Editor, Nature Aging

Dear Qingzhong,

Thank you for the positive in-principle decision on our manuscript. We have added the requested images, clarifications and revisions to address all of the reviewers' remaining comments. Below is a point-by-point response in which the reviewer comments are presented in full in italics, followed by our responses in normal type.

Referee #3:

It is admirable that the authors returned such an extensively revised manuscript in a very short amount of time. In response to my critiques, the authors provided several important pieces of supporting evidence for the revised manuscript, including 1) a lack of statin-induced anabolic responses in bone in orchiectomized males, 2) unaltered RANKL and OPG in Esr1-deficient LepR+ cells, and 3) increased bone marrow adipocytes in estrogen-deficient mice rescued by statins. Overall, this remains an extremely well-conducted study demonstrating the compensatory actions of statins in estrogen-deficient LepR+ cells. A few minor suggestions are listed below.

1. Abstract, Line 40-42. The authors should clarify that atorvastatin's anabolic effect can only be observed in estrogen deficiency.

RESPONSE: We thank the reviewer for this helpful suggestion. We have revised the Abstract to clarify that atorvastatin promotes bone anabolism specifically under estrogen-deficient conditions.

2. Abstract, Line 45. As I pointed out in the initial round of peer review, it is not entirely accurate that estrogen and statins "converge" to promote bone anabolism. A more accurate description would be that statins "compensate" for the signaling defect induced by estrogen deficiency through mechanistically distinct routes.

RESPONSE: We agree. We have replaced the description of convergence in the Abstract with the following statement: "Thus, statins compensate for reduced E2–ERα signaling through a mechanistically distinct pathway."

3. Additional results on bone marrow adipocytes are greatly appreciated. It would be important to show the images of bone marrow adipocytes in each group. How was the adipocyte number determined? Did the authors use H&E or immunostaining?

RESPONSE: Bone marrow adipocytes were identified as perilipin^{+} cells by immunofluorescence staining of bone sections and counted throughout the metaphyseal bone

marrow. We have clarified this method in the revised manuscript and added representative images used for quantification to new Extended Data Fig. 2m and Extended Data Fig. 8h.

Referee #4:

The authors have done a good job addressing most comments. Addition of the data establishing efficiency of the Tet-On LepR model is a really helpful addition technically, and as the authors point out, hints at confounding results from the use of tamoxifen.

Two of my comments were not fully addressed as outlined below, which could be rectified by minor changes to text and/or figures.

Major comment 1

The authors provided a little more detail in the methods section, but not a diagram in the figures or outline of the study design in the figure legend which was what was requested. It would be good to include this at least for Figure 2.

RESPONSE: We thank the reviewer for pointing this out. We have now added schematic diagrams outlining the study designs in Fig. 2a, Fig. 2d, Fig. 2i and Extended Data Fig. 3g.

Major comment 7

Thank you for including the histological evaluation, but the results should mention the compartment-specific data in the text at least for Fig S3e-f where the effect on trabecular bone predominates.

RESPONSE: We have revised the Results to incorporate these compartment-specific data and now state that the atorvastatin-induced increase in osteoblast formation from LEPR$^+$ cells was more pronounced at trabecular than at endocortical bone surfaces (Extended Data Fig. 3e, f).

We hope that these clarifications adequately address the reviewers' remaining comments and render the manuscript suitable for publication.

Sincerely,

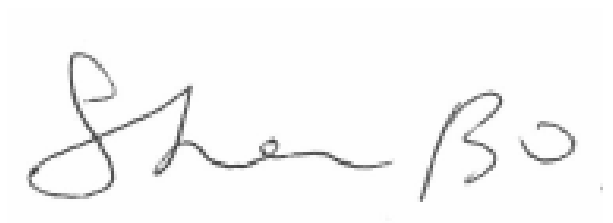

Bo Shen, Ph. D.
Assistant Investigator, National Institute of Biological Sciences, Beijing (NIBS)
Assistant Professor, Tsinghua Institute of Multidisciplinary Biomedical Research
